# Yield and yield component trait analysis with DArT genotyping for GWAS in soybean grown in drought conditions of Kazakhstan

**DOI:** 10.3389/fpls.2025.1674201

**Published:** 2025-10-14

**Authors:** Aigul Amangeldiyeva, Raushan Yerzhebayeva, Shynar Mazkirat, Svetlana Didorenko, Sholpan Bastaubayeva, Bekzhan Maikotov, Rinat Kassenov, Assel Jenisbayeva, Yuri Shavrukov

**Affiliations:** 1 Kazakh Research Institute of Agriculture and Plant Growing, Almalybak, Kazakhstan; 2 Farabi University, Almaty, Kazakhstan; 3 College of Science and Engineering, Biological Sciences, Flinders University, Adelaide, SA, Australia

**Keywords:** bulk segregant analysis (BSA), candidate genes verification, Diversity array technology (DArT), drought, field trial, gene expression, genome-wide association study (GWAS), molecular genetic dendrogram

## Abstract

Development of drought tolerant cultivars of soybean is the single best way to address the challenge of global climate change and very limited water resources for crop irrigation in Central Asia including Kazakhstan. A set of 188 soybean cultivars with diverse origins was assessed for genome-wide association study (GWAS) for yield and eight yield-related traits in both irrigated (well-watered, WW) and non-irrigated (drought) conditions during 2 years in field trials in South-Eastern Kazakhstan. The 295K Diversity array technology (DArT) analysis was applied, and 16K filtered DArT markers were used for genotyping of 183 soybean accessions. In the results, 41 quantitative trait nucleotides (QTN) were identified as significantly associated with nine studied traits. To verify these results, bulk segregant analysis (BSA) was carried out in six breeding lines originating from two crosses between high-yielding under drought cvs, Sponsor and Zen, with drought sensitive cv Lastochka. The evaluation of combined results revealed 10 most significant QTN and eight most promising putative candidate genes, which were selected and tested for their gene expression using RT-qPCR under drought compared with WW controls. Among them, glucose-6-phosphate isomerase (*G6PI*), pentatricopeptide repeats (*PPR*) protein, and *ABC* transporter, associated with seed yield, seed weight per plant, and plant height, were highly upregulated in drought tolerant genotypes. In contrast, two other genes, *Rab*-GDP dissociation inhibitor (*Rab-GDI*) and Transducin with WD40 repeats, associated with seed yield, showed repression in the same genotypes. These verified genes involved in the control of yield and yield-related traits can be used for marker-assisted selection to develop novel genotypes and new soybean cultivars tolerant to strong drought in Kazakhstan and in other countries with similar conditions.

## Introduction

Soybean [*Glycine max* (L.) Merr.] is a very important crop for food and cattle feed, with growing consumption and high demands for soybean production worldwide (Boerema et al., 2016). This crop remains one of the largest sources of vegetable oil and highest protein content among all other food crops (Pagano and Miransari, 2016). Soybean can grow in diverse environmental conditions but suffers from various abiotic stresses, particularly drought (Hasanuzzaman et al., 2016). Drought is one of the biggest challenges in soybean production that can lead to dramatic yield reductions and even total crop failure (Dubey et al., 2019). Many different countries, including the USA (Nguyen et al., 2023) and China (Wang et al., 2020), have experienced these losses, and global climate warming will only escalate the threat for future soybean production (Yoneyama et al., 2025). Advanced breeding tools can help to identify drought-adaptive responses in soybean plants to enable the identification of essential and important genes involved in drought resilience (Jan et al., 2025).

Kazakhstan, with its arid climate, has limited rainfall and very few options to increase its water allocation to agriculture due to the vulnerability of the Central Asian countries to the effects of climate change (Zhiltsov et al., 2018). Therefore, in Kazakhstan, soybean growing is located only in irrigated lands of the southern regions, Zhetysu and Almaty, with 83% of total soybean production (Didorenko et al., 2025). Water limitation remains the biggest key problem for expanding the soybean crop in this country (Yerzhebayeva et al., 2024).

Drought tolerant soybean is the only way to address these water limits and climatic changes. Therefore, all new soybean cultivars must have a high level of drought resilience to support their sufficient profitability and competitiveness (Ferreira et al., 2024). However, tolerance to drought is a complex quantitative trait under polygenic control. Currently, due to the development of genomic technologies, significant progress is being made in the study of genetic control and mechanisms of resilience to drought stress (Dubey et al., 2019; Ali et al., 2022).

There are hundreds (if not thousands) of genes involved in the reaction of soybean plants to drought. This is not surprising because plants need global and urgent reorganisation of their overall metabolism to defend against the dehydration threat. For example, the pentatricopeptide repeat (PPR) gene family is very big and with diverse functions. In *Arabidopsis*, PPR genes were shown to have RNA-binding activity in chloroplasts and mitochondria, supporting their stability and significantly improving tolerance to drought and other abiotic stresses (Yuan and Liu, 2012; Jiang et al., 2015; Liu et al., 2016a). Nitrogen transporter, *NRT*, was found to be extensively involved in a signalling system with NO_3_^−^ transport in plants and to reduce stomata opening in response to abiotic stresses, including drought (Fang et al., 2021; Nedelyaeva et al., 2024). A gene regulation system with *Rab*-GTPase and *Rab* GDP dissociation inhibitor regulates vesicular transport and membrane trafficking in plant cells (Hála et al., 2005) and is strongly involved in the regulation of plant tolerance to drought and other environmental stresses (Liu et al., 2015; Martín-Davison et al., 2017). Another group of *Transducin* genes with WD40 repeats was recognised as containing key regulators of cell division and cytokinesis, involved in meristem organisation and plant-specific developmental stages during flowering and floral development (van Nocker and Ludwig, 2003). Additionally, it was reported that *HOS15*, the *Arabidopsis* trunsducin-WD40 repeats gene, repressed downstream genes and improved plant tolerance to abiotic stress like cold through histone deacetylation (Zhu et al., 2008), whereas WD40 repeat gene *MSI1* negatively regulated responses to drought stress in *Arabidopsis* plants (Alexandre et al., 2009). Of course, this is only a tiny portion of the genes and genetic networks related to drought tolerance in plants, but it must be emphasised for the purpose of this study.

Genome-wide association study (GWAS) is one of the advanced methods used in the identification of genomic regions associated with many economically valuable traits of agricultural crops, and it has seen continued active development in the last few years (He and Gai, 2023; Supritha et al., 2025). GWAS does not require a hypothesis about the origin or mechanism of trait development but is instead based on correlations between the trait phenotypes and molecular differences in the entire genome (Shook et al., 2021). GWAS technology is successfully realised in QTL (quantitative trait loci), which are closely associated with important traits in soybean plants, for example for seed quality (Potapova et al., 2025). Additionally, in many GWAS with SNP (single-nucleotide polymorphism), QTN (quantitative trait nucleotides) with the same function as QTL are successfully used for association analysis between studied traits and genetic regions close to or inside candidate genes (Lee et al., 2014; Zhang et al., 2024). Finally, the identification of candidate genes and development of linked molecular markers are the most important steps for marker-assisted selection (MAS) and the improvement of drought tolerance in crops, including soybean (Ali et al., 2022).

Many QTL–QTN, candidate genes and the corresponding linked molecular markers have already been identified for tolerance to drought and dehydration in plants of both cultivated and wild soybean (*G. max* and *G. soja*, respectively), and they are dispersed over many chromosomes and genetic regions. For example, the rate of seed germination after treatment with polyethylene glycol, PEG-6000 solution, was determined in panels of cultivated and wild soybean accessions with diverse origin with SNP, candidate genes, and markers identified in GWAS (Liu et al., 2020; Aleem et al., 2024; Jia et al., 2024; Nguyen et al., 2024). SNP markers and three potential candidate genes were found to be linked with yield-related traits in a GWAS study of soybean plants in a field trial under drought (Li et al., 2023; Liu et al., 2023; Zhang et al., 2024).

Bulked segregant analysis (BSA) is often used in experiments (Majeed et al., 2022), including soybean study under drought (Li et al., 2022). In this method, DNA extracted from plants in groups with contrasting phenotypes are combined in separated bulk samples or “bulks” for further analyses. Molecular profiles of these bulks can then provide differences in genes potentially associated with traits causing the contrasting phenotypes. Depending on experimental conditions, the number of selected and bulked genotypes can vary from tens to hundreds, as often happened with BSA in soybean (Sreenivasa et al., 2020) and in other legumes like lentil (Singh et al., 2016).

The identified candidate genes then have to be verified for their involvement and role in plant response to drought. The simplest and most popular approach is to study the expression of candidate genes using RT-qPCR in separate experiments under drought in comparison with well-watered plants in controlled conditions (Le et al., 2011; Chen et al., 2014).

Drought can have very different characteristics, including time, duration, severity, and repeated occurrence as “waves,” and the effect of drought on plants, including soybean, can depend on many additional factors such as geographic location, environmental conditions, plant stage, and the tolerance or sensitivity of studied soybean genotypes. Therefore, experiments for QTL and identification of candidate genes involved in drought tolerance must be carried out in each ecological zone or specific environment to determine the most suitable soybean genotypes.

The aims of this study were as follows: (1) phenotyping of 188 soybean accessions with diverse origins in a 2-year field trial experiment in South-Eastern Kazakhstan both in well-watered (irrigated) and drought (non-irrigated) conditions; (2) genotyping of the soybean set using a 295K DArT application; (3) QTN and candidate gene identification for yield and yield-related traits using GWAS analysis; (4) verification of the identified QTN and candidate genes with BSA analyses of hybrid breeding lines and their parents using the same 295K DArT approach; (5) expression analysis of candidate genes under drought in controlled greenhouse conditions.

## Materials and methods

### Plant material

A soybean germplasm collection with 188 cultivars was used in this study. The studied accessions represented all six maturity groups, from MG00 to MG4, with very diverse origins from 20 countries around the world. The studied soybean cultivars were selected based on their previous studies in well-irrigated and drought conditions in 2018–2020 and 2021–2022 (Didorenko et al., 2020; Yerzhebayeva et al., 2024).

Seeds of the selected soybean accessions were obtained from several Genebanks and Germplasm collections of Research Institutes, including the Kazakh Research Institute of Agriculture and Plant Growing, KRIAPG (Almaty region, Kazakhstan); the Genebank of Vavilov Research Institute of Plant Genetic Resources (St.-Petersburg, Russia); V.Pustovoit All-Russian Research Institute of Oilseeds (Krasnodar, Russia); Krasnoyarsk Research Institute of Agriculture (Krasnoyarsk, Russia); Soybean Research Institute (Poltava region, Ukraine); V.Yuriyev Plant Production Institute (Kharkiv, Ukraine); Agro-corporation ‘Soya Sever’ (Minsk region, Belarus); and the US National Plant Germplasm System (USDA, Urbana, IL, USA). The full list of 188 studied soybean cultivars is present in **Supplementary Material 1**.

Additionally, for hybrid analysis, two mapping populations were developed from crosses between local and foreign soybean cultivars, as described in the separate section below.

### Field experiments and design, well-irrigated, and drought

Field experiments with soybean cultivars were carried out at KRIAPG in the period 2023-2024. The field site was in the hill zone of South-Eastern Kazakhstan (Almaty region), at an altitude of 740 m above sea level, 43°15′ N and 76°54′ E. An image of the field trial is present in **Supplementary Material S2**.

Experiments were carried out in two areas simultaneously, with and without irrigation (control and drought, respectively). Four smart soil moisture sensors, Moisture-10 HS and Model S-SMx-M005 (Bourne, MA, USA), were installed to monitor volumetric water content (VWC) at a depth of 10 cm in all treatments at 1-min intervals. HOBO USB Micro Station data loggers (Bourne, MA, USA) were installed above the crop fields. Water availability was calculated in mm, as the sum of available soil moisture from irrigation and seasonal precipitation (from seed sowing till physiological maturity of plants). Drip irrigation equipment was installed at the water-supplying station. Drip tapes were placed in rows at approximately 15 cm from plants. The distance between the emitters was 20 cm. Watering was carried out from June 15 to August 25, with each watering occurring at 7-day intervals for a duration of 16 h. Emitters produced 1.6 L per hour, a total volume of 12 m^3^/ha per hour (Yelnazarkyzy et al., 2019). The total moisture supply per hectare in well-watered irrigated controls was in the range of 4,932-5,872 m^3^/ha, whereas it reached 2,820-3,760 m^3^/ha without irrigation (drought).

All 188 soybean cultivars were tested in 5-m^2^ four-row plots, with a density of 60 plants per m^2^. For hybrid population analysis, six breeding lines were tested in 0.25-m^2^ single-row plots, and with 30 cm between rows in both field trials. The field test was carried out in triplicate with a completely randomised plot design, with irrigation (designated as control, well-watered, WW) and without irrigation (designated as drought or drought stress, DS). The setup of experiments, all agronomic procedures, and treatments were carried out the same and on the same day in fields with and without irrigation.

### Meteorological conditions of the experimental field trial

According to Köppen’s classification (Beck et al., 2023), the climate of the Almaty region is “Dfa,” which can be described as continental, with hot summers. The average annual temperature is 6.5°C, and the amount of precipitation for the entire season reaches 891 mm. The soils are light chestnut, and the total humus content in the arable layer is low, ranging from 1.6% to 1.9%. The soil is slightly alkaline with a pH of 7.8, and the content of clay particles reaches 34.9% (Amangaliev et al., 2023).

Meteorological conditions during the study period (precipitation and average air temperature) were collected by an automatic weather station, iMetos (Model IMT300USW, Pessl Instruments, Weiz, Austria), located 300 m from the experimental site. Compared with 386.5 mm as long-term previous observations (1991-2020), the growing season of 2023 was characterised as extremely dry (282.2 mm), whereas precipitation in 2024 (376.8 mm) was close to the level of the long-term average (**Table 1**). The average monthly air temperature during the soybean growing season (April–September) in 2023 and 2024 exceeded the long-term average by 1.6°C and 1.3°C, respectively. The air temperature during the hottest month of July exceeded the long-term average value of 23.7°С. The hydrothermal coefficient (HTC) was 0.67 in 2023 and 0.84 in 2024.

**Table 1:** Weather conditions during the soybean growing season in the years 2023 and 2024.

Year	April	May	June	July	August	September	
Precipitation, mm							Total precipitation
2023	68.2	43.4	4.3	33.6	72.9	59.8	282.2
2024	111.3	121.2	19.7	85.2	25.1	14.3	376.8
*Long-term average*	110.6	98.4	59.9	56.9	34.8	25.9	386.5
Air temperature,°C							Average value
2023	11.8	17.2	24.6	27.1	24.5	17.7	20.5
2024	12.8	17.6	24.5	25.0	25.9	15.1	20.2
*Long-term average*	11.5	16.7	21.2	23.7	22.9	17.5	18.9

### Trait phenotypes and field evaluations

At the harvesting stage, the yield and yield components were assessed as described in the methodological recommendations published earlier (Vishniyakova et al., 2018). The yield of soybean seeds from plots, designated as “Yield” (Y), was determined in the phase of fully matured plants when seed moisture content reached 12%. Soybean plots were harvested by a Seedmech GmbH Classic Plus combine (Wintersteiger, Ried im Innkreis, Austria). The collected seeds were weighed using a CAS EC-6 electronic scale (CAS, Seoul, South Korea).

Additionally, 10 plants were randomly selected from each plot for each genotype and studied individually. Plant height (PH) was measured at the full maturity stage (R8) using a ruler, from soil level to the end of the longest stem, whereas the distance from soil level to the first internode with pods was measured for height to first pod (HFP). The same plants were used to count the number of side branches (NSB), number of productive nodes (NPN), and pod number per plant (PNP), and to measure seed weight per plant (SWP). Thousand seed weight (TSW) was measured using a Digital Automatic Seed Counter (ASC-TCP, Infitek, Shandong, China) for seed number, and a laboratory scale with two decimals accuracy (RV3102, Ohaus Adventurer, Shanghai, China) for seed weight. The measurements of each trait were made in triplicate with calculation of average and standard deviation.

### Evaluation of NDVI

To assess the condition of soybean cultivars according to the normalised difference vegetation index (NDVI) under two contrasting conditions (well-irrigated and drought), the GreenSeeker Handheld device (Trimble, Westminster, CO, USA) was used. The measurement was carried out according to the manufacturer’s instructions (GreenSeeker, 2023). Leaf diagnostics of all genotypes of the collection using a GreenSeeker Handheld optical sensor were carried out every 14 days from the soybean trifoliate leaf phase (V1) to the beginning of the maturing phase (R7). During measurement, the device assessed plants from the entire 5-m^2^ area of plots for each cultivar in three replicates. The measurements were carried out during the daytime (from 11:00 to 13:00). For each soybean genotype and replicate, average NDVI values for the growing season were derived.

### Drought sensitivity index

Drought sensitivity index (DSI) was calculated in plants of each soybean genotype using the formula (Fischer and Maurer, 1978):


DSI=(1–Y/Yp)/(1–X/Xp)

where *Y* is the yield of the genotype under stress conditions; *Yp* is the yield of the genotype without stress; *X* is the average yield for all varieties of one maturity group under stress; and *Xp* is the average yield for all cultivars in one maturity group without stress. Genotypes showing the lowest DSI values are considered as the most tolerant to drought.

### DNA extraction and 295K DArT analysis

Plants of all 188 studied soybean accessions and two hybrids (described in the separate section below) were grown in field conditions as mentioned above. The first trifoliate leaf was collected in each accession, hybrid parent, and breeding line from a single 18-day-old plant. Leaf samples were collected in 2-ml microtubes and transported to the laboratory in an insulated cooler with ice. DNA was extracted from fresh leaf samples of using the CTAB method as described earlier (Murray and Thompson, 1980).

Extracted DNA was purified using the GeneJET Plant Genomic DNA Purification Mini Kit (Thermo Fisher Scientific, USA). The DNA concentration was measured using a NanoDrop spectrophotometer (Thermo Fisher Scientific, USA), and integrity was checked by electrophoresis in a 1% agarose gel. The concentration of DNA was adjusted to 100 ng/µl, as required for DArT analysis. DNA samples were aliquoted into 50-µl volumes and submitted for genotyping using Soybean DArTseq (1.0) at Diversity Arrays Technology Pty Ltd (Canberra, Australia). This technology is based on sequencing of an enhanced library using a next-generation sequencing (NGS) platform with genome complexity reduction, and 295K DArT clones were applied for the study. However, five accessions did not pass quality control for DArT and were excluded from further analyses. Results were presented in two major files with the In-Silico-DArT and SNP-map used for further analysis (**Supplementary Material S3**).

### GWAS analysis with four models and QTN identification

To identify SNP with significant linkage to the studied yield-related soybean traits, a genome-wide association study (GWAS) was conducted using the Genome association – Prediction integrated tool (GAPIT) version 3 with several models with increased power and accuracy for genome association (Wang and Zhang, 2021). The GAPIT models used in this study include the Bayesian-information and Linkage-disequilibrium iteratively nested keyway (BLINK) (Huang et al., 2019), the Fixed and random model circulating probability uniform (FarmCPU) (Liu et al., 2016b), the Multiple loci mixed model (MLMM) (Segura et al., 2012), the Mixed linear model (MLM) (Zhang et al., 2010), and the General linear model (GLM) (Nelder and Wedderburn, 1972). To identify highly significant associations, in this study, rigorous logarithmic of odds (LOD ≥6.0) criteria were used, based on the Bonferroni correction test (α=0.05) (Bland and Altman, 1995).

The STRUCTURE v2.3.3 software was employed to assess population structures utilising a Bayesian–Markov chain–Monte Carlo (MCMC) method grounded in admixture and correlated allele frequencies (Evanno et al., 2005) The data set was run through 10,000 Markov chain–Monte Carlo iterations with an initial burn-in period of 10,000 with five replicates, considering several subgroups (K) ranging from 1 to 10. The python script of Structure Harvester “StructureHarvester.py” (Earl and con Holdt, 2012) was used to determine the optimal k-value (Structure Harvester, 2022), as well as to illustrate the results obtained from STRUCTURE v2.3.3.

### Molecular genetic phylogeny of soybean accessions using DArT analysis and dendrogram preparation

The molecular-phylogenetic dendrogram was constructed from the SNP-map “csv” Excel file with 295K DArT clones analysis. The results file was converted into a “nex” file for further use in SplitsTree4, version 4.14.4, BioNJ option tree style, from algorithms in the bioinformatics website at the University of Tübingen, Germany (SplitTree4, 2025).

### Location of QTN in chromosome genetic regions and candidate gene identification

Files with DArT data were arranged first in chromosome order and then linear order for all mapped DArT clones. The positions of DArT clones on chromosomes were checked for matches with reference soybean genome, cv. Williams 82, using the “JBrowse2 glyma.Wm82.gnm6” web page on the Legume Information System website (LIS, 2021). For candidate gene identification, at least two genes in the proximal and distal direction were found and assessed for suitability required for the best candidate gene. The information for potential high-confidence candidate genes was checked from various sources, including web sites and published papers, and considered for the final conclusion, which was verified using RT-qPCR analysis of gene expression.

### Hybrid breeding lines analysis from two crosses using 295K DArT

**Figure 1:**
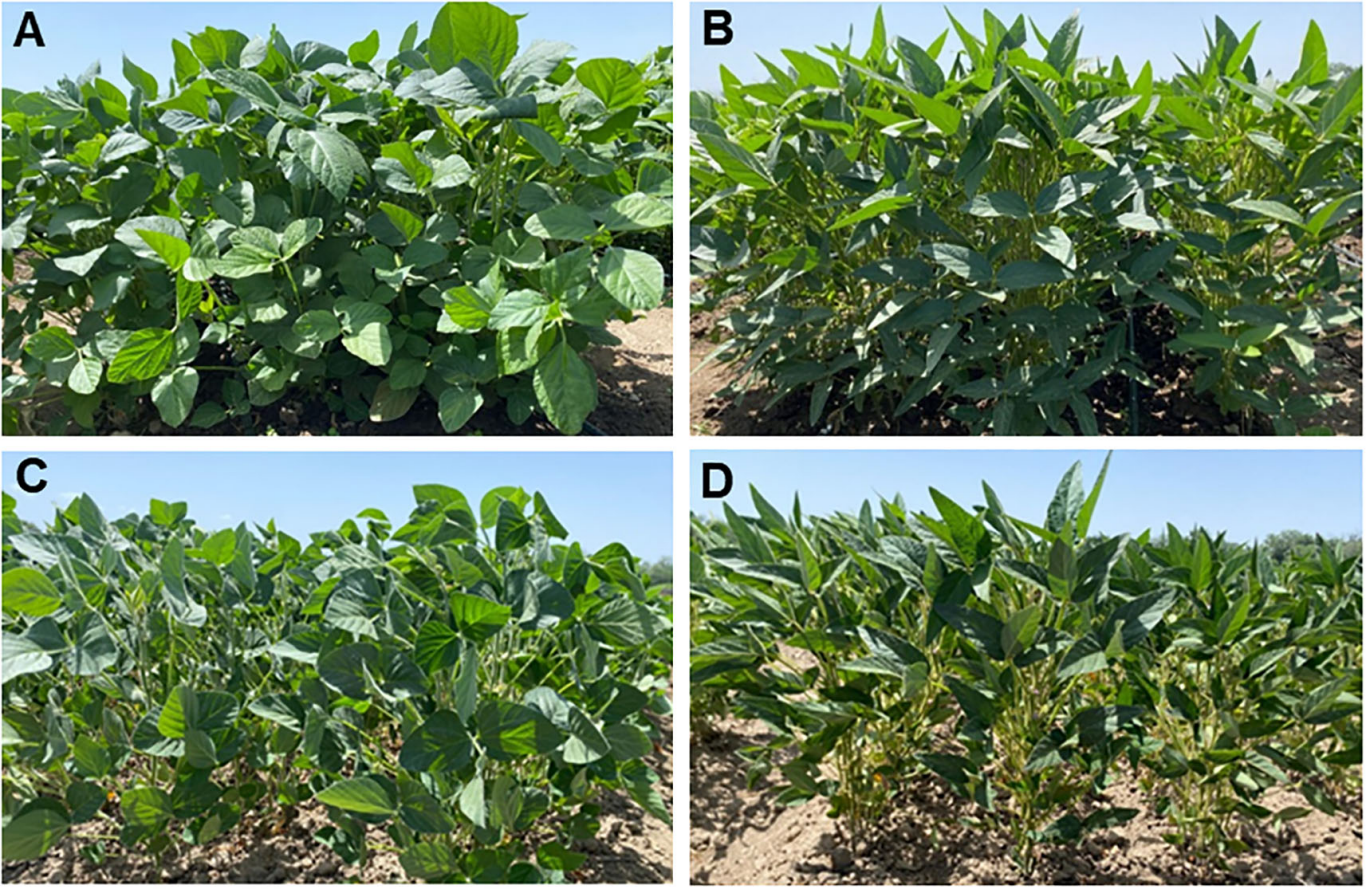
Plants of parent cultivars used for hybridisation, cv. Lastochka **(A)** and cv. Sponsor **(B)**, grown in well-watered conditions, and for comparison, the same cultivars under drought, Lastochka **(C)** and Sponsor **(D)**.

Two hybrid populations were developed between local and foreign soybean cultivars as follows: (LS1) ♀Lastochka × ♂Sponsor and (LZ2) ♀Lastochka × ♂Zen. Parents of the hybrids were highly contrasting in response to drought, where cultivars Sponsor and Zen showed higher seed yield and better drought tolerance compared with low-yielding plants of cv. Lastochka in drought conditions, based on previous studies (**Figure 1**). Three breeding lines, generations F_6_-F_7_, were selected from within each of the two hybrids and used in this study for mapping and segregation analyses.

For bulked segregant analysis (BSA), DNA was collected from 10 randomly selected individual plants and used both for bulking and individual genotyping. In the current study, 6 breeding lines (two hybrids and three breeding lines), 60 individuals, and 6 bulks in total were used together with their parents for genotyping with 295K DArT. This “modified” BSA analysis of hybrids with DArT markers was carried out to evaluate genetic polymorphism, levels of recombination, and heterozygosity both within and between breeding lines in each hybrid with the corresponding phenotyping for SWP in drought conditions.

### RNA extraction and RT-qPCR analysis of gene expression and role verification

Plants were grown in a greenhouse in containers (70 × 20 × 20 cm), seven plants per container of the same genotype, and six containers with a total of 42 plants. An identical set of plants was prepared and used for drought. Six soybean cultivars were selected: Vilana, Zen, Sponsor, Kye-shuan, Czi-ti-4, and Lactochka. The three first and three last cultivars represent high- and low-yielding under drought, respectively, based on previous tests. Additionally, these cultivars belong to maturity groups MG1, MG2, and MG3, respectively, among each of three first and last soybean cultivars. Seeds of these six cultivars were sown in the containers filled with 20 kg of soil collected from the nearby research field, irrigated with tap water two to three times per week using soil moisture sensors to monitor VWC as described above for field experiments, keeping the soil moisture level consistent at 80% field capacity. Plants were grown for 18 days after seed germination with 26°C/20°C day/night temperature, 50%-60% air humidity, and natural light during the spring season and in the same time-frame as the field research. After 18 days, when soybean plants had two trifoliate leaves, a single leaf was collected from each plant, three plants (biological replicates) in each genotype, and these samples were designated as time-point “0”. After sample collection, water was withheld in drought-treated plants, whereas control plants were watering as previously. Leaf samplings at time-points “1” and “2” were taken 7 and 14 days after time-point “0”, when mild and strong effects of dehydration and wilting leaves, respectively, were observed due to drought. Leaf samples were collected in 2-ml microtubes and frozen on dry ice with subsequent storage at −80°C.

RNA was extracted using TRIzol-like reagent following the protocol developed earlier (Shavrukov et al., 2013), and quantity and quality of extracted RNA samples were measured and assessed using a NanoDrop spectrophotometer (Thermo Fisher Scientific, USA) and electrophoresis on a 1% agarose gel. The cDNA was synthesised from 1 µg of each purified RNA sample using LunaScript RT SuperMix Kit (NEBiolab, USA) following the manufacturer’s protocol. 20 µl of synthesised cDNA samples was diluted with sterile water (1:5) and used for further RT-qPCR analysis. The reagent kit Power SYBR Green PCR Master Mix (Applied Biosystems, USA) was used in a 10-μl total reaction volume containing 0.5-μM primers and 3 μl of diluted cDNA and run in a QuantStudio 5 Real-Time qPCR system (Thermo Fisher Scientific, USA). Thermal cycling conditions consisted of an initial melt at 95°C for 3 min, followed by 40 cycles of 95°C, 5 s, and 60°C, 30 s, with a post-PCR melt curve from 60°C to 95°C increasing by 0.5°C increments every 5 s. Expression levels of target genes were normalised using the reference Actin-11 gene *GmAct11* (Glyma18g52780) (Wan et al., 2017). Specificities of target and reference gene amplifications were verified with single distinct peaks on a melting curve. The efficiencies of all qPCR products were calculated based on the slope of the corresponding calibration line, and their suitability was confirmed. The relative standard dilution method was used based on the ABI Guide for relative quantitation of gene expression using real-time quantitative PCR (ABI Guide, 2008), where serial dilutions were applied for each target and reference gene individually. Threshold cycle values were determined based on linear calibration of template cDNA dilution factor and Cq value. Sequences of all used primers with gene identification are present in **Supplementary Material S4**.

### Statistical treatment

Statistical data processing was carried out with R software, version 4.4.1, Race for Your Life (R, 2021), and the program JASP, version 0.19.3 (JASP, 2025). It included descriptive statistics for construction of distribution plots and boxplots. A one-way analysis of variance (ANOVA) was used for multiple comparison of environment, drought treatments, genotypes, and their interactions for yield and yield components. A similar one-way ANOVA with *post-hoc* Tukey HSD test was applied for multiple comparison of drought sensitivity index (DSI) in soybean accessions from different maturity groups as well as for analysis of gene expression (Statpage, 2025). Fisher *F*-criterion was carried out to estimate significant differences between the means of groups. For effect size measure, eta-squared (η²) was used providing a level of variability magnitude associated with group differences. Both *F*-criterion and η² were used for evaluation of environment, genotypes, and their interactions as factors determining variability of the studied traits (Fritz et al., 2012). The Pearson correlation coefficient (*r*) with linear regression criteria was determined between all variable measurement results (McDonald, 2014).

## Results

### Trait phenotypes and evaluation in fields

**Figure 2:**
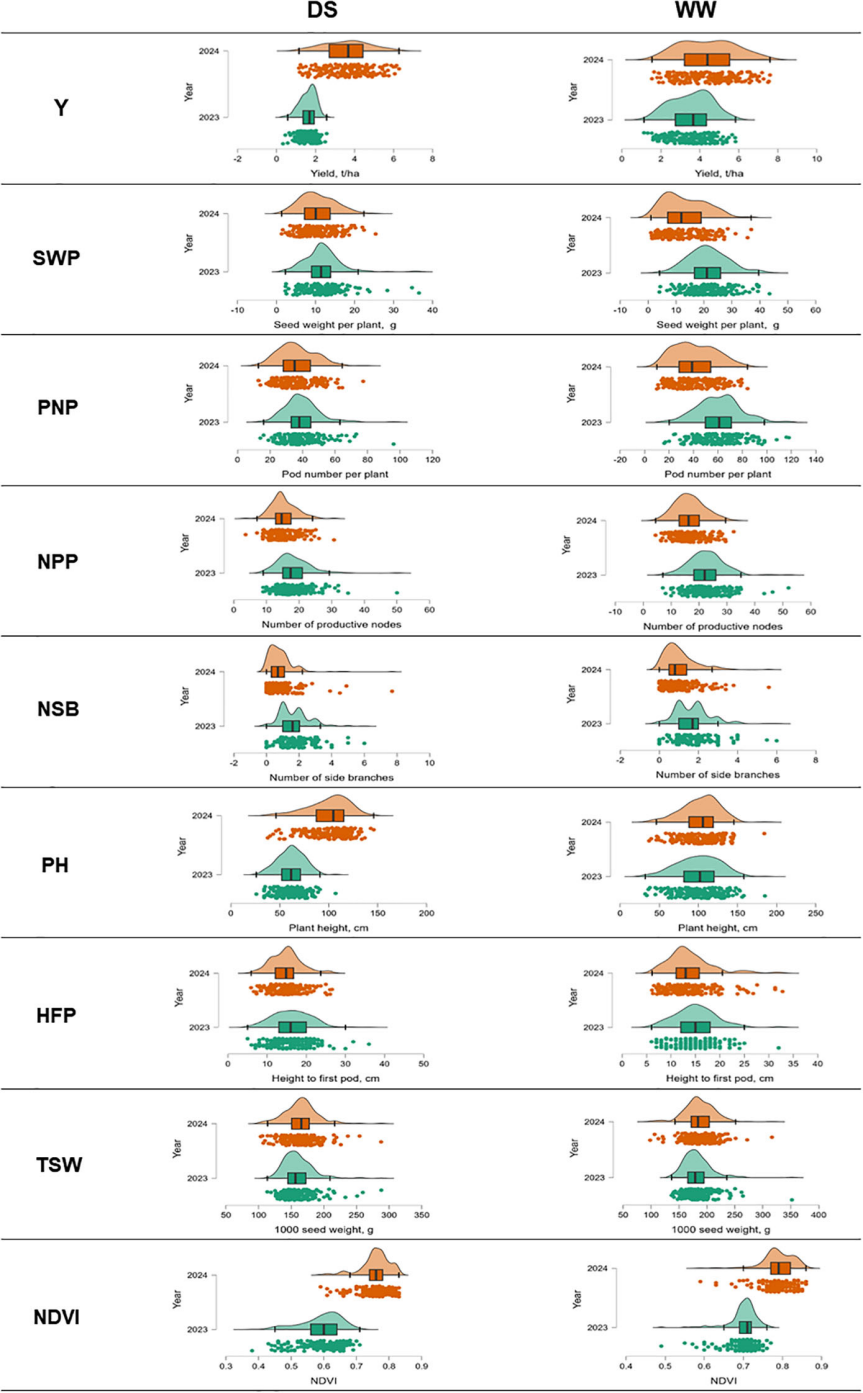
Distribution plots and boxplots showing data distribution for yield and yield components in 188 soybean accessions grown in two seasons and in field trials with and without irrigation in South-Eastern Kazakhstan.

In field trial tests, 188 soybean accessions were studied for yield (Y) and eight yield components: SWP, PNP, NPN, NSB, PH, HFP, TSW, and NDVI. The study was carried out in the environment of South-Eastern Kazakhstan, with and without irrigation. Yield and its components had extensive phenotypic variability in the two cultivation conditions and seasons (**Figure 2**; **Supplementary Material S5**).

**Figure 3:**
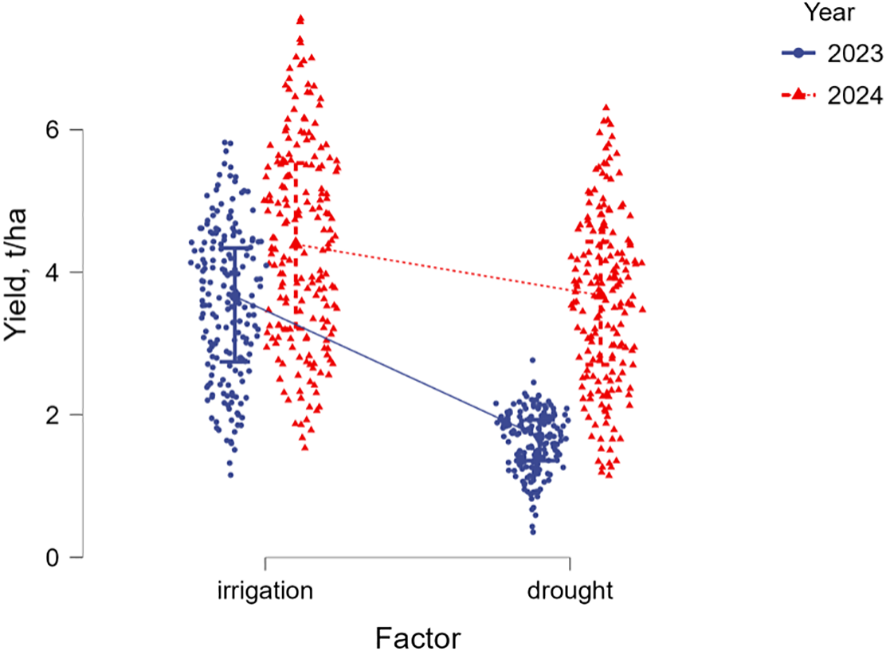
Yield data flex plot of 188 studied soybean accessions in irrigated and non-irrigated field trials in 2023 and 2024.

All 188 studied soybean accessions were represented by six maturity groups, from extra-early MG00 to very late maturity MG4. Therefore, yield was extremely diverse ranging from 0.33 to 6.28 t/ha without irrigation (drought) and from 1.13 to 7.6 t/ha with irrigation (WW). The average yield values for all maturity groups were 2.6 t/ha under drought and 3.98 t/ha in well-watered conditions (**Figure 2**; **Supplementary Material S5**). Under drought, the yield was reduced by 34.4% on average. Additionally, yield was found to be very different in the two studied years, 2023 and 2024, with very high level of probability (*F=*217.7; *p*<0.001). The conditions of 2023 were very dry, and, therefore, yield of soybean accessions was significantly lower in both irrigated and non-irrigated field trials compared with those in 2024 (**Figure 3**).

**Figure 4:**
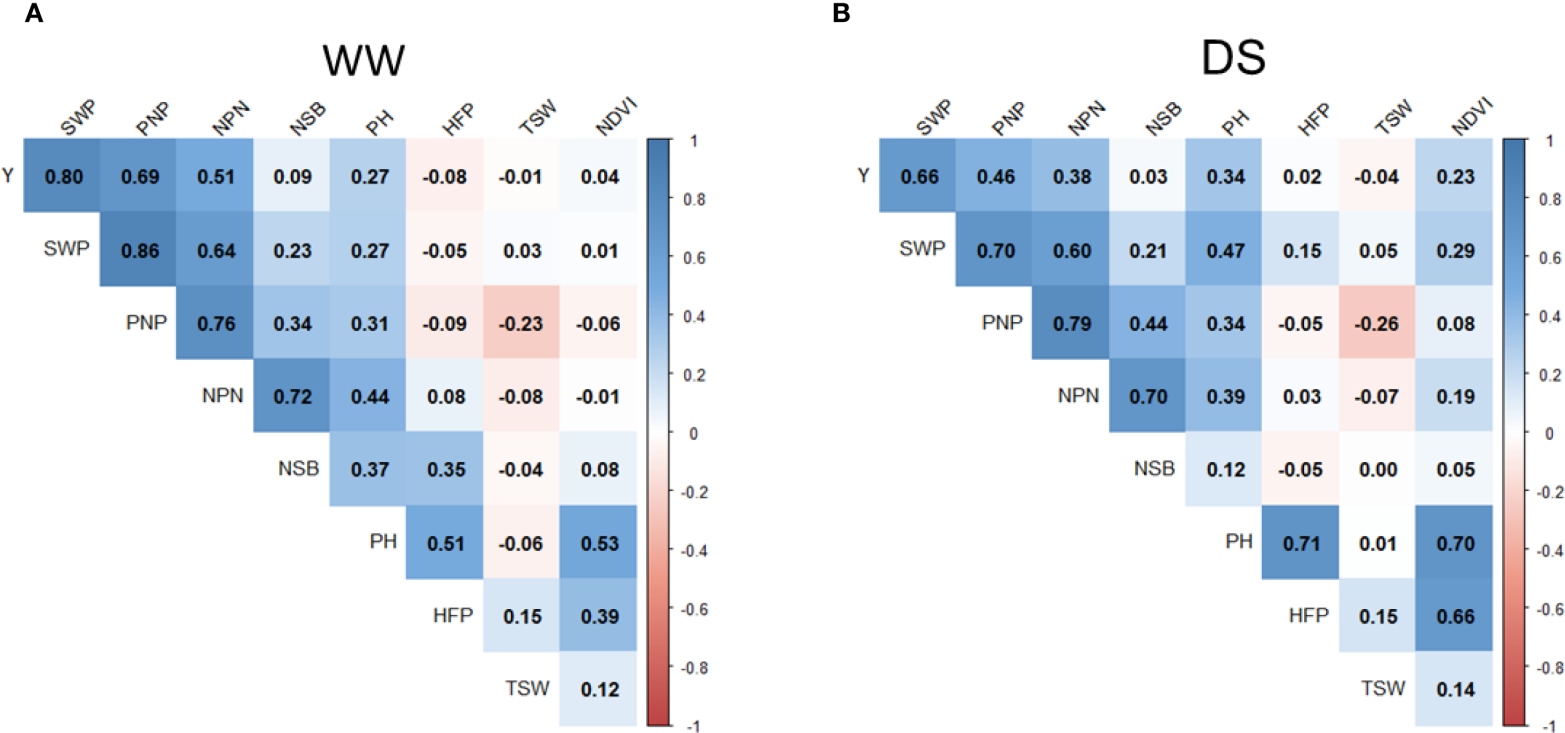
Pearson correlation analysis of yield and yield components in soybean accessions grown in two contrasting conditions. **(A)** WW (irrigated) and **(B)** drought (non-irrigated). Positive and negative correlations are shown by red and blue colours, respectively, whereas dark or light colour reflects correlation values ranging in the bars on the right hand-side in each figure panel. The studied traits were as follows: Y, yield of seeds per plot; SWP, seed weight per plant; PNP, pod number per plant; NPN, number of productive nodes; NSB, number of side branches; PH, plant height; HFP, height to first pod; TSW, thousand seed weight; NDVI, normalised difference vegetation index.

Seed weight per plant (SWP) is one of the important components of yield. In the current experiment with irrigation, the SWP trait demonstrated a significantly high correlation (*r*=0.80). A less but still significant correlation was found for SWP on seed yield under drought (*r*=0.66) (**Figure 4**). The average SWP value with irrigation was 17.36 g compared with 11.17 g under drought. Therefore, the impact of drought on GWP was estimated as a 35.7% reduction on average compared with those in WW field trials (**Supplementary Material S3**).

Pod number per plant (PNP) showed the next highest-ranking importance for a yield component. It can be clearly observed as a significant correlation between PNP and seed yield both in WW conditions (*r*=0.69) and moderate under drought (*r*=0.46). Even higher correlations were found between PNP and SWP, estimated as *r*=0.86 and *r*=0.70 in trials with and without irrigation, respectively (**Figure 4**).

The number of productive nodes (NPN) had a moderate effect for yield with *r*=0.51 and *r*=0.38 in WW и drought conditions, respectively, but there were very strong associations of NPN with PNP in WW and drought conditions with *r*=0.76 and *r*=0.79, respectively (**Figure 4**).

Other studied traits showed very little influence on seed yield, whereas a negative correlation was recorded for thousand seed weight (TSW). Plant height (PH) was associated with many other traits but together with height to first pod (HFP) and vegetation index NDVI, they did not affect seed yield (**Figure 4**).

In the current experiment, the role of separate factors as genotype, drought, and year as well as their “genotype × environment” interactions were analysed for yield and yield components in soybean accessions (**Table 2**). The interactions “genotype × drought” and “genotype × year” were absolutely much smaller than the three isolated factors “genotype,” “drought,” and “year” studied individually. Most of these interactions were not significant at all (**Table 2**).

**Table 2:** Analysis of the factors of drought, genotype, year, and their genotype–environmental interactions for soybean yield, yield components, and vegetation index NDVI using F, Fisher criterion, and η² estimated size effect.

Trait	Genotype		Drought		Year		Genotype × drought		Genotype × year	
	*F*	η²	*F*	η²	*F*	η²	*F*	η²	*F*	η²
Yield of seeds per plots (Y)	3.5***	0.33	429.8***	0.21	454.6***	0.23	0.54 ^na^	0.05	0.77 ^na^	0.13
Seed weight per plant (SWP)	2.5***	0.35	227.6***	0.17	132.6***	0.09	0.77 ^na^	0.11	0.84 ^na^	0.16
Pod number per plant (PNP)	3.08***	0.39	204.2***	0.14	153.4***	0.10	0.83 ^na^	0.11	0.74 ^na^	0.13
Number of productive nodes (NPN)	3.24***	0.44	81.8***	0.06	190.9***	0.14	0.68 ^na^	0.09	1.48***	0.18
Number of side branches (NSB)	3.68***	0.47	3.3***	0.002	220.6***	0.15	0.95 ^na^	0.12	1.18*	0.14
Plant height (PH)	6.5***	0.53	323.8***	0.14	302.3***	0.13	0.52 ^na^	0.04	0.19 ^na^	0.03
Height to first pod (HFP)	4.83***	0.62	22.94***	0.02	29.81***	0.02	0.63 ^na^	0.08	1,67***	0.16
Thousand seed weight (TSW)	5.6***	0.56	279.3***	0.15	12.4***	0.007	0.73 ^na^	0.07	0.88 ^na^	0.13
Normalised difference vegetation index (NDVI)	3.18***	0.18	478.2***	0.15	1630.2***	0.51	0.48 ^na^	0.03	0.37 ^na^	0.04

Significance levels are designated by asterisks (*, **, and ***), correspond to probability p<0.05, p<0.01, and p<0.001, respectively; ns, no significant differences.

Results of “genotype × environment” factors indicated that only isolated drought had very high and significant impact on Y (*F*=429.8, *p*<0.001), PH (*F*=323.8, *p*<0.001), and TSW (*F*=279.2, *p*<0.001). However, the highest influence of drought was found for NDVI, vegetation factor (*F*=478.2, *p*<0.001). The effect size measure in drought conditions was also quite significant, for example η²=0.21 for Y and η²=0.17 SWP and η²=0.15 for TSW.

The influence of genotype on yield and yield components was within the range of Fisher criterion *F* =2.5-6.5, with *p*<0.001. However, the highest role of genotypes was found for PH (*F*=6.5, *p*<0.001) and for TSW (*F*=5.6, *p*<0.001). The role of soybean genotype on studied traits in this experiment was high and varied from 0.33 to 0.62. The highest effect size measure for genotypes was recorded for HFP (η²=0.62), TSW (η²=0.56), and PH (η²=0.56).

The factor of year in the current study had a significant effect on yield and yield components. The highest effect was identified for Y (*F*=454.6, *p*<0.001), PH (*F*=302.3, p<0.001), and NSB (*F*=220.6, *p*<0.001). However, NDVI had the highest impact for year (*F*=1630.2, *p*<0.001). The effect size measure of year was also quite significant and estimated as η²=0.23 for Y, whereas the smallest effect of year was noted for the TSW (η²=0.007).

The drought sensitivity index (DSI) was used to assess the drought tolerance of soybean cultivars. In maturity groups MG00 and MG1, most cultivars with the highest yield under drought conditions also had the most favourable DSI values. However, there was a discrepancy in DSI ranking in maturity groups MG2 and MG3, i.e., cultivars with the best DSI values did not always show the highest yield in non-irrigated plots. At the same time, these cultivars were characterised by the smallest reduction of seed yield under drought conditions compared with well-watered controls.

**Figure 5:**
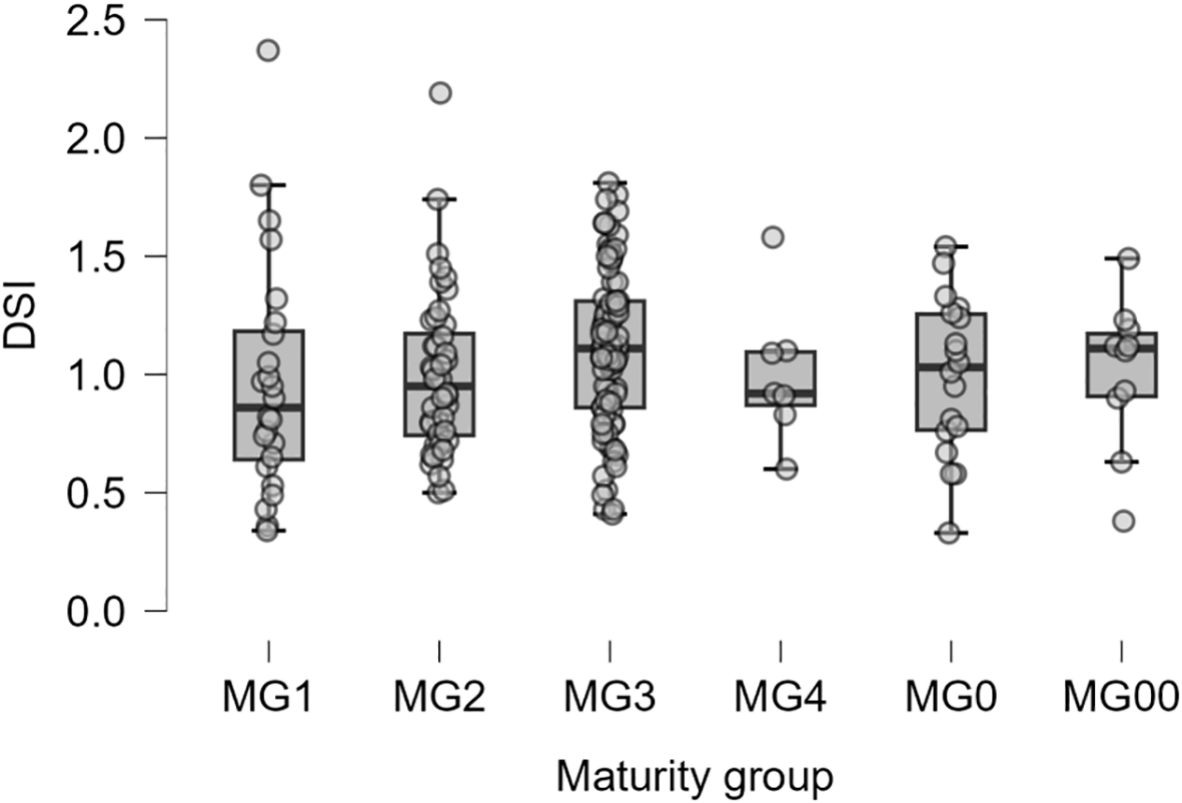
Boxplots showing the distribution of drought sensitivity index (DSI) among six maturity groups with 188 studied soybean accessions.

The correlation coefficient between DSI values and yield under drought conditions was negative (*r=*–0.11; *p*<0.02), but a lower DSI value means higher drought tolerance of the studied genotypes. The pairwise comparisons results using Tukey test for mean DSI values showed no significant differences between maturity groups (**Figure 5**).

### DArT analysis, population structure, and marker distribution

DArT-seq analysis was applied to study genetic polymorphism and variability among a set of 183 soybean accessions described above, with the exception of five accessions excluded from molecular analyses due to quality control. During an initial filtration of the 294,262 DArT markers “In Silico,” marker loci with unknown chromosome location, based on genome assembly, were removed from the analysis. The remaining DArT markers used for the association analysis were filtered out based on the following criteria: a call rate of ≤80%, marker reproducibility of ≤95%, minor allele frequency (MAF) ≤5%, and missing observation fractions ≥10%. Finally, after quality control, 16,063 DArT markers were retained for further investigation in the current study.

**Figure 6:**
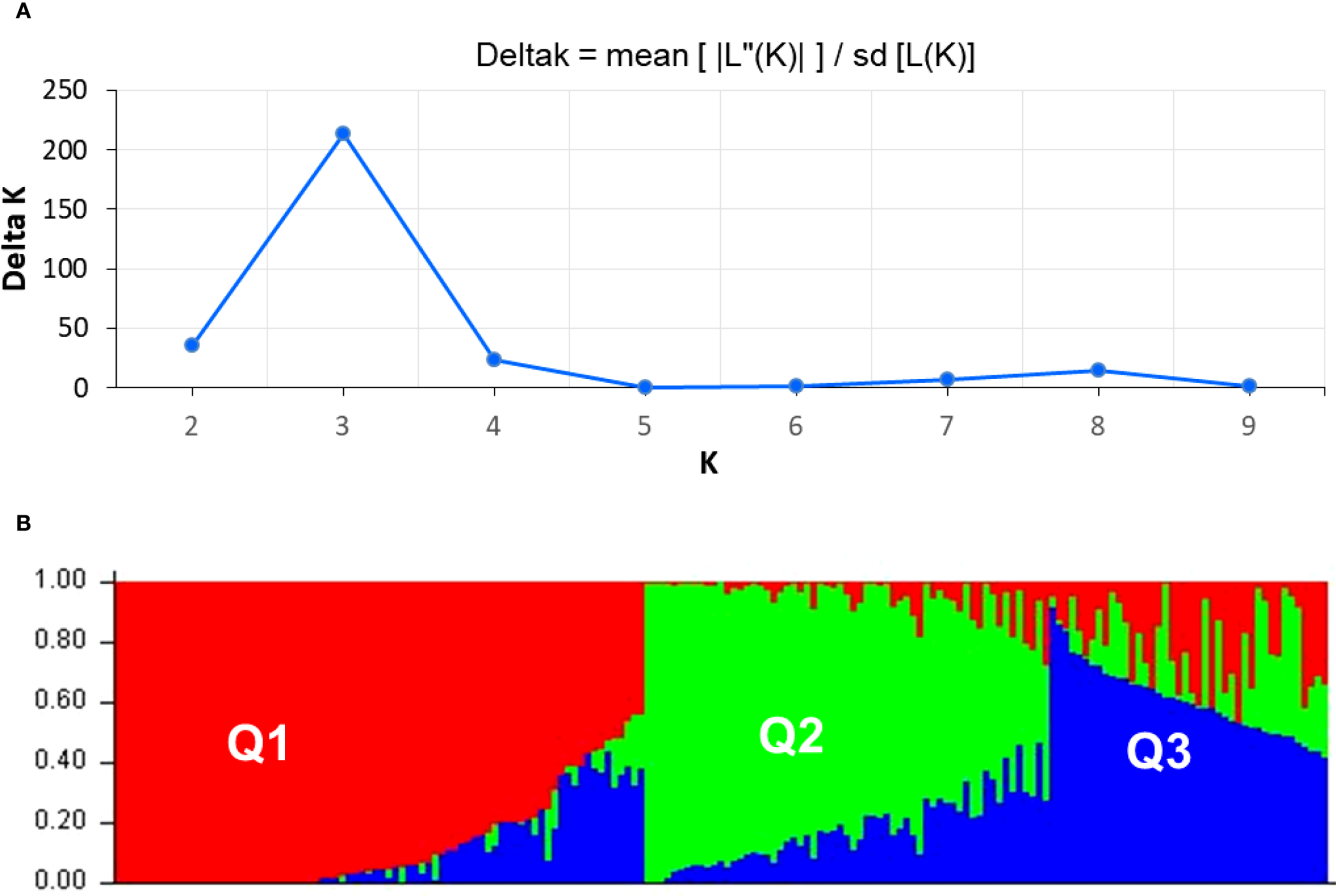
Population structure analysis of studied soybean accessions. **(A)** Delta K values for different numbers of populations assumed (K) in the STRUCTURE analysis. **(B)** Classification of soybean accessions into three “Q populations” (K=3) using STRUCTURE 2.3.3. The distribution of the accessions to different populations is indicated by the colour code. Numbers on the Y-axis show the subgroup membership, whereas the X-axis shows the distribution of 183 studied soybean accessions.

The genetic structure of 183 soybean accessions was assessed, employing 16,063 filtered DArT markers distributed across the genome. The dataset was evaluated using multilocus genotypic profiles to ascertain the optimal number of genetic clusters (K). The delta K plot indicated a peak at K=3, which was selected as the most likely number of subpopulations such as Q1, Q2, and Q3 (**Figure 6A**). In subpopulation Q1, 86 accessions were assigned, originating from Kazakhstan, Europe, East Asia, and North America. Among them, five accessions were considered as admixed according to the membership coefficient threshold. Most from 58 accessions in Q2, except six admixed ones, originated from Kazakhstan and Europe. Subpopulation Q3 included 39 accessions from Kazakhstan, Europe, and China, and admixture was classified in six accessions (**Figure 6B**).

### Molecular genetic phylogeny of studied soybean germplasm using DArT analysis

**Figure 7:**
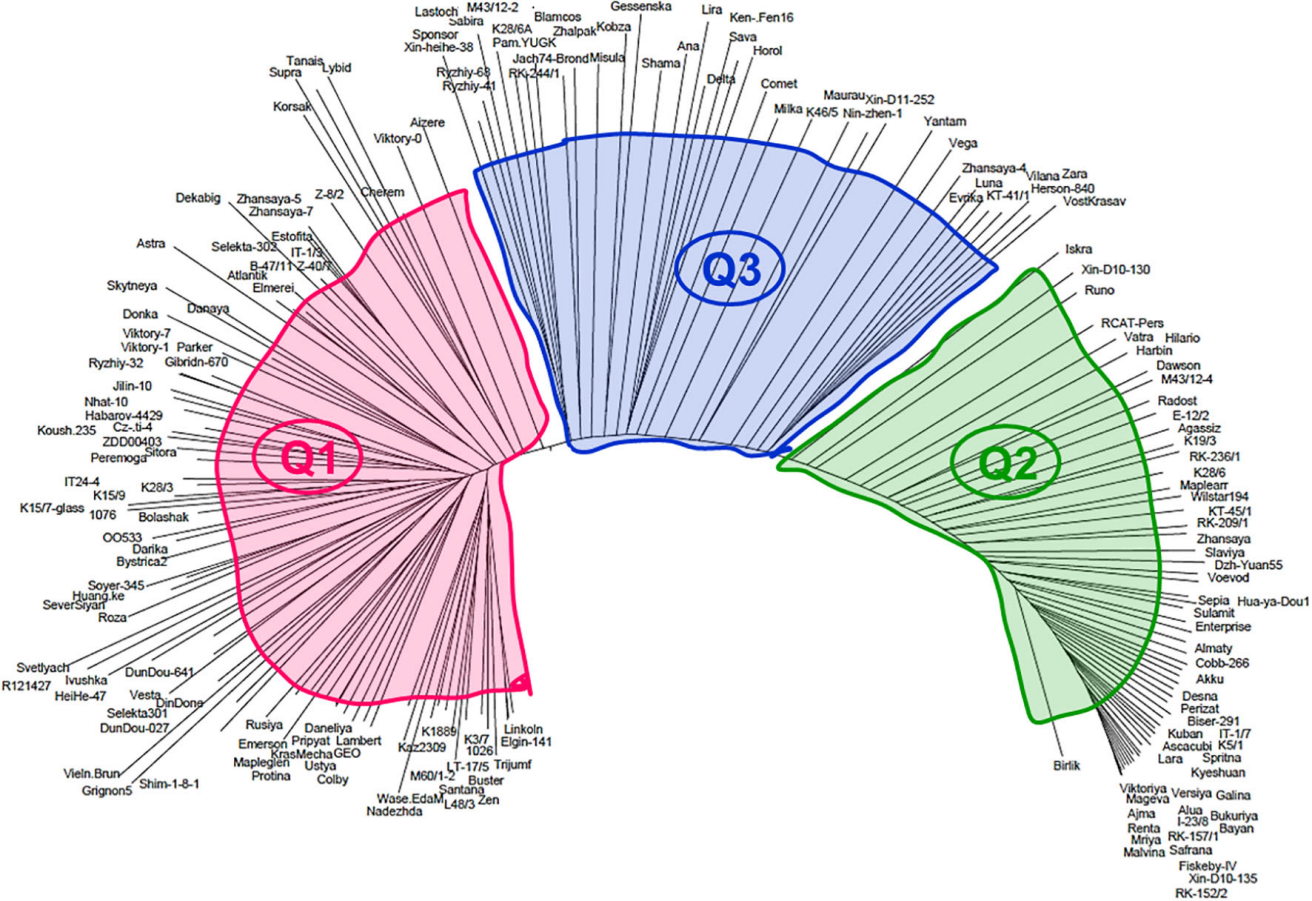
Distribution of the 183 soybean genotypes on a molecular dendrogram using 16K filtered DArT clones. Three Q-clades are indicated by colour and code. A full list of the accessions shown in clockwise order of their occurrence in each clade is presented in **Supplementary Material S1**.

To evaluate genetic diversity among the 183 studied soybean accessions, the molecular phylogenetic analysis based on 16K filtered DArT clones was carried out and the generated dendrogram is present in **Figure 7**. All studied genotypes were soybean cultivars and registered breeding lines and, therefore, their distribution among three big and crowded clades, identical to those in the population structure analysis above. All clades had accessions with mixed origin, which can reflect their pedigree history, and it is not always possible to identify for all studied soybean accessions.

Clade Q1 was present by 86 accessions from four major origins with the majority from Kazakhstan (29) approximately half of that with almost equal numbers from Europe (12), Canada–USA (11), Asia (17), and few others. In clade Q1, eight subclades were identified and present in **Supplementary Material S1** with some fluctuated frequency of the accession origin. Clade Q2 with 58 accessions is located in the distal part of the molecular dendrogram and very distanced from clade Q1. The proportion of geographic origin of soybean accession in clade Q2 remains approximately the same as in clade Q1. The number of soybean accessions from Kazakhstan (22) was approximately two to three times higher as from Europe (10), Canada–USA (8), Asia (6), and some other countries. Interestingly, clade 3 was located between first two clades sharing genetic polymorphism in a similar way with both distal parts of the presented phylogenetic tree. From 39 soybean accessions in clade Q3, 15 accessions originated from Kazakhstan, Europe, Canada–USA, and Asia had smaller portions with eight, two, and six accessions, respectively, and some more countries (**Figure 7**; **Supplementary Material S1**).

### GWAS with four models and identification of 41 QTN

The GWAS analysis included 10 traits study in 188 soybean accessions grown in two environments (WW and drought) over 2 years (2023 and 2024), and with genotyping of 183 studied germplasm using 16K filtered DArT markers. As indicated in the M&M section, five accessions did not pass quality control for DArT and were excluded from further analyses. These results revealed 41 identified QTN showing high significance (**Table 3**; **Supplementary Material S6**). Most identified QTN were found in drought stress studies, 24 in 2023 and 9 in 2024, whereas only 8 QTN were found in WW conditions, 4 in each of 2023 and 2024 (**Supplementary Material S6**).

**Table 3:** List of 41 identified QTN and relevant putative candidate genes in 183 soybean germplasm accessions based on GWAS analysis of marker–trait associations (MTA) between 16K-filtered DArT markers and nine major yield and yield-related traits in plants grown in two environments with regular and limited irrigation (well-watered and drought), during 2 years (2023 and 2024) in South-Eastern Kazakhstan.

QTN	DArT clone	Chr.	Putative candidate gene	Gene position on chromosome	Annotated description	References for gene description
Yield						
**QTN1-Yield**	**14967925**	**4**	**Glyma. 04G112700**	11,823,184-11,828,101	*Transducin-1* with WD40 repeats	Neer et al. (1994)
**QTN2-Yield**	**14970391**	**6**	**Glyma. 06G032500**	2,514,935-2,519,671	Glucose-6-phosphate isomerase (*G6PI=PGI*)	Gao et al. (2021a)
**QTN3-Yield**	**100499705**	**8**	**Glyma. 08G126200**	9,753,912-9,759,348	Rab-GDP dissociation inhibitor 2 (*Rab-GDI2*)	Bahk et al. (2009)
QTN4-Yield	14969881	18	Glyma 18G222500	53,626,652-53,641,652	Titan-9	Tzafrir et al. (2002)
QTN5-Yield	14982561	18	Glyma. 18G226500	54,205,371-54,275,961	NB-ARC domain disease resistance protein (*NB-ARC*)	van Ooijen et al. (2008)
Seeds weight per plant (SWP)						
QTN1-SWP	14982846	6	Glyma. 06G266500	47,545,898-47,552,336	ATP binding microtubule motor (*M.tubul-motor*)	Chen et al. (2024)
**QTN2-SWP**	**100499705**	**8**	**Glyma. 08G126200**	9,753,912-9,759,348	Rab-GDP dissociation inhibitor 2 (*Rab-GDI2*)	Hála et al. (2005)
QTN3-SWP	14977304	12	Glyma. 12G188100	38,435,948-38,440,478	Protein kinase (*PK*)	Dufayard et al. (2017)
QTN4-SWP	50677764	14	Glyma. 14G098900	9,748,407-9,757,546	F-box Leucine-rich repeat protein 15 (*F-box-LRR*)	Yu et al. (2020)
QTN5-SWP	14972694	14	Glyma. 14G112700	13,497,477-13,517,410	Histone-lysine N-methyl-transferase (*SUVR2*)	Liew et al. (2013)
**QTN6-SWP**	**14978704**	**15**	**Glyma. 15G092400**	7,099,029-7,120,507	ATP-binding cassette (*ABC*) transporter	Klein et al. (2006)
**QTN7-SWP**	**14981189**	**15**	**Glyma. 15G156600**	13,118,431-13,121,292	Pentatricopeptide repeat (*PPR*) protein	Chen et al. (2018)
QTN8-SWP	22920979	20	Glyma. 20G238500	50,140,311-50,143,656	Pre-mRNA-splicing factor	Rosenkranz et al. (2022)
Pod number per plant (PNP)						
QTN1-PNP	14981804	6	No any gene found	–	No any gene found	–
QTN2-PNP	50683668	9	Glyma. 09G208200	44,726,180-44,731,616	Cellulose synthase D3 (*CS-D3*)	Wu et al. (2023)
Number of productive nodes (NPN)						
**QTN1-NPN**	**14969732**	**5**	**Glyma. 05G163000**	37,634,948-37,639,388	Nitrate transporter (*NTR1.2*)	Nedelyaeva et al. (2024)
QTN2-NPN	100481948	17	Glyma. 17G248400	42,784,838-42,794,814	Metalloendopeptidase-zinc ion-binding protein	Ren et al. (2012)
Number of side branches (NSB)						
QTN1-NSB	14969678	3	Glyma. 03G041600	5,520,500-5,528,728	Protein SCAR2	Uhrig et al. (2007)
QTN2-NSB	14970470	4	Glyma. 04G028600	2,316,665-2,322,256	Beta-galactosidase 3 (*BG3*)	Yang et al. (2018)
Plant height (PH)						
QTN1-PH	14970055	7	Glyma. 07G000400	42,094- 58,689	Histone acetyltransferase *HAC1*	Liu et al. (2022)
**QTN2-PH**	**14978704**	**15**	**Glyma. 15G092400**	7,099,029-7,120,507	ATP-binding cassette (*ABC*) transporter	Wanke and Kolukisaoglu (2010)
QTN3-PH	14983750	16	Glyma. 16G176600	35,886,356-35,894,264	Receptor-like protein kinase 2 (*RLPK2*)	Liu et al. (2024)
**QTN4-PH**	**29305538**	**17**	**Glyma. 17G093400**	7,310,578-7,312,266	*Transducin-2* with WD40 repeats	Gachomo et al. (2014)
QTN5-PH	14975485	19	Glyma. 19G151300	44,257,603-44,259,910	Pentatricopeptide repeat (*PPR*) protein	van der Giezen et al. (2024)
Height to first pod (HFP)						
QTN1-HFP	14975791	4	Glyma. 04G055200	4,460,129-4,464,132	Mechanosensitive ion channel protein	Hamilton et al. (2015)
QTN2-HFP	14968153	4	Glyma. 04G082700	6,951,452-6,962,443	Peptide transporter 1 (*PT1*)	Wu et al. (2025)
QTN3-HFP	14972262	8	Glyma. 08G225302	18,361,851-18,366,217	P-loop containing nucleoside triphosphate hydrolases (*P-NTPH*)	Leipe et al. (2003)
QTN4-HFP	22920754	15	Glyma. 15G117100	9,160,663-9,166,841	Myb DNA-binding domain protein	Roy (2016)
QTN5-HFP	29305595	15	Glyma. 15G211600	34,305,648-34,308,370	PHD finger protein	Quan et al. (2023)
QTN6-HFP	14974197	15	Glyma. 15G233400	46,174,045-46,179,394	NB-ARC domain disease resistance protein (*NB-ARC*)	Pan et al. (2022)
QTN7-HFP	14969945	16	Glyma. 16G024900	2,405,982-2,414,133	Histone-lysine N-methyltransferase *ATX3*	Liu et al. (2022)
**QTN8-HFP**	**14969998**	**18**	**Glyma. 18G029000**	2,200,543-2,206,490	Auxin transporter (*AUXT1*)	Yang et al. (2021)
QTN9-HFP	14980986	19	Glyma. 19G251700	52,885,119-52,890,402	Essential nucleolar protein, small subunit processome	Broeck and Klinge (2022)
Thousand seeds weight (TSW)						
QTN1-TSW	14965896	6	Glyma. 06G041400	3,133,973-3,137,754	HXXXD-acyl-transferase	Sinka et al. (2024)
QTN2-TSW	14976250	16	Glyma. 16G146900	32,782,187-32,785,178	Phosphoglycerate mutase (*PGM*)	Duminil et al. (2021)
QTN3-TSW	24388245	17	Glyma. 17G239500	41,935,411-41,954,004	ATP binding microtubule motor (*M.tubul-motor*)	Jin et al. (2025)
Normalised difference vegetation index (NDVI)						
QTN1-NDVI	24386134	2	Glyma. 02G202500	44,951,358-44,955,905	Aldehyde dehydrogenase 2, member C4-like (*ADH-C4*)	Su et al. (2022)
QTN2-NDVI	14970403	4	Glyma. 04G032600	2,585,516-2,595,004	Glucose-6-phosphate isomerase (*G6PI=PGI*)	Grauvogel et al. (2007)
QTN3-NDVI	14972262	8	Glyma. 08G225302	18,361,851-18,366,217	P-loop containing nucleoside triphosphate hydrolase (*P-NTPH*)	Vaccaro and Andre (2022)
QTN4-NDVI	14983239	17	Glyma. 17G214100	38,291,147-38,294,163	Receptor-like protein kinase 4 (*RLPK4*)	Yang et al. (2022)
QTN5-NDVI	86239108	18	Glyma. 18G089500	8,897,657-8,903,847	Transcription initiation factor TFIID subunit (*TFIID*)	Cavel et al. (2011)

Relevant references were added describing functions of similar genes in various plant species. The QTN were detected using four methods of bioinformatics described in the M&M section. In the table, 10 significant QTN with eight corresponding putative candidate genes are indicated in bold. Additional information is provided in **Supplementary Material S6**.

In nine studied traits, QTN were distributed variably. They included five QTN in Y; eight QTN in SWP; two QTN in each of PNP, NPN, and NSP; five QTN in PH; nine QTN in HFP; three QTN in TSW; and five QTN in NDVI. The majority of QTN were unique, where each DArT marker showed a significant association only with a single trait in one condition. However, two markers were found to be associated with two traits. These include (1) DArT marker 100499705 in chromosome 08 associated with QTN3-Yield and QTN2-SWP and (2) DArT marker 14978704 in chromosome 15 associated with QTN6-SWP and QTN2-PH (**Table 3**; **Supplementary Material S6**).

**Figure 8:**
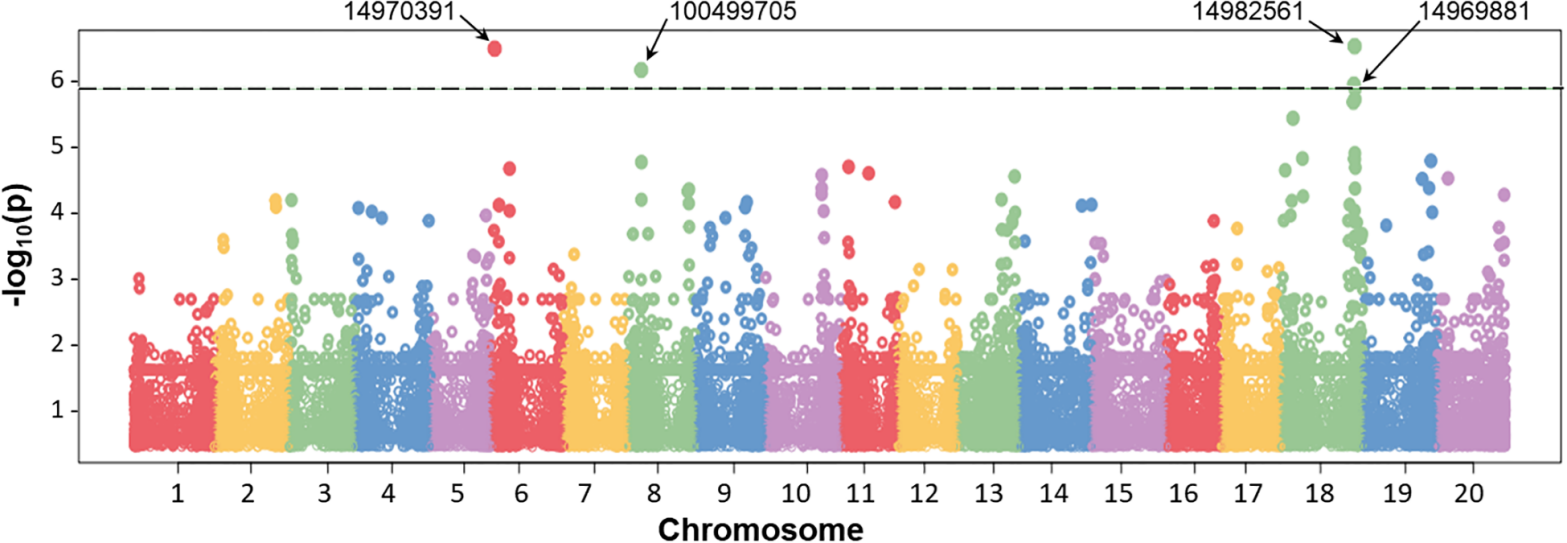
Example of a Manhattan plot of the association analysis in the water deficit experiment (drought) using the BLINK model. The positions on chromosomes are shown on the X-axis, whereas values of SNPs are shown on the Y-axis in the logarithmic scale. Dashed line indicates the threshold level of significance (−log_10_ *P*-value =  6.0). Four significant identified DArT markers are shown at the top by arrows, which were identified during seed yield (Y) analysis, and their IDs are presented in **Table 3**.

Genetic regions were identified for each of 41 QTN indicated above, based on DArT clones. An example of such DArT clones, which were identified and used for QTN analysis for the trait of seed yield (Y), is present in **Figure 8**. GWAS results and Q–Q plots for other traits are present in **Supplementary Material S7**. Assessment of at least two proximal and two distal genes from each DArT clone revealed names, ID, and annotated functions of the most suitable candidate genes. Only one case for “Pod number per plant” trait, QTN1-PNP, with DArT clone 14981804, on chromosome 6, did not yield any suitable putative candidate gene with an extremely large fragment of chromosome without any genes nearby in this DArT clone. In all other 40 QTN, candidate genes were identified and accompanied at least by one reference for relevant published papers indicating their biological roles in plants, presented in **Table 3**.

### Hybrid breeding lines analysis from two crosses with modified BSA using 16K filtered DArT

In addition to GWAS, segregation analysis in breeding lines was carried out in two hybrids, LS1, Lastochka × Sponsor, and LZ2, Lastochka × Zen (**Table 4**). From three studied breeding lines in two hybrid populations, one line LS122 in hybrid LS1 and another line LZ52 in hybrid LZ2 were closely similar to the paternal parents, Sensor and Zen, respectively. These lines showed 2-3-fold higher SWP under drought conditions compared with two other lines in the same hybrids, which were similar to the maternal parent, cv. Lastochka.

**Table 4:** Comparative analysis of genetic polymorphism in chromosome regions with 45 identified QTNs using 16K filtered DArT markers in breeding lines with BSA and individual plants, 10 plants in each line, from two hybrids with contrasting phenotypes.

Breeding lines	SWP, g	41 QTN in 10 plants				Differential QTN identified in the studied breeding lines
		All monomorphic	All identical to ♀	All identical to ♂	Segregated	
Hybrid LS1: [♀Lastochka × ♂Sponsor]						
Line-LS25	16.0	22	6	9	4	(1) QTN1-Yield: Glyma.04G112700=*Transducin-1*; (2) QTN3-Yield and (3) QTN2-SWP: Glyma.08G126200=*Rab-GDI2*; (4) QTN7-SWP: Glyma.15G156600=*PPR*; (5) QTN1-NPN: Glyma.05G163000=*NTR1.2*; (6) QTN4-PH: Glyma.17G093400=*Transducin-2*;
Line-LS91	11.8	22	6	11	2	
**Line-LS122**	**28.8**	22	1	16	2	
Hybrid LZ2: [♀Lastochka × ♂Zen]						
**Line-LZ52**	**35.3**	22	3	15	1	(1) QTN2-Yield: Glyma.06G032500=*G6PI* (*PGI*) (2) QTN6-SWP and (3) QTN2-PH: Glyma.15G092400=*ABC* transporter: (4) QTN7-SWP: Glyma.15G156600=*PPR*; (5) QTN8-HFP: Glyma.18G029000=*AUXT1*;
Line-LZ163	14.3	22	10	7	2	
Line-LZ213	10.1	22	9	7	3	

High-yielding breeding lines are indicated in Bold.

The BSA and study of individual plants in six breeding lines were applied to study 41 identified QTN in GWAS using the same 16K filtered DArT assay. It is important to note in the beginning that only one DArT clone was targeted in each studied QTN, but it represents a haplotype inherited as a genetic fragment (sometimes, significantly long) without recombination events. This was based on genotyping of other closely located DArT clones nearby, identical to the same parent as in the targeted DArT clone.

The BSA results of the current study, presented in **Table 4**, indicated that more than half of 41 QTN were monomorphic among parents and bulked breeding lines, which can reflect the limitation of biparental hybrid analyses compared with GWAS. The rest of 19 studied QTN among 10 individual plants in each breeding line were distributed in three groups: segregated or non-segregated with genotypes identical to maternal or paternal parent. This research fragment was carried out on individual plants.

In hybrid LS1, the number of QTN identical to drought tolerant parent cv. Sponsor was highest in 10 individual plants and in each of the breeding lines 9, 11, and 16, respectively. However, the number of QTN identical to the maternal parent, drought sensitive cv. Lastochka, was critical to identify differences between breeding lines, accounting 6, 6, and 1 QTN, respectively (**Table 4**). More spectacular differences in QTN were found in hybrid LZ2. The numbers of the “maternal type” of QTN in breeding lines were 3, 10, and 9, whereas the numbers of “paternal-type” QTN were 15, 7, and 7 in the same breeding lines (**Table 4**).

Finally, six QTN were identified for differences between breeding lines in hybrid LS1 for QTN1-Y, QTN3-Y, QNT2-SWP, QTN7-SWP, QTN1-NPN, and QTN4-PH. In breeding lines of hybrid LZ2, five differential QTN were found, namely, QTN2-Y, QTN6-SWP, QTN2-PH, QTN7-SWP, and QTN8-HFP (**Table 4**).

### Selection of significant QTN: candidate gene identification for QTN and their annotation and relevance to the study

Based on combined analysis of GWAS and BSA, 10 significant QTN and 8 corresponding putative candidate genes were identified as having important potential functions in plant growth under drought and were used for more detailed analysis and verification of their roles *via* gene expression analysis using RT-qPCR. These genes showed association with several yield and yield-related traits as follows: Y, SWP, PH, NPN, and HFP, and their descriptions are present in **Table 5**. Some genes are enhanced and upregulated whereas other genes are suppressors and were downregulated.

**Table 5:** Eight selected putative candidate genes in soybean for RT-qPCR analysis in dehydration and control (well-watered) conditions.

Putative candidate gene	Chr	Position on chromosome	Annotated description	QTN
Glyma.08G126200	8	9,753,912-9,759,348	Rab-GDP dissociation inhibitor 2 (*Rab-GDI2*)	QTN3-Yield
				QTN2-SWP
Glyma.06G032500	6	2,514,935-2,519,671	Glucose-6-phosphate isomerase (*G6PI=PGI*)	QTN2-Yield
Glyma.04G112700	4	11,823,184-11,828,101	*Transducin-1* with WD40 repeats	QTN1-Yield
Glyma.17G093400	17	7,310,578-7,312,266	*Transducin-2* with WD40 repeats	QTN4-PH
Glyma.15G156600	15	13,118,431-13,121,292	Pentatricopeptide repeat (*PPR*) protein	QTN7-SWP
Glyma.15G092400	15	7,099,029-7,120,507	ATP-binding cassette (*ABC*) transporter	QTN6-SWP
				QTN2-PH
Glyma.05G163000	5	37,634,948-37,639,388	Nitrate transporter (*NTR1.2*)	QTN1-NPN
Glyma.18G029000	18	2,200,543-2,206,490	Auxin transporter (*AUXT1*)	QTN8-HFP

### RT-qPCR expression analysis of eight selected genes in response to drought

**Figure 9:**
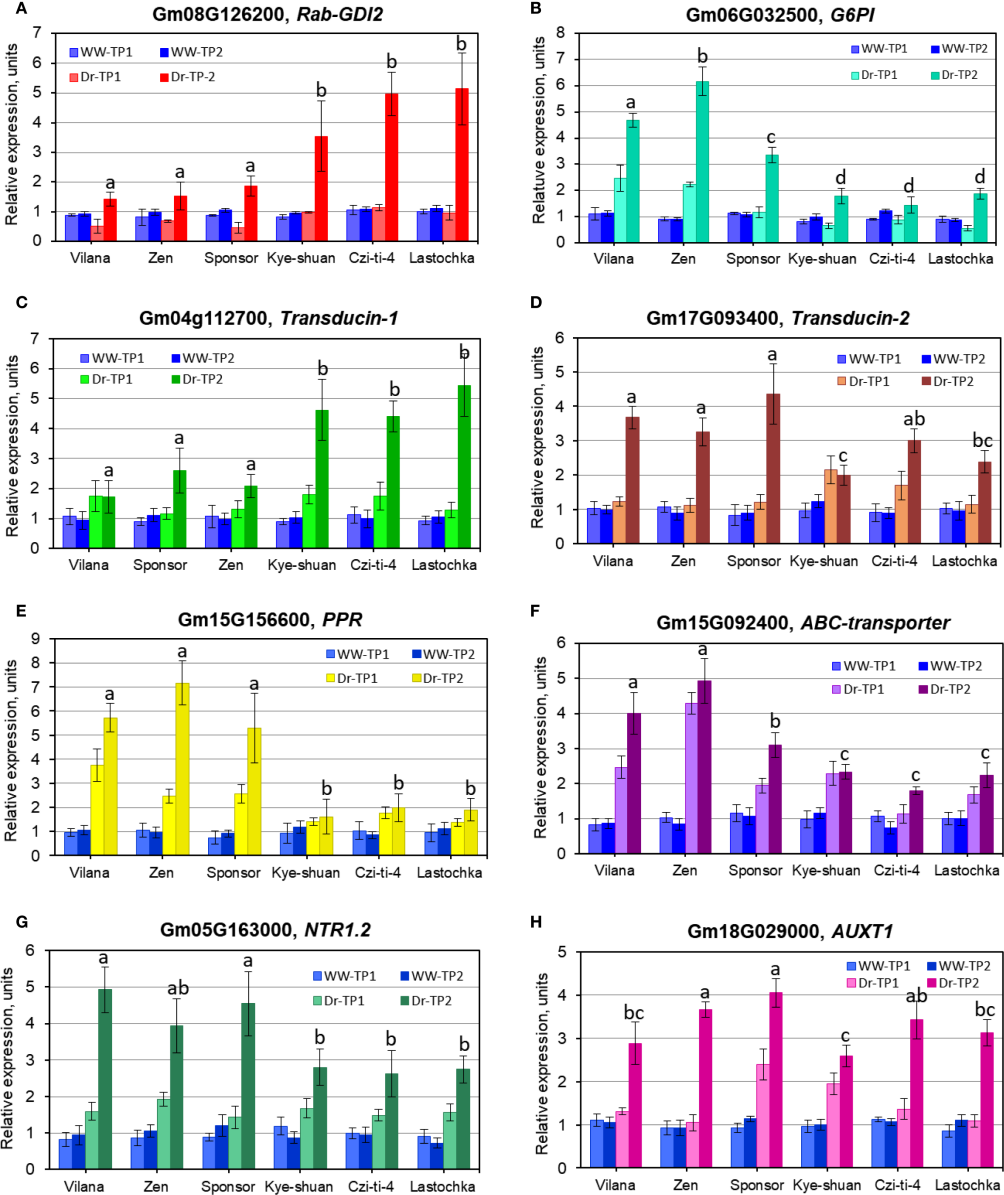
RT-qPCR expression analysis of eight selected genes in six soybean cultivars: **(A)** Glyma.08G126200, GmRabGDI2; **(B)** Glyma.06G032500, G6PI; **(C)** Glyma.04G112700 and **(D)** Glyma.17G093400, for Transducin-1 and -2, respectively; **(E)** Glyma.15G156600, PPR; **(F)** Glyma.15G092400, ABC transporter; **(G)** Glyma.05G163000, NTR1.2; **(H)** Glyma.18G029000. AUXT1. The gene expressions at time-point 0 at the start of withholding watering, was set up as level “1” in each figure panel. Leaf samples collected at time-points 1 and 2 (7 and 14 days after time-point 0) are designated as TP1 and TP2, respectively. Well-watered controls (WW) are shown in blue, whereas drought treatments (Dr) are shown in different colours for each gene with the identification provided on the top of each figure panel. Expression data were normalised using reference gene *GmAct11* (Glyma18g52780), and these data are present as the average ± SE of three biological replicates (individual plants) for each genotype, time-point, and treatment. For simplicity of presentation, significant differences (*p*<0.05) between genotypes are shown by different letters only for time-point Dr-TP2 in each figure panel. Results for statistical treatment of all time-points calculated using one-way ANOVA with *post-hoc* Tukey-HSD test are present in **Supplementary Material S8**.

Expression analysis of eight putative candidate genes, indicated in **Table 5**, was carried out in six selected soybean cultivars. The first three cultivars, Vilana, Zen, and Sponsor, from maturity groups MG1, MG2, and MG3, respectively, were identified as high yielding in drought conditions and drought tolerant. The last three cultivars, Kye-shuan, Czi-ti-4, and Lastochka, with the same MG group order, were identified as low yielding in non-irrigated dry conditions and sensitive to drought (**Figure 9**). For simplicity of presentation, significant differences between genotypes are shown only for time-point Dr-TP2 for each candidate gene. Additionally, results for statistical treatment of all time-points are present in **Supplementary Material S8**.

The first *GmRab-GDI2* gene (Glyma.08G126200) showed no or very low expression in three drought tolerant cultivars (Vilana, Zen, and Sponsor), but much higher upregulation in three drought sensitive cultivars (Kye-shuan, Czi-ti-4, and Lastochka) (**Figure 9A**). A similar pattern was found in the *Transducin-1* gene with WD40 repeats, Glyma.04G112700 (**Figure 9C**).

In contrast, significantly higher mRNA synthesis was found in three drought tolerant cultivars (Vilana, Zen, and Sponsor) compared with three drought sensitive cultivars (Kye-shuan, Czi-ti-4, and Lastochka) in three genes, Glyma.06G032500, glucose-6-phosphate isomerase (*G6PI*=*PGI*) (**Figure 9B**); Glyma.15G156600, pentatricopeptide repeat (*PPR*) protein (**Figure 9E**); and Glyma.15G092400, *ABC transporter* (**Figure 9F**). Two other genes, Glyma.17G093400, the *Transducin-2* gene (**Figure 9D**) and Glyma.05G163000, Nitrate transporter, *NTR1.2* (**Figure 9G**), showed a similar tendency for gene expression but with some results overlapping between genotypes tolerant and sensitive to drought. Very different results were found for the last gene Glyma.18G029000. Auxin transporter *AUXT1* had mixed expression levels between drought tolerant and sensitive genotypes (**Figure 9H**).

Based on the presented results, it can be concluded that the first two described genes, *GmRab-GDI2* (Glyma.08G126200) and *Transducin-1* (Glyma.04G112700), are acting as the repressors, negatively regulating gene expression. In contrast, five other studied genes, *G6PI=PGI* (Glyma.06G032500), *Transducin-2* (Glyma.17G093400), *PPR* (Glyma.15G156600), *ABC transporter* (Glyma.15G092400), and *NTR1.2* (Glyma.05G163000), showed high expression (in various levels) in drought tolerant cultivars (Vilana, Zen and Sponsor) and confirmed their positive regulation for plant response to drought and dehydration. The expression of the last gene *AUXT1* has a very different pattern and did not directly relate to drought tolerant and sensitive cultivars used in the study.

## Discussion

Seed yield is the final step after harvesting of most crops including soybean, and it is determined by many seed-related traits. Any of these traits can be affected by drought but in a different range. Therefore, reduction of seed yield as an integrative trait is unavoidable in dry conditions. However, knowing which yield-related traits could be the most persistent and stable in support of yield under drought always remains a key issue of plant development with improved tolerance to conditions with insufficient water availability.

In the current study, yield and eight yield-related traits were studied in 188 soybean accessions, and the strongest association with seed yield was found in SWP in both WW and DS conditions (**Figure 4**). This result is very similar to what was previously published on 148 Chinese soybean cultivars, where 10 QTL were identified for SWP under drought and eight in WW conditions (Liu et al., 2023). In another study, two seed-related traits, SWP and PNP, with four QTLs were reported as most important for drought tolerance in both field and pot experiments with 188 soybean accessions, including cultivars and landraces, mostly from China and also from other countries (Li et al., 2023). The consensus results were present in the current study, where PNP was identified as the second biggest “contributor” to seed yield of soybean plants grown in both WW and DS conditions (**Figure 4**). Another report for GWAS with 585 soybean accessions in irrigated and non-irrigated field trials revealed that the drought tolerance index was determined based on yield and PH and resulted in QTN located in chromosome 8 (Zhang et al., 2024), which is also very similar to our results for QTN identification for Y and PH, especially for drought, presented in the current study (**Figure 4**).

In contrast, many other published reports with both cultivated and wild soybean plants with drought and dehydration were carried out either in earlier stages, for example in soybean young seedlings for survival rate and growth traits (Zhang et al., 2022) and for leaf slow wilting (Li et al., 2024; Nguyen et al., 2024), or in the germination stage (Liu et al., 2020; Aleem et al., 2024; Jia et al., 2024). Therefore, it is impossible to make a comparison with mature plants and yield-related traits in the current study. Additionally, dehydration experiments with PEG in controlled conditions cannot accurately simulate drought in soil and particular in field conditions, as demonstrated earlier (Kylyshbayeva et al., 2025).

In the current study, a slight but negative correction was found between TSW and seed yield particularly in drought conditions (**Figure 4**). TSW represents one of the very important traits characterising each soybean cultivar. Genotypes with high TSW do not always produce a bigger seed yield, showing a small to moderate level of positive correlation. Negative correlations between TSW and Y are also not surprising for plants grown in conditions where many pods are formed and there is a strong competition for nutrients. Therefore, depending on soybean genotype, an “alternative strategy” can be followed by plants, especially when grown in drought conditions: to produce either more seeds with smaller TSW or less seeds with higher TSW. This statement is supported by published reports (Xu et al., 2022; Jiang et al., 2024).

Based on DArT molecular-phylogenetic analysis, 183 soybean accessions used for GWAS were distributed in three Q-clades with almost similar proportions of their geographic origin. This can indicate that many of the studied soybean accessions, including the majority of local cultivars from Kazakhstan, shared a similar pedigree, and no particular isolation of any geographic group or country was found in any parts of the identified clades (**Figure 7**).

GWAS analysis helps to identify molecular markers, QTL or QTN, genetic regions on chromosomes and the most suitable candidate genes. In the current study, 41 QTN were identified for yield and eight yield-related traits in 183 soybean accessions based on 16K filtered DArT markers (**Table 3**). The set of soybean germplasms used and, most importantly, conditions of field trials carried out in Kazakhstan were very specific and perhaps very different from other reports published earlier (Li et al., 2023; Liu et al., 2023; Zhang et al., 2024). For these reasons, there were no QTN and putative candidate genes found overlapping with other studies. This fact indicates for the complexity of soybean plant responses to drought compared with WW conditions, where many genes are strongly involved. Nevertheless, the 41 identified QTN in the current study represent an important step for further evaluation of the presented data for development of molecular markers and MAS with the final target of producing novel drought tolerant soybean genotypes (**Table 3**).

However, 10 highly significant QTN and eight corresponding genes were selected for further analyses. For integrated seed yield trait, a putative candidate gene Glyma.08G126200, *Rab*-GDP dissociation inhibitor 2 or *Rab-GDI2*, was identified sharing two QTN3-Yield and QTN2-SWP, indicating that seed weight per plant remains the priority among the seed-related traits (**Tables 3** and **5**). This gene Glyma.08G126200 was found and confirmed during BSA of breeding lines from hybrid LS1, Lastochka × Sponsor (**Table 4**). *Rab-GDI* is a very well-known gene, part of the negative regulating network system together with *Rab-GTP*, small GTP-binding proteins (guanosine triphosphatases) (Khassanova et al., 2019), for intercellular vesicle trafficking of different molecules and compounds in plant cells (Takai et al., 2001; Bahk et al., 2009). It was shown for different plant species including grape, *Vitis vinifera* L. (Abbal and Tesniere, 2010), and drumstick tree, *Moringa oleifera* Lam. (Jabeen et al., 2022). *Rab*-GDI was reportedly highly expressed in roots of *Medicago truncatula* L. (Yaneva and Niehaus, 2005). but sharply downregulated in PEG-induced dehydration in leaves of mango tree, *Mangifera indica* L. (Liu et al., 2015). This gene was expressed in leaves and roots of *Solanum chilense* Dunal. under salinity stress (Martín-Davison et al., 2017) and highly overexpressed with *AtGDI* from *Arabidopsis thaliana* in yeast (Ueda et al., 2000). In the current study, the *GmRab-GDI2* gene (Glyma.08G126200) showed no or very low expression in three drought tolerant cultivars but much higher upregulation in soybean cultivars sensitive to drought treatment (**Figure 9A**). This can indicate that dissociation factor Rab-GDI2 is strongly involved in drought tolerance and acts as a negative regulator in soybean plants under drought.

Glyma.06G032500 represents another putative candidate gene, *G6PI*, glucose-6-phosphate isomerase, also known by the synonym *PGI*, phosphoglucose isomerase, and, therefore, both names are used in our study, *G6PI=PGI* (**Tables 3** and **5**). In the BSA study of hybrid LZ2, Lastochka × Zen, the *PGI* gene was found to be involved in the genotype differences among parents and breeding lines (**Table 4**). This PGI enzyme has shown both strong conservatism and several global rearrangements during evolution as reflected in the diversity between land plants and algae (Grauvogel et al., 2007). In *Arabidopsis*, PGI1 was an important protein involved in seed yield with GA-mediated development of reproductive organs and the metabolic storage of G6P in the embryos (Bahaji et al., 2018). Overexpression of wheat *TaPGIc* in chloroplasts of the *atpgip* mutant of *Arabidopsis thaliana* resulted in significantly higher plant biomass and seed yield (Gao et al., 2021a). In the current study, our results for RT-qPCR analyses confirmed a high level of mRNA synthesis in response to drought in high-yielding and drought tolerant soybean accessions (**Figure 9B**).

The following QTN1-Yield was associated with candidate gene Glyma.04G112700, encoding Transducin protein with WD40-repeats, whereas the additional homolog of this gene, Glyma.17G093400, was also identified in QTN4-PH for plant height (**Tables 3** and **5**). Both *Transducin* genes, Glyma.04G112700 and Glyma.17G093400, were identified in BSA of breeding lines from hybrid LS1, Lastochka × Sponsor (**Table 4**). Transducin with WD40 repeats represents a very big family with conservative repeats ending with amino acids Trp-Asp (WD) and was firstly found in GTP-binding proteins, a partner gene indicated above, which transduce or transfer a signal through the cell membrane (Neer et al., 1994). These genes were reported as involved in multiple aspects of chromatin assembly and dynamics in plant cells, and they also negatively regulated networking genes. In rice (*Oryza sativa* L.), expression profile analysis of 200 *OsWD40* genes showed very diverse up- and downregulations in different tissues and stages of plant development with proposed interactions with other genes involved in signalling and metabolic pathways (Ouyang et al., 2012). In *Arabidopsis thaliana*, WD-repeat genes were reported to be involved in plant development, meristem structure, flowering, and floral development (van Nocker and Ludwig, 2003). Two *Arabidopsis* genes, *MSI1* and *GTS1* (Gigantus1), with WD repeats were described as negative regulators of drought-inducible target genes *via* chromatin binding. The *MSI1* gene was involved in plant response to drought stress (Alexandre et al., 2009), whereas mutant *gts1* showed quicker growth in young plant and biomass accumulation (Gachomo et al., 2014). Similar negative regulation was shown in Transducin-like gene *AtHOS15*, and the transcript HOS15 with WD40-repeats was identified as a negative regulator of cold stress-responsive genes in *Arabidopsis* (Zhu et al., 2008).

All this information was similar to the presented results in the current study; expression of the *Transducin-1* gene with WD40 repeats, Glyma.04G112700, from QTN1-Yield, showed differential expressions. Low-yielding and more sensitive soybean accessions were shown to have a very high level of gene upregulation under drought, whereas the gene repression with significantly lesser mRNA production was obvious and recorded in drought tolerant and high-yielding soybean cultivars (**Figure 9C**). In contrast, gene *OsLIS-L1* (Lissencephaly type-1-like 1) with WD40 repeats in rice was reported to be directly involved in plant height, and two mutant *oslis-l1* plants had a semi-dwarf phenotype (Gao et al., 2012). These published results can confirm that the *Transducin-2* gene with WD40 repeats, identified in the current study, Glyma.17G093400 from QTN4-PH, has a very similar function for plant height in soybean cultivars, which was confirmed in the gene expression analysis (**Figure 9D**).

QTN7-SWP in the current study was identified as one of the most important with the Glyma.15G156600 gene, which encodes the pentatricopeptide repeat (*PPR*) protein (**Tables 3** and **5**). Importantly, the *PPR* gene 15G156600 was also confirmed during BSA of breeding lines in both hybrids LS1 and LZ2 (**Table 4**). Additional homologous gene Glyma.19G151300 was identified from QTN5-PH for plant height but not selected for further analysis. Most of *PPR* genes are mitochondrial and chloroplast-derived, and they represent one of the largest gene families in plants, and in maize, 456–491 *PPR* genes were found and reported. Eight *PPR* genes located earlier in meta-QTL regions and two more *PPR* genes were identified as associated with yield and kernel-related traits in maize (Chen et al., 2018). It was reported that many *PPR* genes are involved in the regulation of plant responses to abiotic stresses. For example, in *Arabidopsis*, upregulation of PPR-type *SOAR1* gene expression enhanced ABA-induced stomatal closure resulting in improved plant tolerance to multiple abiotic stresses, including drought (Jiang et al., 2015), whereas other *Arabidopsis* genes, *SLG1* and *PPR96*, with mitochondria-localised polypeptides, were shown responsive to drought, salinity, and oxidative stress (Yuan and Liu, 2012; Liu et al., 2016a). Particularly in soybean, one group of *PPR* genes was mapped to all 20 chromosomes and accounted for 179 genes, including Glyma.15G156600 from the current study, and three *PPR* genes were reported highly upregulated based on the drought- and salt-induced transcriptome database (Su et al., 2019). Additionally, the application of synthetic PPR proteins was reported to be a valuable tool for controlling the expression of chloroplast and mitochondrial transcripts in plants (van der Giezen et al., 2024). In the current study, significant differences in the *PPR* gene 15G156600 expression in three high-yielding cultivars showed a strong association with drought tolerance (**Figure 9E**) and can be considered as an important targeting gene for further MAS approaches.

The gene Glyma.15G092400 encoding an ATP-binding cassette (ABC) transporter protein, class C, was identified for both QTN6-SWP and QTN2-PH, indicating that these traits together are involved in drought tolerance and higher seed yield production in soybean plants in dry field trials (**Tables 3** and **5**) and verified in hybrid LZ2 (**Table 4**). The *ABCC* gene family is well known in various plant species (Klein et al., 2006; Wanke and Kolukisaoglu, 2010) and is actively involved in stomatal opening during dehydration in the example of *Arabidopsis* (Klein et al., 2004; Nagy et al., 2009; Gaedeke et al., 2001). In chickpea, gene *GmABCC* was shown to be involved in seed weight and yield (Basu et al., 2019), and it can regulate plant tolerance to salinity and oxidative stress (Khassanova et al., 2024). In the current study, a high level of expression of *GmABCC* gene Glyma.15G092400 in soybean plants was significantly associated with their response to dehydration, confirming their potential role as an important gene for drought tolerance (**Figure 9F**).

The gene Glyma.05G163000, *NTR1.2*, nitrate transporter, identified in QTN1-NPN for number of productive nodes (**Tables 3** and **5**) was confirmed in hybrid population LS1 using BSA (**Table 4**). The *NRT1* gene mediated nitrate transport signalling for the improvement of nitrogen use efficiency in the plant, which can be reflected in more productive nodes in soybean and other legumes. Additionally, recent studies reported that the *NRT1* gene was extensively involved in plant tolerance to various environmental conditions and abiotic stresses, including drought (Fang et al., 2021; Nedelyaeva et al., 2024). Our results for RT-qPCR indicated for a significant association of the gene expression with drought tolerance despite relatively high variability and error bars of the results (**Figure 9G**).

The last selected and studied QTN8-HFP was associated with candidate gene Glyma.18G029000 encoding auxin transporter (*AUXT1*), and the auxin-regulated mechanism of HFP was reported in legumes (Kuzbakova et al., 2022). Gene interaction networks for auxin influx and transporters are very diverse and involved in tolerance to abiotic stresses and in plant organ development and morphogenesis in various plant species including soybean (Chai et al., 2016; Gao et al., 2021b; Yang et al., 2021). The *AUXT* gene was also similar to genes identified in comprehensive analysis of soybean recombinant inbred lines using specific-length amplified fragment (SLAF) markers (Jiang et al., 2018). Eight candidate genes were identified in this report involving the control of the HFP in soybean, and all of them were linked with the auxin network, metabolism, and transportation. Similar auxin-mediated genetic control of internode elongation was shown in maize mutants (Avila et al., 2016; Zhang et al., 2019) and in *CmSi* (short internode) in melon (*Cucumis melo* L.) under auxin regulation (Yang et al., 2020). HFP is a very specific gene which was not directly involved in the drought response in soybean plants, and, therefore, there are very variable results indicated for other factors affecting cell elongation in stem internodes, but it was actually associated with real HFP traits in the studied soybean genotypes and production of first pods on the stem (**Figure 9H**).

Finally, GWAS-identified 41 QTN with their corresponding genes provide an important understanding of the multiple genetic control of yield and yield-related traits in soybean plants grown under drought compared with well-watered conditions. Based on combined GWAS and BSA analyses, eight important genes were selected and studied in more detail, and their involvement in the response to drought was verified. All selected genes confirmed their involvement with either up- or downregulations, with and without differences between low- and high-yielding soybean accessions. All presented results in the current study provide new knowledge and background for the development of molecular markers, which can be used for practical application for the production of novel drought tolerant soybean cultivars in dry continental climate conditions of South-Eastern Kazakhstan and in other countries with similar environments.

## Data Availability

The original contributions presented in the study are included in the article/**Supplementary Material**. Further inquiries can be directed to the corresponding authors.
