# Correction: Yield and yield component trait analysis with DArT genotyping for GWAS in soybean grown in drought conditions of Kazakhstan

**DOI:** 10.3389/fpls.2026.1910549

**Published:** 2026-06-22

**Authors:** Aigul Amangeldiyeva, Raushan Yerzhebayeva, Shynar Mazkirat, Svetlana Didorenko, Sholpan Bastaubayeva, Bekzhan Maikotov, Rinat Kassenov, Assel Jenisbayeva, Yuri Shavrukov

**Affiliations:** 1 Kazakh Research Institute of Agriculture and Plant Growing, Almalybak, Kazakhstan; 2 Farabi University, Almaty, Kazakhstan; 3 College of Science and Engineering, Biological Sciences, Flinders University, Adelaide, SA, Australia

**Keywords:** bulk segregant analysis (BSA), candidate genes verification, Diversity array technology (DArT), drought, field trial, gene expression, genome-wide association study (GWAS), molecular genetic dendrogram

In the published article, the additional affiliation ^2^Farabi University, Almaty, Kazakhstan was omitted for author Aigul Amangeldiyeva. The first affiliation remains unchanged. The omitted affiliation has now been added for author Aigul Amangeldiyeva as follow:

“^1^Kazakh Research Institute of Agriculture and Plant Growing, Almalybak, Almaty district, Kazakhstan; ^2^Farabi University, Almaty, Kazakhstan”

The original version of this article has been updated.

