# Supplementary material for: Yield and yield component trait analysis with DArT genotyping for GWAS in soybean grown in drought conditions of Kazakhstan
Source: Front Plant Sci. 2025 Oct 14;16:1674201. doi: 10.3389/fpls.2025.1674201 (PMC12558979; doi:10.3389/fpls.2025.1674201)
Supplement: Supplementary file 3 [file DataSheet3.pdf]

Supplementary material S2. Image of the field trial with 188 soybean accessions grown simultaneously in both well-watered WW (irrigated) field, framed by the blue line, and in conditions of drought stress, DS (non-irrigated), framed by the yellow line. The experiment was carried out in research fields of the Kazakh Research Institute of Agriculture and Plant Growing (KRIAPG), Almaty region, Kazakhstan in 2024.

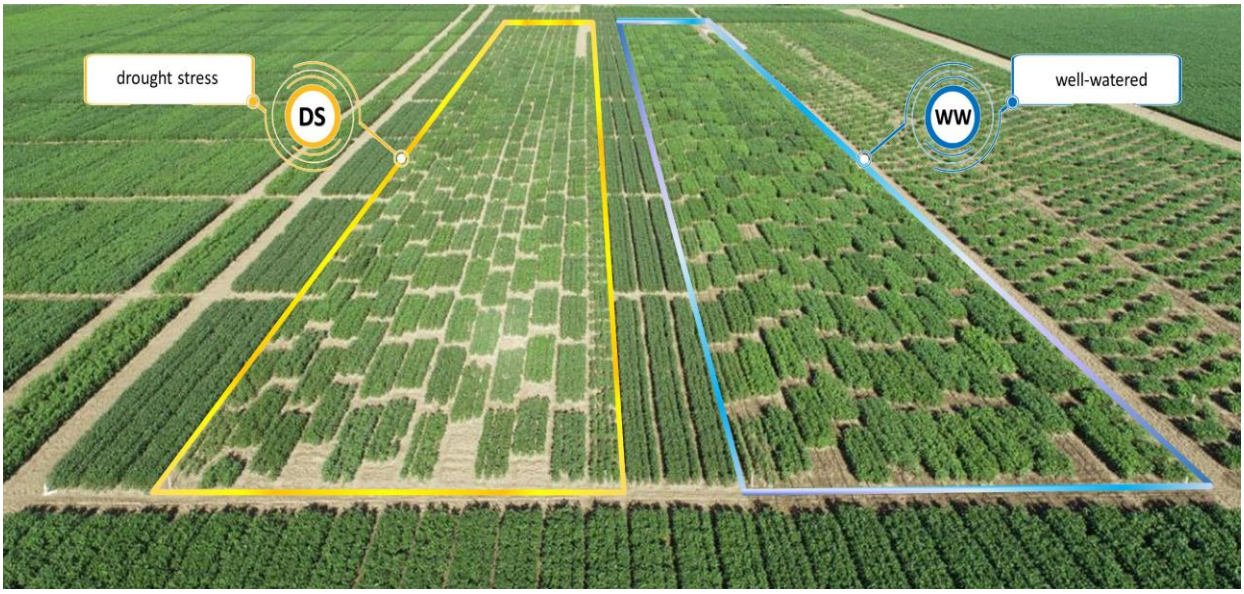

Supplementary material S4. Sequences of the used primers with gene identification

Name	Gene ID	Sequence 5'-3'	Amplicon, bp
Rab-GDI2	Glyma.08G126200	F: TGATCCATCATATTTGCCCGGC	145
		R: TGACTTCCGACCCAACTGTTTC	
Tr-WD40-1	Glyma.04G112700	F: GTTGGAGGATTTCAGGGTGAAC	150
		R: AGCGGCTGTGAAATGAACTGCA	
Tr-WD40-2	Glyma.17G093400	F: CAAACTCCTCGTCCCCAAAAAC	113
		R: AGAAGTTTCCGTCTTGGGACAG	
G6PI	Glyma.06G032500	F: GAGAAGTTTGCCCCACACATTC	136
		R: AGCTATGCTGCCCATTAGTTCC	
PPR	Glyma.15G156600	F: ATGCATGGGAAAGGGGAAGAAG	157
		R: ACAAATGCAGGCCTTCATCCAC	
ABCC	Glyma.15G092400	F: GGTGGCCAAAAGCAGAGAGTAT	143
		R: CCTCTCAAATCTCCCTTGATGC	
NTR1.2	Glyma.05G163000	F: TCAACAGGACAAAGAAGCCGCA	147
		R: ACTCTGCATAGTTTCCACGGGT	
AUXT1	Glyma.18G029000	F: TATTTGGAGCTTGCTGTGCCAC	127
		R: TGAACAAGGGCTGCAATGGTCA	
ACT11	Glyma.18G52780	F: ATCTTGACTGAGCGTGGTTATTCC	126
		R: GCTGGTCCTGGCTGTCTCC	

Supplementary material S5. Descriptive statistics analysis of yield, yield components and vegetation index NDVI in 188 soybean accessions grown in field trials with and without irrigation (well watering, WW, and drought stress, DS, respectively) in South-Eastern Kazakhstan.

Trait	Factor	Statistical parameters of studied					Reduction ratio DS/WW, %
		Mean	St.Dev.	CV	Min	Max	
Seed yield in plots (Y)	DS	2.6	1.3	0.5	0.3	6.3	34.4
	WW	4.0	1.3	0.3	1.1	7.6	
Seed weight per plant (SWP)	DS	11.2	4.7	0.4	1.3	36.5	35.7
	WW	17.4	8.5	0.5	1.0	43.5	
Pod number per plant (PNP)	DS	37.9	11.2	0.3	12.0	96.0	25.7
	WW	51.0	20.1	0.4	10.0	118,0	
Number of productive nodes (NPN)	DS	16.8	4.9	0.3	3.6	50.0	14.8
	WW	19.7	6.6	0.3	4.4	52.0	
Number of side branches (NSB)	DS	1.3	1.1	0.8	0.0	6.0	7.2
	WW	1.4	1.0	0.7	0.0	7.70	
Plant height (PH)	DS	80.7	26.1	0.3	26.0	145.9	20.6
	WW	101.7	25.9	0.3	32.0	185.0	
Height to first pod (HFP)	DS	15.7	4.5	0.3	5.0	36.0	-8.0
	WW	14.5	4.6	0.3	6.0	32.8	
Thousand seed weight (TSW)	DS	162.4	25.1	0.2	103.3	228.0	12.1
	WW	184.8	27.2	0.2	98.3	352.0	
Normalized difference vegetation index (NDVI)	DS	0.7	0.1	0.1	0.4	0.8	9.5
	WW	0.7	0.1	0.1	0.5	0.9	

Supplementary material S6. Identified 41 QTN and relevant putative candidate genes in soybean germplasm accessions based on GWAS analysis of marker-trait associations (MTA) between 16K filtered DArT markers and nine major yield and yield-related traits in plants grown in two environments with regular and limited irrigation (well-watering and drought), during two years (2023 and 2024) in South-Eastern Kazakhstan. Detection methods: G, General Linear Model (GLM); M. Multiple Loci Mixed Model (MLMM); F, Fixed and Random Model Circulating Probability Unification (FarmCPU); B, Linkage-Disequilibrium Iteratively Nested Keyway (Blink).

QTN	DArT clone	Chr	Clone position	Relevant gene	Gene position	Annotated description	P.value	MAF	Detec. method	Treatm. & Year
**Yield**										
QTN1-Yield	14967925	4	11,826,820	Glyma. 04G112700	11,823,184-11,828,101	Transducin with WD40 repeats protein	1.206e-06 - 2.449e-11	0.301	M,F	Drought, 2024
QTN2-Yield	14970391	6	2,517,628	Glyma. 06G032500	2,514,935-2,519,671	Glucose-6-phosphate isomerase (G6PI=PGI)	1.079e-06 - 3.527e-07	0.161	F,B	Drought, 2023
QTN3-Yield	100499705	8	9,754,335	Glyma. 08G126200	9,753,912-9,759,348	Rab-GDP dissociation inhibitor 2 (Rab-GDI2)	7.813e-07	0.212	B	Drought, 2023
QTN4-Yield	14969881	18	53,634,338	Glyma 18G222500	53,626,652-53,641,652	Titan-9	1.335e-06	0.276	M,B	Drought, 2023
QTN5-Yield	14982561	18	54,205,434	Glyma. 18G226500	54,205,371-54,275,961	NB-ARC domain disease resistance protein (NB-ARC)	3.218e-07 - 9.919e-07	0.218	F,B	Drought, 2023
**Seeds weight per plant (SWP)**										
QTN1-SWP	14982846	6	47,684,603	Glyma. 06G266500	47,545,898-47,552,336	ATP binding microtubule motor protein (m.tubul-motor)	1.076e-09	0.481	M	Drought, 2024
QTN2-SWP	100499705	8	9,754,335	Glyma. 08G126200	9,753,912-9,759,348	Rab-GDP dissociation inhibitor 2 (Rab-GDI2)	4.102e-09	0.199	F	Drought, 2023
QTN3-SWP	14977304	12	38,436,659	Glyma. 12G188100	38,435,948-38,440,478	Protein kinase protein	4.676e-06	0.433	M	Drought, 2024
QTN4-SWP	50677764	14	9,729,961	Glyma. 14G098900	9,748,407-9,757,546	F-box/ Leucine-rich repeat protein 15 (F-box- LRR)	4.960e-08	0.327	F	Drought, 2023
QTN5-SWP	14972694	14	13,522,469	Glyma. 14G112700	13,497,477-13,517,410	Histone-lysine N-methyl-transferase (SUVR2)	2.693e-14	0.135	M	WW, 2024
QTN6-SWP	14978704	15	7,106,934	Glyma. 15G092400	7,099,029-7,120,507	ATP-binding cassette (ABC) transporter protein	2.148e-07	0.298	B	Drought, 2023
QTN7-SWP	14981189	15	13,118,452	Glyma. 15G156600	13,118,431-13,121,292	Pentatricopeptide repeat (PPR) protein	1.497e-06	0.254	B	Drought, 2023
QTN8-SWP	22920979	20	50,140,446	Glyma. 20G238500	50,140,311-50,143,656	Pre-mRNA-splicing factor	8.252e-06	0.317	B	WW, 2023

**Pod number per plant (PNP)**										
QTN1-PNP	14981804	6	45,562,607	No any gene found	-	No any gene found	9.898e-08	0.309	F	WW, 2023
QTN2-PNP	50683668	9	44,728,755	Glyma. 09G208200	44,726,180-44,731,616	Cellulose synthase D3 (CS-D3)	1.108e-06	0.338	B	Drought, 2023
**Number of productive nodes (NPN)**										
QTN1-NPN	14969732	5	37,651,079	Glyma. 05G163000	37,634,948-37,639,388	Nitrate transporter 1.2 (NTR1.2)	5.445e-14	0.121	F	WW, 2024
QTN2-NPN	100481948	17	42,808,919	Glyma. 17G248400	42,784,838-42,794,814	Metalloendopeptidase-zinc ion-binding protein	7.358e-07 - 9.160e-12	0.289	F,B	Drought, 2023
**Number of side branches (NSB)**										
QTN1-NSB	14969678	3	5,524,723	Glyma. 03G041600	5,520,500-5,528,728	Protein SCAR2-like	2.143e-08	0.240	F	Drought, 2023
QTN2-NSB	14970470	4	2,315,413	Glyma. 04G028600	2,316,665-2,322,256	Beta-galactosidase 3	1.289e-07	0.194	B	Drought, 2024
**Plant height (PH)**										
QTN1-PH	14970055	7	43,741	Glyma. 07G000400	42,094-58,689	Histone acetyltransferase HAC1	8.800e-07	0.110	B	Drought, 2023
QTN2-PH	14978704	15	7,106,934	Glyma. 15G092400	7,099,029-7,120,507	ATP-binding cassette (ABC) transporter protein	1.491e-06 - 1.454e-08	0.298	G,M,B	Drought, 2023
QTN3-PH	14983750	16	35,880,652	Glyma. 16G176600	35,886,356-35,894,264	Receptor-like protein kinase 2	2.517e-07 - 1.426e-09	0.434	M,F	Drought, 2024
QTN4-PH	29305538	17	7,310,902	Glyma. 17G093400	7,310,578-7,312,266	Transducin with WD40 repeats protein	1.307e-07	0.358	F	Drought, 2023
QTN5-PH	14975485	19	44,252,259	Glyma. 19G151300	44,257,603-44,259,910	Pentatricopeptide repeat (PPR-like) protein	1.320e-06	0.494	B	WW 2023
**Height to first pod (HFP)**										
QTN1-HFP	14975791	4	4,467,835	Glyma. 04G055200	4,460,129-4,464,132	Mechanosensitive ion channel protein	1.100e-06	0.172	F	Drought, 2024
QTN2-HFP	14968153	4	6,944,135	Glyma. 04G082700	6,951,452-6,962,443	Peptide transporter 1	2.799e-07 - 2.520e-07	0.249	F,B	Drought, 2023
QTN3-HFP	14972262	8	18,361,859	Glyma. 08G225302 (or another)	18,361,851-18,366,217	P-loop containing nucleoside triphosphate hydrolases protein (P-NTPH)	5.337e-09	0.243	M	Drought, 2023
QTN4-HFP	22920754	15	9,155,744	Glyma. 15G117100	9,160,663-9,166,841	Myb DNA-binding domain protein	3.352e-07	0.071	F	Drought, 2024
QTN5-HFP	29305595	15	34,313,660	Glyma. 15G211600	34,305,648-34,308,370	PHD finger protein	5.820e-07 - 1.145e-07	0.205	M,F,B	Drought, 2024

QTN6-HFP	14974197	15	46,121,624	Glyma. 15G233400	46,174,045-46,179,394	NB-ARC domain disease resistance protein (NB-ARC)	1.432e-06	0.194	B	WW, 2023
QTN7-HFP	14969945	16	2,411,858	Glyma. 16G024900	2,405,982-2,414,133	Histone-lysine N-methyltransferase ATX3	6.227e-07	0.338	B	Drought, 2023
QTN8-HFP	14969998	18	2,219,949	Glyma. 18G029000	2,200,543-2,206,490	Auxin transporter protein 1 (AUXT1)	8.174e-10 - 2.971e-07	0.075	M,F,B	Drought, 2023
QTN9-HFP	14980986	19	52,886,557	Glyma. 19G251700	52,885,119-52,890,402	Essential nucleolar protein, small subunit processome	3.971e-07	0.368	B	WW 2024
**Thousand seeds weight (TSW)**										
QTN1-TSW	14965896	6	3,137,583	Glyma. 06G041400	3,133,973-3,137,754	HXXXD-acyl-transferase protein	7.668e-09	0.057	F	Drought, 2023
QTN2-TSW	14976250	16	32780857	Glyma. 16G146900	32,782,187-32,785,178	Phosphoglycerate mutase protein	2.035e-10	0.060	B	Drought, 2024
QTN3-TSW	24388245	17	41,930,334	Glyma. 17G239500	41,935,411-41,954,004	ATP binding microtubule motor protein (m.tubul-motor)	1.159e-06	0.105	F	Drought, 2023
**Normalized difference vegetation index (NDVI)**										
QTN1-NDVI	24386134	2	44,959,613	Glyma. 02G202500	44,951,358-44,955,905	Aldehyde dehydrogenase 2, member C4-like (ADH-C4)	1.192e-07	0.070	M	WW, 2024
QTN2-NDVI	14970403	4	2,588,144	Glyma. 04G032600	2,585,516-2,595,004	Glucose-6-phosphate isomerase (G6PI=PGI)	9.103e-08 - 2.432e-09	0.110	M,B	Drought, 2023
QTN3-NDVI	14972262	8	18,361,859	Glyma. 08G225302	18,361,851-18,366,217	P-loop containing nucleoside triphosphate hydrolases protein (P-NTPH)	1.118e-06 - 5.256e-11	0.243	G,M,F,B	Drought, 2023
QTN4-NDVI	14983239	17	38,288,541	Glyma. 17G214100	38,291,147-38,294,163	Receptor-like protein kinase 4	1.437e-06	0.488	B	Drought, 2023
QTN5-NDVI	86239108	18	8,894,374	Glyma. 18G089500	8,897,657-8,903,847	Transcription initiation factor TFIID subunit	3.160e-10	0.364	F	Drought, 2023

Supplementary material S7. Manhattan Plots and Q-Q plots of the association analysis in the water deficit experiment (drought) using the BLINK model. The positions on chromosomes are shown on the X-axis, whereas values of SNPs are shown on the Y-axis in logarithmic scale. Dashed line indicates threshold level of significance (-log10 P-value = 6.0-15.0). Identified significant DArT markers are shown at the top by arrows for the corresponding traits: **(A)** SWP, Seed weight per plant; **(B)** HFP, Height to first pod; **(C)** PH, Plant height; **(D)** NPN, Number of productive nodes; **(E)** NDVI, Normalized difference vegetation index; **(F)** Y, Yield. Identification of DArT markers is present in Table 3.

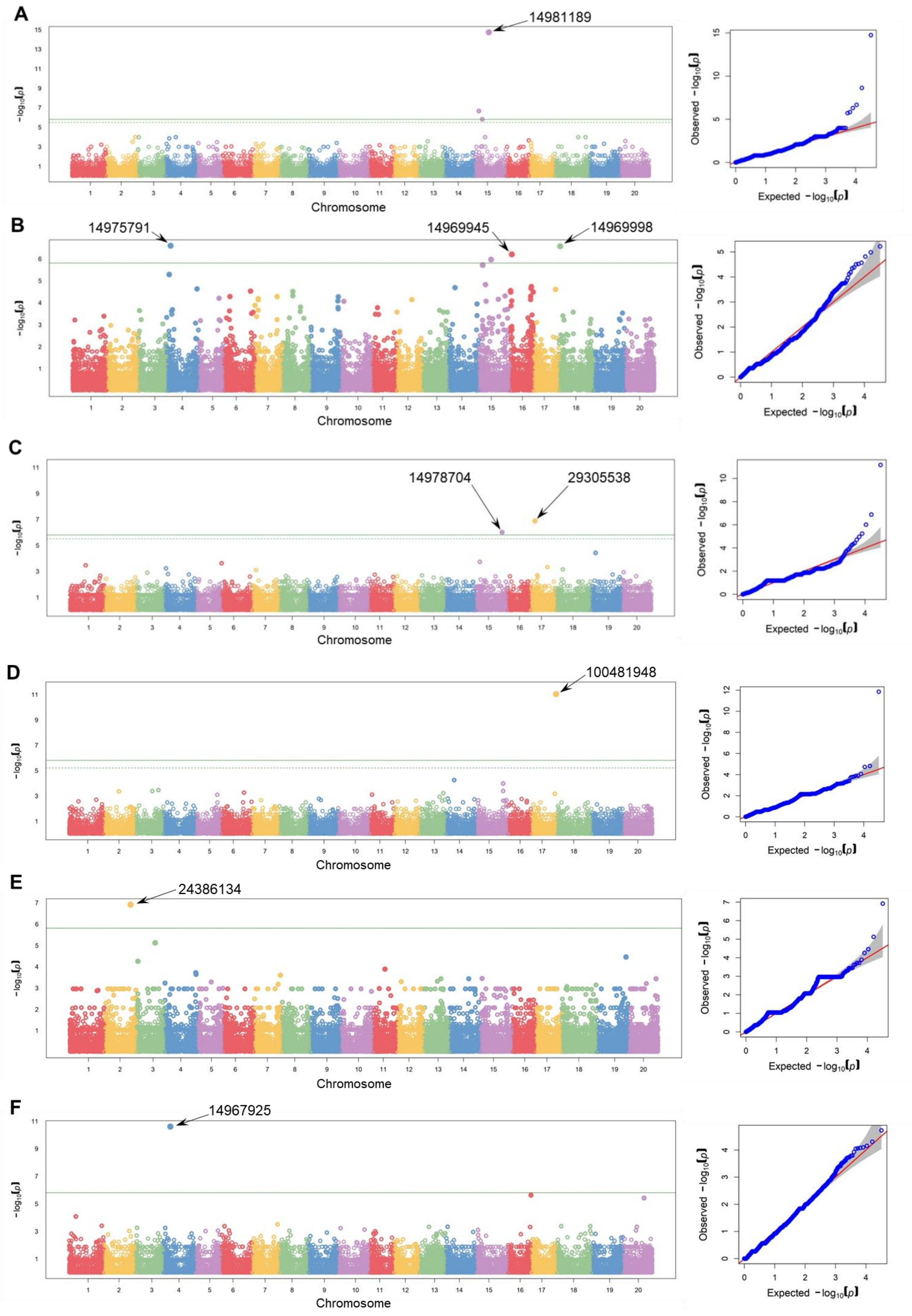

Supplementary material S8. Details of statistical treatments of RT-qPCR analysis for one-way ANOVA and post-hoc Tukey HSD test.

(A) Gene Gm08g126200, Rab-GDP dissociation inhibitor 2 (*Rab-GDI2*)

WW-TP1, Well-watered, Time-point 1:

ANOVA Table...

Source of Variation	Sum of Squares	d.f.	Variance	F	p
Between Groups:	0.1450	5	0.0290	0.4828	0.7827
Within Groups:	0.7210	12	0.0601		
Total:	0.8660	17			

Post-hoc tests...

```
Tukey HSD Post-hoc Test...
Vilana vs Zen: Diff=-0.0677, 95%CI=-0.7399 to 0.6045, p=0.9992
Vilana vs Sponsor: Diff=-0.0036, 95%CI=-0.6759 to 0.6686, p=0.9940
Vilana vs Kye-shuan: Diff=-0.0600, 95%CI=-0.7323 to 0.6122, p=0.9996
Vilana vs Czi-ti-4: Diff=0.1737, 95%CI=-0.4985 to 0.8460, p=0.9473
Vilana vs Lastochka: Diff=0.1198, 95%CI=-0.5524 to 0.7920, p=0.9891
Zen vs Sponsor: Diff=0.0641, 95%CI=-0.6082 to 0.7363, p=0.9994
Zen vs Kye-shuan: Diff=0.0077, 95%CI=-0.6646 to 0.6799, p=1.0000
Zen vs Czi-ti-4: Diff=0.2414, 95%CI=-0.4308 to 0.9137, p=0.8260
Zen vs Lastochka: Diff=0.1875, 95%CI=-0.4847 to 0.8597, p=0.9290
```

Tukey HSD Post-hoc Test:

Vilana vs Zen: Diff=-0.0677, 95%CI=-0.7399 to 0.6045, p=0.9992
Vilana vs Sponsor: Diff=-0.0036, 95%CI=-0.6759 to 0.6686, p=0.9940
Vilana vs Kye-shuan: Diff=-0.0600, 95%CI=-0.7323 to 0.6122, p=0.9996
Vilana vs Czi-ti-4: Diff=0.1737, 95%CI=-0.4985 to 0.8460, p=0.9473
Vilana vs Lastochka: Diff=0.1198, 95%CI=-0.5524 to 0.7920, p=0.9891
Zen vs Sponsor: Diff=0.0641, 95%CI=-0.6082 to 0.7363, p=0.9994
Zen vs Kye-shuan: Diff=0.0077, 95%CI=-0.6646 to 0.6799, p=1.0000
Zen vs Czi-ti-4: Diff=0.2414, 95%CI=-0.4308 to 0.9137, p=0.8260
Zen vs Lastochka: Diff=0.1875, 95%CI=-0.4847 to 0.8597, p=0.9290
Sponsor vs Kye-shuan: Diff=-0.0564, 95%CI=-0.7287 to 0.6158, p=0.9997
Sponsor vs Czi-ti-4: Diff=0.1773, 95%CI=-0.4949 to 0.8496, p=0.9428
Sponsor vs Lastochka: Diff=0.1234, 95%CI=-0.5488 to 0.7957, p=0.9876
Kye-shuan vs Czi-ti-4: Diff=0.2338, 95%CI=-0.4385 to 0.9060, p=0.8434
Kye-shuan vs Lastochka: Diff=0.1798, 95%CI=-0.4924 to 0.8521, p=0.9396
Czi-ti-4 vs Lastochka: Diff=-0.0539, 95%CI=-0.7262 to 0.6183, p=0.9997

WW-TP2, Well-watered, Time-point 2:

ANOVA Table...

Source of Variation	Sum of Squares	d.f.	Variance	F	p
Between Groups:	0.0723	5	0.0145	0.1473	0.9770
Within Groups:	1.1786	12	0.0982		
Total:	1.2510	17			

Post-hoc tests...

```
Tukey HSD Post-hoc Test...
Vilana vs Zen: Diff=0.0609, 95%CI=-0.7986 to 0.9204, p=0.9998
Vilana vs Sponsor: Diff=0.1188, 95%CI=-0.7408 to 0.9783, p=0.9966
Vilana vs Kye-shuan: Diff=0.0386, 95%CI=-0.8210 to 0.8981, p=1.0000
Vilana vs Czi-ti-4: Diff=0.1521, 95%CI=-0.7075 to 1.0116, p=0.9895
Vilana vs Lastochka: Diff=0.1786, 95%CI=-0.6809 to 1.0382, p=0.9786
Zen vs Sponsor: Diff=0.0578, 95%CI=-0.8017 to 0.9174, p=0.9999
Zen vs Kye-shuan: Diff=-0.0224, 95%CI=-0.8819 to 0.8372, p=1.0000
Zen vs Czi-ti-4: Diff=0.0911, 95%CI=-0.7684 to 0.9507, p=0.9990
Zen vs Lastochka: Diff=0.1177, 95%CI=-0.7418 to 0.9773, p=0.9967
```

Tukey HSD Post-hoc Test:

Vilana vs Zen: Diff=0.0609, 95%CI=-0.7986 to 0.9204, p=0.9998
Vilana vs Sponsor: Diff=0.1188, 95%CI=-0.7408 to 0.9783, p=0.9966
Vilana vs Kye-shuan: Diff=0.0386, 95%CI=-0.8210 to 0.8981, p=1.0000
Vilana vs Czi-ti-4: Diff=0.1521, 95%CI=-0.7075 to 1.0116, p=0.9895
Vilana vs Lastochka: Diff=0.1786, 95%CI=-0.6809 to 1.0382, p=0.9786
Zen vs Sponsor: Diff=0.0578, 95%CI=-0.8017 to 0.9174, p=0.9999
Zen vs Kye-shuan: Diff=-0.0224, 95%CI=-0.8819 to 0.8372, p=1.0000
Zen vs Czi-ti-4: Diff=0.0911, 95%CI=-0.7684 to 0.9507, p=0.9990
Zen vs Lastochka: Diff=0.1177, 95%CI=-0.7418 to 0.9773, p=0.9967
Sponsor vs Kye-shuan: Diff=-0.0802, 95%CI=-0.9397 to 0.7793, p=0.9995
Sponsor vs Czi-ti-4: Diff=0.0333, 95%CI=-0.8262 to 0.8928, p=1.0000
Sponsor vs Lastochka: Diff=0.0599, 95%CI=-0.7996 to 0.9194, p=0.9999
Kye-shuan vs Czi-ti-4: Diff=0.1135, 95%CI=-0.7460 to 0.9730, p=0.9973
Kye-shuan vs Lastochka: Diff=0.1401, 95%CI=-0.7194 to 0.9996, p=0.9927
Czi-ti-4 vs Lastochka: Diff=0.0266, 95%CI=-0.8329 to 0.8861, p=1.0000

Dr-TP1, Drought, Time-point 1:

ANOVA Table...

Source of Variation	Sum of Squares	d.f.	Variance	F	p
Between Groups:	1.2017	5	0.2403	12.6530	0.0002
Within Groups:	0.2279	12	0.0190		
Total:	1.4297	17			

Post-hoc tests...

```
Zen vs Kye-shuan: Diff=0.2937, 95%CI=-0.0843 to 0.6717, p=0.1681
Zen vs Czi-ti-4: Diff=0.4496, 95%CI=0.0716 to 0.8276, p=0.0171
Zen vs Lastochka: Diff=0.2744, 95%CI=-0.1036 to 0.6524, p=0.2175
Sponsor vs Kye-shuan: Diff=0.5356, 95%CI=0.1577 to 0.9136, p=0.0048
Sponsor vs Czi-ti-4: Diff=0.6915, 95%CI=0.3135 to 1.0695, p=0.0006
Sponsor vs Lastochka: Diff=0.5163, 95%CI=0.1383 to 0.8943, p=0.0063
Kye-shuan vs Czi-ti-4: Diff=0.1559, 95%CI=-0.2221 to 0.5339, p=0.7349
Kye-shuan vs Lastochka: Diff=-0.0193, 95%CI=-0.3973 to 0.3587, p=1.0000
Czi-ti-4 vs Lastochka: Diff=-0.1752, 95%CI=-0.5532 to 0.2028, p=0.6382
```

Tukey HSD Post-hoc Test:

Vilana vs Zen: Diff=0.1891, 95%CI=-0.1889 to 0.5671, p=0.5675
Vilana vs Sponsor: Diff=-0.0529, 95%CI=-0.4309 to 0.3251, p=0.9964
Vilana vs Kye-shuan: Diff=0.4828, 95%CI=0.1048 to 0.8608, p=0.0104
Vilana vs Czi-ti-4: Diff=0.6386, 95%CI=0.2606 to 1.0166, p=0.0011
Vilana vs Lastochka: Diff=0.4634, 95%CI=0.0854 to 0.8414, p=0.0139
Zen vs Sponsor: Diff=-0.2419, 95%CI=-0.6199 to 0.1360, p=0.3260
Zen vs Kye-shuan: Diff=0.2937, 95%CI=-0.0843 to 0.6717, p=0.1681
Zen vs Czi-ti-4: Diff=0.4496, 95%CI=0.0716 to 0.8276, p=0.0171
Zen vs Lastochka: Diff=0.2744, 95%CI=-0.1036 to 0.6524, p=0.2175
Sponsor vs Kye-shuan: Diff=0.5356, 95%CI=0.1577 to 0.9136, p=0.0048
Sponsor vs Czi-ti-4: Diff=0.6915, 95%CI=0.3135 to 1.0695, p=0.0006
Sponsor vs Lastochka: Diff=0.5163, 95%CI=0.1383 to 0.8943, p=0.0063
Kye-shuan vs Czi-ti-4: Diff=0.1559, 95%CI=-0.2221 to 0.5339, p=0.7349
Kye-shuan vs Lastochka: Diff=-0.0193, 95%CI=-0.3973 to 0.3587, p=1.0000
Czi-ti-4 vs Lastochka: Diff=-0.1752, 95%CI=-0.5532 to 0.2028, p=0.6382

Dr-TP2, Drought, Time-point 2:

ANOVA Table...

Source of Variation	Sum of Squares	d.f.	Variance	F	p
Between Groups:	43.9803	5	8.7961	4.6132	0.0140
Within Groups:	22.8807	12	1.9067		
Total:	66.8610	17			

Post-hoc tests...

```
Tukey HSD Post-hoc Test...
Vilana vs Zen: Diff=0.1099, 95%CI=-3.6772 to 3.8970, p=1.0000
Vilana vs Sponsor: Diff=0.4573, 95%CI=-3.3298 to 4.2443, p=0.9982
Vilana vs Kye-shuan: Diff=2.1273, 95%CI=-1.6598 to 5.9144, p=0.4532
Vilana vs Czi-ti-4: Diff=3.5563, 95%CI=-0.2308 to 7.3434, p=0.0703
Vilana vs Lastochka: Diff=3.7193, 95%CI=-0.0678 to 7.5064, p=0.0553
Zen vs Sponsor: Diff=0.3473, 95%CI=-3.4398 to 4.1344, p=0.9995
Zen vs Kye-shuan: Diff=2.0174, 95%CI=-1.7697 to 5.8045, p=0.5060
Zen vs Czi-ti-4: Diff=3.4464, 95%CI=-0.3407 to 7.2335, p=0.0825
Zen vs Lastochka: Diff=3.6094, 95%CI=-0.1777 to 7.3965, p=0.0650
```

Tukey HSD Post-hoc Test:

Vilana vs Zen: Diff=0.1099, 95%CI=-3.6772 to 3.8970, p=1.0000
Vilana vs Sponsor: Diff=0.4573, 95%CI=-3.3298 to 4.2443, p=0.9982
Vilana vs Kye-shuan: Diff=2.1273, 95%CI=-1.6598 to 5.9144, p=0.4532
Vilana vs Czi-ti-4: Diff=3.5563, 95%CI=-0.2308 to 7.3434, p=0.0703
Vilana vs Lastochka: Diff=3.7193, 95%CI=-0.0678 to 7.5064, p=0.0553
Zen vs Sponsor: Diff=0.3473, 95%CI=-3.4398 to 4.1344, p=0.9995
Zen vs Kye-shuan: Diff=2.0174, 95%CI=-1.7697 to 5.8045, p=0.5060
Zen vs Czi-ti-4: Diff=3.4464, 95%CI=-0.3407 to 7.2335, p=0.0825
Zen vs Lastochka: Diff=3.6094, 95%CI=-0.1777 to 7.3965, p=0.0650
Sponsor vs Kye-shuan: Diff=1.6701, 95%CI=-2.1170 to 5.4572, p=0.6814
Sponsor vs Czi-ti-4: Diff=3.0991, 95%CI=-0.6880 to 6.8862, p=0.1355
Sponsor vs Lastochka: Diff=3.2620, 95%CI=-0.5250 to 7.0491, p=0.1076
Kye-shuan vs Czi-ti-4: Diff=1.4290, 95%CI=-2.3581 to 5.2161, p=0.7964
Kye-shuan vs Lastochka: Diff=1.5920, 95%CI=-2.1951 to 5.3791, p=0.7201
Czi-ti-4 vs Lastochka: Diff=0.1630, 95%CI=-3.6241 to 3.9501, p=1.0000

(B) Glyma.06G032500, Glucose-6-phosphate isomerase (*G6PI=PGI*)

WW-TP1, Well-watered, Time-point 1:

ANOVA Table...

Source of Variation	Sum of Squares	d.f.	Variance	F	p
Between Groups:	0.2340	5	0.0468	1.1244	0.3986
Within Groups:	0.4995	12	0.0416		
Total:	0.7335	17			

Post-hoc tests...

```
Tukey HSD Post-hoc Test...
Vilana vs Zen: Diff=-0.1995, 95%CI=-0.7590 to 0.3601, p=0.8301
Vilana vs Sponsor: Diff=0.0306, 95%CI=-0.5290 to 0.5901, p=0.9999
Vilana vs Kye-shuan: Diff=-0.2782, 95%CI=-0.8377 to 0.2814, p=0.5734
Vilana vs Czi-ti-4: Diff=-0.1898, 95%CI=-0.7494 to 0.3698, p=0.8559
Vilana vs Lastochka: Diff=-0.2049, 95%CI=-0.7644 to 0.3547, p=0.8148
Zen vs Sponsor: Diff=0.2300, 95%CI=-0.3295 to 0.7896, p=0.7372
Zen vs Kye-shuan: Diff=-0.0787, 95%CI=-0.6383 to 0.4809, p=0.9963
Zen vs Czi-ti-4: Diff=0.0097, 95%CI=-0.5499 to 0.5692, p=1.0000
Zen vs Lastochka: Diff=-0.0054, 95%CI=-0.5650 to 0.5542, p=1.0000
```

Tukey HSD Post-hoc Test:

Vilana vs Zen: Diff=-0.1995, 95%CI=-0.7590 to 0.3601, p=0.8301
Vilana vs Sponsor: Diff=0.0306, 95%CI=-0.5290 to 0.5901, p=0.9999
Vilana vs Kye-shuan: Diff=-0.2782, 95%CI=-0.8377 to 0.2814, p=0.5734
Vilana vs Czi-ti-4: Diff=-0.1898, 95%CI=-0.7494 to 0.3698, p=0.8559
Vilana vs Lastochka: Diff=-0.2049, 95%CI=-0.7644 to 0.3547, p=0.8148
Zen vs Sponsor: Diff=0.2300, 95%CI=-0.3295 to 0.7896, p=0.7372
Zen vs Kye-shuan: Diff=-0.0787, 95%CI=-0.6383 to 0.4809, p=0.9963
Zen vs Czi-ti-4: Diff=0.0097, 95%CI=-0.5499 to 0.5692, p=1.0000
Zen vs Lastochka: Diff=-0.0054, 95%CI=-0.5650 to 0.5542, p=1.0000
Sponsor vs Kye-shuan: Diff=-0.3087, 95%CI=-0.8683 to 0.2508, p=0.4711
Sponsor vs Czi-ti-4: Diff=-0.2204, 95%CI=-0.7799 to 0.3392, p=0.7681
Sponsor vs Lastochka: Diff=-0.2354, 95%CI=-0.7950 to 0.3241, p=0.7194
Kye-shuan vs Czi-ti-4: Diff=0.0884, 95%CI=-0.4712 to 0.6479, p=0.9937
Kye-shuan vs Lastochka: Diff=0.0733, 95%CI=-0.4863 to 0.6329, p=0.9974
Czi-ti-4 vs Lastochka: Diff=-0.0151, 95%CI=-0.5746 to 0.5445, p=1.0000

WW-TP2, Well-watered, Time-point 2:

ANOVA Table...

Source of Variation	Sum of Squares	d.f.	Variance	F	p
Between Groups:	0.2724	5	0.0545	2.3196	0.1079
Within Groups:	0.2818	12	0.0235		
Total:	0.5542	17			

Post-hoc tests...

```
Tukey HSD Post-hoc Test...
Vilana vs Zen: Diff=-0.2300, 95%CI=-0.6503 to 0.1903, p=0.4794
Vilana vs Sponsor: Diff=-0.0427, 95%CI=-0.4630 to 0.3776, p=0.9992
Vilana vs Kye-shuan: Diff=-0.1250, 95%CI=-0.5453 to 0.2953, p=0.9095
Vilana vs Czi-ti-4: Diff=0.0876, 95%CI=-0.3327 to 0.5079, p=0.9784
Vilana vs Lastochka: Diff=-0.2580, 95%CI=-0.6783 to 0.1623, p=0.3658
Zen vs Sponsor: Diff=0.1873, 95%CI=-0.2330 to 0.6076, p=0.6725
Zen vs Kye-shuan: Diff=0.1049, 95%CI=-0.3154 to 0.5253, p=0.9540
Zen vs Czi-ti-4: Diff=0.3176, 95%CI=-0.1027 to 0.7379, p=0.1875
Zen vs Lastochka: Diff=-0.0280, 95%CI=-0.4483 to 0.3923, p=0.9999
```

Tukey HSD Post-hoc Test:

Vilana vs Zen: Diff=-0.2300, 95%CI=-0.6503 to 0.1903, p=0.4794
Vilana vs Sponsor: Diff=-0.0427, 95%CI=-0.4630 to 0.3776, p=0.9992
Vilana vs Kye-shuan: Diff=-0.1250, 95%CI=-0.5453 to 0.2953, p=0.9095
Vilana vs Czi-ti-4: Diff=0.0876, 95%CI=-0.3327 to 0.5079, p=0.9784
Vilana vs Lastochka: Diff=-0.2580, 95%CI=-0.6783 to 0.1623, p=0.3658
Zen vs Sponsor: Diff=0.1873, 95%CI=-0.2330 to 0.6076, p=0.6725
Zen vs Kye-shuan: Diff=0.1049, 95%CI=-0.3154 to 0.5253, p=0.9540
Zen vs Czi-ti-4: Diff=0.3176, 95%CI=-0.1027 to 0.7379, p=0.1875
Zen vs Lastochka: Diff=-0.0280, 95%CI=-0.4483 to 0.3923, p=0.9999
Sponsor vs Kye-shuan: Diff=-0.0824, 95%CI=-0.5027 to 0.3380, p=0.9835
Sponsor vs Czi-ti-4: Diff=0.1303, 95%CI=-0.2900 to 0.5506, p=0.8949
Sponsor vs Lastochka: Diff=-0.2153, 95%CI=-0.6356 to 0.2050, p=0.5444
Kye-shuan vs Czi-ti-4: Diff=0.2126, 95%CI=-0.2077 to 0.6329, p=0.5565
Kye-shuan vs Lastochka: Diff=-0.1330, 95%CI=-0.5533 to 0.2873, p=0.8869
Czi-ti-4 vs Lastochka: Diff=-0.3456, 95%CI=-0.7659 to 0.0747, p=0.1327

Dr-TP1, Drought, Time-point 1:

ANOVA Table...

Source of Variation	Sum of Squares	d.f.	Variance	F	p
Between Groups:	10.1238	5	2.0248	10.0822	0.0006
Within Groups:	2.4099	12	0.2008		
Total:	12.5336	17			

Post-hoc tests...

```
Tukey HSD Post-hoc Test...
Vilana vs Zen: Diff=-0.2249, 95%CI=-1.4540 to 1.0041, p=0.9878
Vilana vs Sponsor: Diff=-1.3013, 95%CI=-2.5304 to -0.0723, p=0.0359
Vilana vs Kye-shuan: Diff=-1.8020, 95%CI=-3.0311 to -0.5730, p=0.0037
Vilana vs Czi-ti-4: Diff=-1.5785, 95%CI=-2.8076 to -0.3495, p=0.0100
Vilana vs Lastochka: Diff=-1.9024, 95%CI=-3.1314 to -0.6733, p=0.0024
Zen vs Sponsor: Diff=-1.0764, 95%CI=-2.3054 to 0.1527, p=0.0995
Zen vs Kye-shuan: Diff=-1.5771, 95%CI=-2.8062 to -0.3481, p=0.0101
Zen vs Czi-ti-4: Diff=-1.3536, 95%CI=-2.5826 to -0.1245, p=0.0282
Zen vs Lastochka: Diff=-1.6774, 95%CI=-2.9065 to -0.4484, p=0.0064
```

Tukey HSD Post-hoc Test:

Vilana vs Zen: Diff=-0.2249, 95%CI=-1.4540 to 1.0041, p=0.9878
Vilana vs Sponsor: Diff=-1.3013, 95%CI=-2.5304 to -0.0723, p=0.0359
Vilana vs Kye-shuan: Diff=-1.8020, 95%CI=-3.0311 to -0.5730, p=0.0037
Vilana vs Czi-ti-4: Diff=-1.5785, 95%CI=-2.8076 to -0.3495, p=0.0100
Vilana vs Lastochka: Diff=-1.9024, 95%CI=-3.1314 to -0.6733, p=0.0024
Zen vs Sponsor: Diff=-1.0764, 95%CI=-2.3054 to 0.1527, p=0.0995
Zen vs Kye-shuan: Diff=-1.5771, 95%CI=-2.8062 to -0.3481, p=0.0101
Zen vs Czi-ti-4: Diff=-1.3536, 95%CI=-2.5826 to -0.1245, p=0.0282
Zen vs Lastochka: Diff=-1.6774, 95%CI=-2.9065 to -0.4484, p=0.0064
Sponsor vs Kye-shuan: Diff=-0.5007, 95%CI=-1.7298 to 0.7283, p=0.7439
Sponsor vs Czi-ti-4: Diff=-0.2772, 95%CI=-1.5063 to 0.9518, p=0.9698
Sponsor vs Lastochka: Diff=-0.6011, 95%CI=-1.8301 to 0.6280, p=0.5889
Kye-shuan vs Czi-ti-4: Diff=0.2235, 95%CI=-1.0055 to 1.4526, p=0.9881
Kye-shuan vs Lastochka: Diff=-0.1003, 95%CI=-1.3294 to 1.1287, p=0.9997
Czi-ti-4 vs Lastochka: Diff=-0.3238, 95%CI=-1.5529 to 0.9052, p=0.9431

Dr-TP2, Drought, Time-point 2:

ANOVA Table...

Source of Variation	Sum of Squares	d.f.	Variance	F	p
Between Groups:	53.5078	5	10.7016	24.9065	0.0000
Within Groups:	5.1560	12	0.4297		
Total:	58.6638	17			

Post-hoc tests...

```
Tukey HSD Post-hoc Test...
Vilana vs Zen: Diff=1.4821, 95%CI=-0.3156 to 3.2799, p=0.1312
Vilana vs Sponsor: Diff=-1.3419, 95%CI=-3.1397 to 0.4558, p=0.1963
Vilana vs Kye-shuan: Diff=-2.8907, 95%CI=-4.6885 to -1.0930, p=0.0017
Vilana vs Czi-ti-4: Diff=-3.2412, 95%CI=-5.0389 to -1.4434, p=0.0006
Vilana vs Lastochka: Diff=-2.8073, 95%CI=-4.6050 to -1.0095, p=0.0022
Zen vs Sponsor: Diff=-2.8240, 95%CI=-4.6218 to -1.0263, p=0.0021
Zen vs Kye-shuan: Diff=-4.3728, 95%CI=-6.1706 to -2.5751, p=0.0000
Zen vs Czi-ti-4: Diff=-4.7233, 95%CI=-6.5210 to -2.9255, p=0.0000
Zen vs Lastochka: Diff=-4.2894, 95%CI=-6.0871 to -2.4916, p=0.0000
```

Tukey HSD Post-hoc Test:

Vilana vs Zen: Diff=1.4821, 95%CI=-0.3156 to 3.2799, p=0.1312
Vilana vs Sponsor: Diff=-1.3419, 95%CI=-3.1397 to 0.4558, p=0.1963
Vilana vs Kye-shuan: Diff=-2.8907, 95%CI=-4.6885 to -1.0930, p=0.0017
Vilana vs Czi-ti-4: Diff=-3.2412, 95%CI=-5.0389 to -1.4434, p=0.0006
Vilana vs Lastochka: Diff=-2.8073, 95%CI=-4.6050 to -1.0095, p=0.0022
Zen vs Sponsor: Diff=-2.8240, 95%CI=-4.6218 to -1.0263, p=0.0021
Zen vs Kye-shuan: Diff=-4.3728, 95%CI=-6.1706 to -2.5751, p=0.0000
Zen vs Czi-ti-4: Diff=-4.7233, 95%CI=-6.5210 to -2.9255, p=0.0000
Zen vs Lastochka: Diff=-4.2894, 95%CI=-6.0871 to -2.4916, p=0.0000
Sponsor vs Kye-shuan: Diff=-1.5488, 95%CI=-3.3465 to 0.2490, p=0.1075
Sponsor vs Czi-ti-4: Diff=-1.8992, 95%CI=-3.6970 to -0.1015, p=0.0364
Sponsor vs Lastochka: Diff=-1.4653, 95%CI=-3.2631 to 0.3324, p=0.1378
Kye-shuan vs Czi-ti-4: Diff=-0.3504, 95%CI=-2.1482 to 1.4473, p=0.9838
Kye-shuan vs Lastochka: Diff=0.0835, 95%CI=-1.7143 to 1.8812, p=1.0000
Czi-ti-4 vs Lastochka: Diff=0.4339, 95%CI=-1.3639 to 2.2316, p=0.9600

(C) Glyma.04G112700, *Transducin-1*

WW-TP1, Well-watered, Time-point 1:

ANOVA Table...

Source of Variation	Sum of Squares	d.f.	Variance	F	p
Between Groups:	0.1520	5	0.0304	0.1812	0.9644
Within Groups:	2.0134	12	0.1678		
Total:	2.1655	17			

Post-hoc tests...

```
Tukey HSD Post-hoc Test...
Vilana vs Zen: Diff=-0.1903, 95%CI=-1.3137 to 0.9331, p=0.9913
Vilana vs Sponsor: Diff=-0.0104, 95%CI=-1.1339 to 1.1130, p=1.0000
Vilana vs Kye-shuan: Diff=-0.1729, 95%CI=-1.2963 to 0.9505, p=0.9944
Vilana vs Czi-ti-4: Diff=0.0413, 95%CI=-1.0821 to 1.1647, p=1.0000
Vilana vs Lastochka: Diff=-0.1427, 95%CI=-1.2661 to 0.9807, p=0.9977
Zen vs Sponsor: Diff=0.1799, 95%CI=-0.9435 to 1.3033, p=0.9933
Zen vs Kye-shuan: Diff=0.0174, 95%CI=-1.1060 to 1.1408, p=1.0000
Zen vs Czi-ti-4: Diff=0.2316, 95%CI=-0.8918 to 1.3550, p=0.9794
Zen vs Lastochka: Diff=0.0476, 95%CI=-1.0758 to 1.1710, p=1.0000
```

Tukey HSD Post-hoc Test:

Vilana vs Zen: Diff=-0.1903, 95%CI=-1.3137 to 0.9331, p=0.9913
Vilana vs Sponsor: Diff=-0.0104, 95%CI=-1.1339 to 1.1130, p=1.0000
Vilana vs Kye-shuan: Diff=-0.1729, 95%CI=-1.2963 to 0.9505, p=0.9944
Vilana vs Czi-ti-4: Diff=0.0413, 95%CI=-1.0821 to 1.1647, p=1.0000
Vilana vs Lastochka: Diff=-0.1427, 95%CI=-1.2661 to 0.9807, p=0.9977
Zen vs Sponsor: Diff=0.1799, 95%CI=-0.9435 to 1.3033, p=0.9933
Zen vs Kye-shuan: Diff=0.0174, 95%CI=-1.1060 to 1.1408, p=1.0000
Zen vs Czi-ti-4: Diff=0.2316, 95%CI=-0.8918 to 1.3550, p=0.9794
Zen vs Lastochka: Diff=0.0476, 95%CI=-1.0758 to 1.1710, p=1.0000
Sponsor vs Kye-shuan: Diff=-0.1625, 95%CI=-1.2859 to 0.9610, p=0.9958
Sponsor vs Czi-ti-4: Diff=0.0517, 95%CI=-1.0717 to 1.1752, p=1.0000
Sponsor vs Lastochka: Diff=-0.1323, 95%CI=-1.2557 to 0.9911, p=0.9984
Kye-shuan vs Czi-ti-4: Diff=0.2142, 95%CI=-0.9092 to 1.3376, p=0.9853
Kye-shuan vs Lastochka: Diff=0.0302, 95%CI=-1.0932 to 1.1536, p=1.0000
Czi-ti-4 vs Lastochka: Diff=-0.1840, 95%CI=-1.3074 to 0.9394, p=0.9926

WW-TP2, Well-watered, Time-point 2:

ANOVA Table...

Source of Variation	Sum of Squares	d.f.	Variance	F	p
Between Groups:	0.0518	5	0.0104	0.0620	0.9968
Within Groups:	2.0056	12	0.1671		
Total:	2.0574	17			

Post-hoc tests...

```
Tukey HSD Post-hoc Test...
Vilana vs Zen: Diff=0.1689, 95%CI=-0.9524 to 1.2901, p=0.9949
Vilana vs Sponsor: Diff=0.0453, 95%CI=-1.0759 to 1.1665, p=1.0000
Vilana vs Kye-shuan: Diff=0.0916, 95%CI=-1.0296 to 1.2128, p=0.9997
Vilana vs Czi-ti-4: Diff=0.0547, 95%CI=-1.0665 to 1.1760, p=1.0000
Vilana vs Lastochka: Diff=0.1119, 95%CI=-1.0094 to 1.2331, p=0.9993
Zen vs Sponsor: Diff=-0.1235, 95%CI=-1.2448 to 0.9977, p=0.9988
Zen vs Kye-shuan: Diff=-0.0773, 95%CI=-1.1985 to 1.0440, p=0.9999
Zen vs Czi-ti-4: Diff=-0.1141, 95%CI=-1.2354 to 1.0071, p=0.9992
Zen vs Lastochka: Diff=-0.0570, 95%CI=-1.1782 to 1.0642, p=1.0000
```

Tukey HSD Post-hoc Test:

Vilana vs Zen: Diff=0.1689, 95%CI=-0.9524 to 1.2901, p=0.9949
Vilana vs Sponsor: Diff=0.0453, 95%CI=-1.0759 to 1.1665, p=1.0000
Vilana vs Kye-shuan: Diff=0.0916, 95%CI=-1.0296 to 1.2128, p=0.9997
Vilana vs Czi-ti-4: Diff=0.0547, 95%CI=-1.0665 to 1.1760, p=1.0000
Vilana vs Lastochka: Diff=0.1119, 95%CI=-1.0094 to 1.2331, p=0.9993
Zen vs Sponsor: Diff=-0.1235, 95%CI=-1.2448 to 0.9977, p=0.9988
Zen vs Kye-shuan: Diff=-0.0773, 95%CI=-1.1985 to 1.0440, p=0.9999
Zen vs Czi-ti-4: Diff=-0.1141, 95%CI=-1.2354 to 1.0071, p=0.9992
Zen vs Lastochka: Diff=-0.0570, 95%CI=-1.1782 to 1.0642, p=1.0000
Sponsor vs Kye-shuan: Diff=0.0463, 95%CI=-1.0749 to 1.1675, p=1.0000
Sponsor vs Czi-ti-4: Diff=0.0094, 95%CI=-1.1118 to 1.1306, p=1.0000
Sponsor vs Lastochka: Diff=0.0665, 95%CI=-1.0547 to 1.1878, p=0.9999
Kye-shuan vs Czi-ti-4: Diff=-0.0369, 95%CI=-1.1581 to 1.0844, p=1.0000
Kye-shuan vs Lastochka: Diff=0.0203, 95%CI=-1.1010 to 1.1415, p=1.0000
Czi-ti-4 vs Lastochka: Diff=0.0571, 95%CI=-1.0641 to 1.1784, p=1.0000

Dr-TP1, Drought, Time-point 1:

ANOVA Table...

Source of Variation	Sum of Squares	d.f.	Variance	F	p
Between Groups:	1.2378	5	0.2476	0.6426	0.6722
Within Groups:	4.6230	12	0.3852		
Total:	5.8607	17			

Post-hoc tests...

Tukey HSD Post-hoc Test...
Vilana vs Zen: Diff=-0.5884, 95%CI=-2.2906 to 1.1139, p=0.8465
Vilana vs Sponsor: Diff=-0.4445, 95%CI=-2.1468 to 1.2578, p=0.9451
Vilana vs Kye-shuan: Diff=0.0530, 95%CI=-1.6493 to 1.7553, p=1.0000
Vilana vs Czi-ti-4: Diff=-0.0103, 95%CI=-1.7126 to 1.6919, p=1.0012
Vilana vs Lastochka: Diff=-0.4708, 95%CI=-2.1731 to 1.2314, p=0.9312
Zen vs Sponsor: Diff=0.1439, 95%CI=-1.5584 to 1.8461, p=0.9997
Zen vs Kye-shuan: Diff=0.6414, 95%CI=-1.0609 to 2.3436, p=0.7973
Zen vs Czi-ti-4: Diff=0.5780, 95%CI=-1.1243 to 2.2803, p=0.8554
Zen vs Lastochka: Diff=0.1175, 95%CI=-1.5848 to 1.8198, p=0.9999

Tukey HSD Post-hoc Test:

Vilana vs Zen: Diff=-0.5884, 95%CI=-2.2906 to 1.1139, p=0.8465
Vilana vs Sponsor: Diff=-0.4445, 95%CI=-2.1468 to 1.2578, p=0.9451
Vilana vs Kye-shuan: Diff=0.0530, 95%CI=-1.6493 to 1.7553, p=1.0000
Vilana vs Czi-ti-4: Diff=-0.0103, 95%CI=-1.7126 to 1.6919, p=1.0012
Vilana vs Lastochka: Diff=-0.4708, 95%CI=-2.1731 to 1.2314, p=0.9312
Zen vs Sponsor: Diff=0.1439, 95%CI=-1.5584 to 1.8461, p=0.9997
Zen vs Kye-shuan: Diff=0.6414, 95%CI=-1.0609 to 2.3436, p=0.7973
Zen vs Czi-ti-4: Diff=0.5780, 95%CI=-1.1243 to 2.2803, p=0.8554
Zen vs Lastochka: Diff=0.1175, 95%CI=-1.5848 to 1.8198, p=0.9999
Sponsor vs Kye-shuan: Diff=0.4975, 95%CI=-1.2048 to 2.1998, p=0.9153
Sponsor vs Czi-ti-4: Diff=0.4342, 95%CI=-1.2681 to 2.1365, p=0.9500
Sponsor vs Lastochka: Diff=-0.0263, 95%CI=-1.7286 to 1.6760, p=1.0000
Kye-shuan vs Czi-ti-4: Diff=-0.0633, 95%CI=-1.7656 to 1.6389, p=1.0000
Kye-shuan vs Lastochka: Diff=-0.5238, 95%CI=-2.2261 to 1.1784, p=0.8976
Czi-ti-4 vs Lastochka: Diff=-0.4605, 95%CI=-2.1628 to 1.2418, p=0.9369

Dr-TP2, Drought, Time-point 2:

ANOVA Table...

Source of Variation	Sum of Squares	d.f.	Variance	F	p
Between Groups:	35.3851	5	7.0770	4.1801	0.0197
Within Groups:	20.3161	12	1.6930		
Total:	55.7012	17			

Post-hoc tests...

Tukey HSD Post-hoc Test...
Vilana vs Zen: Diff=0.8772, 95%CI=-2.6914 to 4.4457, p=0.9569
Vilana vs Sponsor: Diff=0.3644, 95%CI=-3.2041 to 3.9330, p=0.9992
Vilana vs Kye-shuan: Diff=2.8928, 95%CI=-0.6757 to 6.4614, p=0.1411
Vilana vs Czi-ti-4: Diff=2.6889, 95%CI=-0.8796 to 6.2575, p=0.1895
Vilana vs Lastochka: Diff=3.7156, 95%CI=0.1470 to 7.2841, p=0.0396
Zen vs Sponsor: Diff=-0.5127, 95%CI=-4.0813 to 3.0558, p=0.9959
Zen vs Kye-shuan: Diff=2.0156, 95%CI=-1.5529 to 5.5842, p=0.4477
Zen vs Czi-ti-4: Diff=1.8118, 95%CI=-1.7568 to 5.3803, p=0.5531
Zen vs Lastochka: Diff=2.8384, 95%CI=-0.7301 to 6.4070, p=0.1528

Tukey HSD Post-hoc Test:

Vilana vs Zen: Diff=0.8772, 95%CI=-2.6914 to 4.4457, p=0.9569
Vilana vs Sponsor: Diff=0.3644, 95%CI=-3.2041 to 3.9330, p=0.9992
Vilana vs Kye-shuan: Diff=2.8928, 95%CI=-0.6757 to 6.4614, p=0.1411
Vilana vs Czi-ti-4: Diff=2.6889, 95%CI=-0.8796 to 6.2575, p=0.1895
Vilana vs Lastochka: Diff=3.7156, 95%CI=0.1470 to 7.2841, p=0.0396
Zen vs Sponsor: Diff=-0.5127, 95%CI=-4.0813 to 3.0558, p=0.9959
Zen vs Kye-shuan: Diff=2.0156, 95%CI=-1.5529 to 5.5842, p=0.4477
Zen vs Czi-ti-4: Diff=1.8118, 95%CI=-1.7568 to 5.3803, p=0.5531
Zen vs Lastochka: Diff=2.8384, 95%CI=-0.7301 to 6.4070, p=0.1528
Sponsor vs Kye-shuan: Diff=2.5284, 95%CI=-1.0402 to 6.0969, p=0.2368
Sponsor vs Czi-ti-4: Diff=2.3245, 95%CI=-1.2440 to 5.8931, p=0.3098
Sponsor vs Lastochka: Diff=3.3512, 95%CI=-0.2174 to 6.9197, p=0.0703
Kye-shuan vs Czi-ti-4: Diff=-0.2039, 95%CI=-3.7724 to 3.3647, p=0.9999
Kye-shuan vs Lastochka: Diff=0.8228, 95%CI=-2.7458 to 4.3913, p=0.9669
Czi-ti-4 vs Lastochka: Diff=1.0266, 95%CI=-2.5419 to 4.5952, p=0.9201

(D) Glyma.17G093400, *Transducin-2*

WW-TP1, Well-watered, Time-point 1:

ANOVA Table...

Source of Variation	Sum of Squares	d.f.	Variance	F	p
Between Groups:	0.1271	5	0.0254	0.1463	0.9774
Within Groups:	2.0849	12	0.1737		
Total:	2.2120	17			

Post-hoc tests...

```
Tukey HSD Post-hoc Test...
Vilana vs Zen: Diff=0.0420, 95%CI=-1.1012 to 1.1851, p=1.0000
Vilana vs Sponsor: Diff=-0.2048, 95%CI=-1.3480 to 0.9384, p=0.9889
Vilana vs Kye-shuan: Diff=-0.0669, 95%CI=-1.2101 to 1.0762, p=0.9999
Vilana vs Czi-ti-4: Diff=-0.1325, 95%CI=-1.2757 to 1.0107, p=0.9985
Vilana vs Lastochka: Diff=-0.0138, 95%CI=-1.1570 to 1.1294, p=1.0000
Zen vs Sponsor: Diff=-0.2468, 95%CI=-1.3900 to 0.8964, p=0.9749
Zen vs Kye-shuan: Diff=-0.1089, 95%CI=-1.2521 to 1.0343, p=0.9994
Zen vs Czi-ti-4: Diff=-0.1744, 95%CI=-1.3176 to 0.9688, p=0.9946
Zen vs Lastochka: Diff=-0.0558, 95%CI=-1.1990 to 1.0874, p=1.0000
```

Tukey HSD Post-hoc Test:

Vilana vs Zen: Diff=0.0420, 95%CI=-1.1012 to 1.1851, p=1.0000
Vilana vs Sponsor: Diff=-0.2048, 95%CI=-1.3480 to 0.9384, p=0.9889
Vilana vs Kye-shuan: Diff=-0.0669, 95%CI=-1.2101 to 1.0762, p=0.9999
Vilana vs Czi-ti-4: Diff=-0.1325, 95%CI=-1.2757 to 1.0107, p=0.9985
Vilana vs Lastochka: Diff=-0.0138, 95%CI=-1.1570 to 1.1294, p=1.0000
Zen vs Sponsor: Diff=-0.2468, 95%CI=-1.3900 to 0.8964, p=0.9749
Zen vs Kye-shuan: Diff=-0.1089, 95%CI=-1.2521 to 1.0343, p=0.9994
Zen vs Czi-ti-4: Diff=-0.1744, 95%CI=-1.3176 to 0.9688, p=0.9946
Zen vs Lastochka: Diff=-0.0558, 95%CI=-1.1990 to 1.0874, p=1.0000
Sponsor vs Kye-shuan: Diff=0.1379, 95%CI=-1.0053 to 1.2811, p=0.9982
Sponsor vs Czi-ti-4: Diff=0.0723, 95%CI=-1.0709 to 1.2155, p=0.9999
Sponsor vs Lastochka: Diff=0.1910, 95%CI=-0.9522 to 1.3342, p=0.9919
Kye-shuan vs Czi-ti-4: Diff=-0.0655, 95%CI=-1.2087 to 1.0776, p=0.9999
Kye-shuan vs Lastochka: Diff=0.0531, 95%CI=-1.0901 to 1.1963, p=1.0000
Czi-ti-4 vs Lastochka: Diff=0.1187, 95%CI=-1.0245 to 1.2618, p=0.9991

WW-TP2, Well-watered, Time-point 2:

ANOVA Table...

Source of Variation	Sum of Squares	d.f.	Variance	F	p
Between Groups:	0.2727	5	0.0545	0.3872	0.8482
Within Groups:	1.6902	12	0.1408		
Total:	1.9628	17			

Post-hoc tests...

```
Tukey HSD Post-hoc Test...
Vilana vs Zen: Diff=-0.1100, 95%CI=-1.1393 to 0.9193, p=0.9990
Vilana vs Sponsor: Diff=-0.0893, 95%CI=-1.1186 to 0.9400, p=0.9996
Vilana vs Kye-shuan: Diff=0.2479, 95%CI=-0.7814 to 1.2772, p=0.9603
Vilana vs Czi-ti-4: Diff=-0.0961, 95%CI=-1.1254 to 0.9332, p=0.9995
Vilana vs Lastochka: Diff=-0.0357, 95%CI=-1.0650 to 0.9936, p=1.0000
Zen vs Sponsor: Diff=0.0207, 95%CI=-1.0086 to 1.0500, p=1.0000
Zen vs Kye-shuan: Diff=0.3579, 95%CI=-0.6714 to 1.3872, p=0.8434
Zen vs Czi-ti-4: Diff=0.0139, 95%CI=-1.0154 to 1.0432, p=1.0000
Zen vs Lastochka: Diff=0.0743, 95%CI=-0.9550 to 1.1036, p=0.9998
```

Tukey HSD Post-hoc Test:

Vilana vs Zen: Diff=-0.1100, 95%CI=-1.1393 to 0.9193, p=0.9990
Vilana vs Sponsor: Diff=-0.0893, 95%CI=-1.1186 to 0.9400, p=0.9996
Vilana vs Kye-shuan: Diff=0.2479, 95%CI=-0.7814 to 1.2772, p=0.9603
Vilana vs Czi-ti-4: Diff=-0.0961, 95%CI=-1.1254 to 0.9332, p=0.9995
Vilana vs Lastochka: Diff=-0.0357, 95%CI=-1.0650 to 0.9936, p=1.0000
Zen vs Sponsor: Diff=0.0207, 95%CI=-1.0086 to 1.0500, p=1.0000
Zen vs Kye-shuan: Diff=0.3579, 95%CI=-0.6714 to 1.3872, p=0.8434
Zen vs Czi-ti-4: Diff=0.0139, 95%CI=-1.0154 to 1.0432, p=1.0000
Zen vs Lastochka: Diff=0.0743, 95%CI=-0.9550 to 1.1036, p=0.9998
Sponsor vs Kye-shuan: Diff=0.3372, 95%CI=-0.6921 to 1.3665, p=0.8721
Sponsor vs Czi-ti-4: Diff=-0.0068, 95%CI=-1.0361 to 1.0224, p=1.0013
Sponsor vs Lastochka: Diff=0.0536, 95%CI=-0.9757 to 1.0829, p=1.0000
Kye-shuan vs Czi-ti-4: Diff=-0.3440, 95%CI=-1.3733 to 0.6852, p=0.8629
Kye-shuan vs Lastochka: Diff=-0.2836, 95%CI=-1.3129 to 0.7457, p=0.9322
Czi-ti-4 vs Lastochka: Diff=0.0604, 95%CI=-0.9689 to 1.0897, p=0.9999

Dr-TP1, Drought, Time-point 1:

ANOVA Table...

Source of Variation	Sum of Squares	d.f.	Variance	F	p
Between Groups:	2.5884	5	0.5177	2.0480	0.1434
Within Groups:	3.0333	12	0.2528		
Total:	5.6216	17			

Post-hoc tests...

```
Tukey HSD Post-hoc Test...
Vilana vs Zen: Diff=-0.1228, 95%CI=-1.5017 to 1.2561, p=0.9996
Vilana vs Sponsor: Diff=-0.0191, 95%CI=-1.3979 to 1.3598, p=1.0000
Vilana vs Kye-shuan: Diff=0.9184, 95%CI=-0.4604 to 2.2973, p=0.2896
Vilana vs Czi-ti-4: Diff=0.4686, 95%CI=-0.9102 to 1.8475, p=0.8550
Vilana vs Lastochka: Diff=-0.0873, 95%CI=-1.4662 to 1.2916, p=0.9999
Zen vs Sponsor: Diff=0.1038, 95%CI=-1.2751 to 1.4826, p=0.9998
Zen vs Kye-shuan: Diff=1.0413, 95%CI=-0.3376 to 2.4201, p=0.1879
Zen vs Czi-ti-4: Diff=0.5915, 95%CI=-0.7874 to 1.9703, p=0.7041
Zen vs Lastochka: Diff=0.0355, 95%CI=-1.3434 to 1.4144, p=1.0000
```

Tukey HSD Post-hoc Test:

Vilana vs Zen: Diff=-0.1228, 95%CI=-1.5017 to 1.2561, p=0.9996
Vilana vs Sponsor: Diff=-0.0191, 95%CI=-1.3979 to 1.3598, p=1.0000
Vilana vs Kye-shuan: Diff=0.9184, 95%CI=-0.4604 to 2.2973, p=0.2896
Vilana vs Czi-ti-4: Diff=0.4686, 95%CI=-0.9102 to 1.8475, p=0.8550
Vilana vs Lastochka: Diff=-0.0873, 95%CI=-1.4662 to 1.2916, p=0.9999
Zen vs Sponsor: Diff=0.1038, 95%CI=-1.2751 to 1.4826, p=0.9998
Zen vs Kye-shuan: Diff=1.0413, 95%CI=-0.3376 to 2.4201, p=0.1879
Zen vs Czi-ti-4: Diff=0.5915, 95%CI=-0.7874 to 1.9703, p=0.7041
Zen vs Lastochka: Diff=0.0355, 95%CI=-1.3434 to 1.4144, p=1.0000
Sponsor vs Kye-shuan: Diff=0.9375, 95%CI=-0.4414 to 2.3164, p=0.2715
Sponsor vs Czi-ti-4: Diff=0.4877, 95%CI=-0.8912 to 1.8666, p=0.8343
Sponsor vs Lastochka: Diff=-0.0682, 95%CI=-1.4471 to 1.3106, p=1.0000
Kye-shuan vs Czi-ti-4: Diff=-0.4498, 95%CI=-1.8287 to 0.9291, p=0.8740
Kye-shuan vs Lastochka: Diff=-1.0057, 95%CI=-2.3846 to 0.3731, p=0.2138
Czi-ti-4 vs Lastochka: Diff=-0.5559, 95%CI=-1.9348 to 0.8229, p=0.7515

Dr-TP2, Drought, Time-point 2:

ANOVA Table...

Source of Variation	Sum of Squares	d.f.	Variance	F	p
Between Groups:	9.9520	5	1.9904	1.8566	0.1762
Within Groups:	12.8652	12	1.0721		
Total:	22.8172	17			

Post-hoc tests...

```
Zen vs Kye-shuan: Diff=-0.6586, 95%CI=-3.4984 to 2.1811, p=0.9660
Zen vs Czi-ti-4: Diff=-1.1751, 95%CI=-4.0148 to 1.6647, p=0.7323
Zen vs Lastochka: Diff=-0.8651, 95%CI=-3.7048 to 1.9747, p=0.9013
Sponsor vs Kye-shuan: Diff=-1.7699, 95%CI=-4.6097 to 1.0698, p=0.3512
Sponsor vs Czi-ti-4: Diff=-2.2863, 95%CI=-5.1261 to 0.5534, p=0.1452
Sponsor vs Lastochka: Diff=-1.9763, 95%CI=-4.8161 to 0.8634, p=0.2516
Kye-shuan vs Czi-ti-4: Diff=-0.5164, 95%CI=-3.3562 to 2.3233, p=0.9881
Kye-shuan vs Lastochka: Diff=-0.2064, 95%CI=-3.0462 to 2.6333, p=0.9998
Czi-ti-4 vs Lastochka: Diff=0.3100, 95%CI=-2.5297 to 3.1497, p=0.9989
```

Tukey HSD Post-hoc Test:

Vilana vs Zen: Diff=0.1494, 95%CI=-2.6904 to 2.9891, p=1.0000
Vilana vs Sponsor: Diff=1.2606, 95%CI=-1.5791 to 4.1004, p=0.6758
Vilana vs Kye-shuan: Diff=-0.5093, 95%CI=-3.3490 to 2.3305, p=0.9888
Vilana vs Czi-ti-4: Diff=-1.0257, 95%CI=-3.8654 to 1.8141, p=0.8227
Vilana vs Lastochka: Diff=-0.7157, 95%CI=-3.5554 to 2.1241, p=0.9523
Zen vs Sponsor: Diff=1.1113, 95%CI=-1.7285 to 3.9510, p=0.7724
Zen vs Kye-shuan: Diff=-0.6586, 95%CI=-3.4984 to 2.1811, p=0.9660
Zen vs Czi-ti-4: Diff=-1.1751, 95%CI=-4.0148 to 1.6647, p=0.7323
Zen vs Lastochka: Diff=-0.8651, 95%CI=-3.7048 to 1.9747, p=0.9013
Sponsor vs Kye-shuan: Diff=-1.7699, 95%CI=-4.6097 to 1.0698, p=0.3512
Sponsor vs Czi-ti-4: Diff=-2.2863, 95%CI=-5.1261 to 0.5534, p=0.1452
Sponsor vs Lastochka: Diff=-1.9763, 95%CI=-4.8161 to 0.8634, p=0.2516
Kye-shuan vs Czi-ti-4: Diff=-0.5164, 95%CI=-3.3562 to 2.3233, p=0.9881
Kye-shuan vs Lastochka: Diff=-0.2064, 95%CI=-3.0462 to 2.6333, p=0.9998
Czi-ti-4 vs Lastochka: Diff=0.3100, 95%CI=-2.5297 to 3.1497, p=0.9989

(E) Glyma.15G156600, Pentatricopeptide repeat *(PPR)*

WW-TP1, Well-watered, Time-point 1:

ANOVA Table...

Source of Variation	Sum of Squares	d.f.	Variance	F	p
Between Groups:	0.1882	5	0.0376	0.1201	0.9853
Within Groups:	3.7614	12	0.3134		
Total:	3.9496	17			

Post-hoc tests...

```
Vilana vs Zen: Diff=0.1075, 95%CI=-1.4280 to 1.6429, p=0.9999
Vilana vs Sponsor: Diff=-0.2114, 95%CI=-1.7469 to 1.3241, p=0.9967
Vilana vs Kye-shuan: Diff=-0.0458, 95%CI=-1.5813 to 1.4897, p=1.0000
Vilana vs Czi-ti-4: Diff=0.0740, 95%CI=-1.4615 to 1.6094, p=1.0000
Vilana vs Lastochka: Diff=-0.0059, 95%CI=-1.5414 to 1.5296, p=1.0383
Zen vs Sponsor: Diff=-0.3189, 95%CI=-1.8544 to 1.2166, p=0.9787
Zen vs Kye-shuan: Diff=-0.1533, 95%CI=-1.6888 to 1.3822, p=0.9993
Zen vs Czi-ti-4: Diff=-0.0335, 95%CI=-1.5690 to 1.5020, p=1.0000
Zen vs Lastochka: Diff=-0.1134, 95%CI=-1.6488 to 1.4221, p=0.9998
Sponsor vs Kye-shuan: Diff=0.1656, 95%CI=-1.3699 to 1.7011, p=0.9989
```

Tukey HSD Post-hoc Test:

Vilana vs Zen: Diff=0.1075, 95%CI=-1.4280 to 1.6429, p=0.9999
Vilana vs Sponsor: Diff=-0.2114, 95%CI=-1.7469 to 1.3241, p=0.9967
Vilana vs Kye-shuan: Diff=-0.0458, 95%CI=-1.5813 to 1.4897, p=1.0000
Vilana vs Czi-ti-4: Diff=0.0740, 95%CI=-1.4615 to 1.6094, p=1.0000
Vilana vs Lastochka: Diff=-0.0059, 95%CI=-1.5414 to 1.5296, p=1.0383
Zen vs Sponsor: Diff=-0.3189, 95%CI=-1.8544 to 1.2166, p=0.9787
Zen vs Kye-shuan: Diff=-0.1533, 95%CI=-1.6888 to 1.3822, p=0.9993
Zen vs Czi-ti-4: Diff=-0.0335, 95%CI=-1.5690 to 1.5020, p=1.0000
Zen vs Lastochka: Diff=-0.1134, 95%CI=-1.6488 to 1.4221, p=0.9998
Sponsor vs Kye-shuan: Diff=0.1656, 95%CI=-1.3699 to 1.7011, p=0.9989
Sponsor vs Czi-ti-4: Diff=0.2854, 95%CI=-1.2501 to 1.8208, p=0.9869
Sponsor vs Lastochka: Diff=0.2055, 95%CI=-1.3300 to 1.7410, p=0.9971
Kye-shuan vs Czi-ti-4: Diff=0.1198, 95%CI=-1.4157 to 1.6553, p=0.9998
Kye-shuan vs Lastochka: Diff=0.0399, 95%CI=-1.4956 to 1.5754, p=1.0000
Czi-ti-4 vs Lastochka: Diff=-0.0799, 95%CI=-1.6153 to 1.4556, p=1.0000

WW-TP2, Well-watered, Time-point 2:

ANOVA Table...

Source of Variation	Sum of Squares	d.f.	Variance	F	p
Between Groups:	0.2428	5	0.0486	0.3712	0.8588
Within Groups:	1.5699	12	0.1308		
Total:	1.8127	17			

Post-hoc tests...

```
Vilana vs Zen: Diff=-0.1023, 95%CI=-1.0943 to 0.8897, p=0.9991
Vilana vs Sponsor: Diff=-0.1421, 95%CI=-1.1341 to 0.8499, p=0.9960
Vilana vs Kye-shuan: Diff=0.1275, 95%CI=-0.8645 to 1.1195, p=0.9976
Vilana vs Czi-ti-4: Diff=-0.2031, 95%CI=-1.1950 to 0.7889, p=0.9800
Vilana vs Lastochka: Diff=0.0621, 95%CI=-0.9298 to 1.0541, p=0.9999
Zen vs Sponsor: Diff=-0.0398, 95%CI=-1.0318 to 0.9522, p=1.0000
Zen vs Kye-shuan: Diff=0.2298, 95%CI=-0.7622 to 1.2218, p=0.9662
Zen vs Czi-ti-4: Diff=-0.1007, 95%CI=-1.0927 to 0.8912, p=0.9992
Zen vs Lastochka: Diff=0.1645, 95%CI=-0.8275 to 1.1564, p=0.9921
Sponsor vs Kye-shuan: Diff=0.2696, 95%CI=-0.7224 to 1.2616, p=0.9357
```

Tukey HSD Post-hoc Test:

Vilana vs Zen: Diff=-0.1023, 95%CI=-1.0943 to 0.8897, p=0.9991
Vilana vs Sponsor: Diff=-0.1421, 95%CI=-1.1341 to 0.8499, p=0.9960
Vilana vs Kye-shuan: Diff=0.1275, 95%CI=-0.8645 to 1.1195, p=0.9976
Vilana vs Czi-ti-4: Diff=-0.2031, 95%CI=-1.1950 to 0.7889, p=0.9800
Vilana vs Lastochka: Diff=0.0621, 95%CI=-0.9298 to 1.0541, p=0.9999
Zen vs Sponsor: Diff=-0.0398, 95%CI=-1.0318 to 0.9522, p=1.0000
Zen vs Kye-shuan: Diff=0.2298, 95%CI=-0.7622 to 1.2218, p=0.9662
Zen vs Czi-ti-4: Diff=-0.1007, 95%CI=-1.0927 to 0.8912, p=0.9992
Zen vs Lastochka: Diff=0.1645, 95%CI=-0.8275 to 1.1564, p=0.9921
Sponsor vs Kye-shuan: Diff=0.2696, 95%CI=-0.7224 to 1.2616, p=0.9357
Sponsor vs Czi-ti-4: Diff=-0.0610, 95%CI=-1.0529 to 0.9310, p=0.9999
Sponsor vs Lastochka: Diff=0.2043, 95%CI=-0.7877 to 1.1962, p=0.9795
Kye-shuan vs Czi-ti-4: Diff=-0.3306, 95%CI=-1.3225 to 0.6614, p=0.8644
Kye-shuan vs Lastochka: Diff=-0.0654, 95%CI=-1.0573 to 0.9266, p=0.9999
Czi-ti-4 vs Lastochka: Diff=0.2652, 95%CI=-0.7268 to 1.2572, p=0.9397

Dr-TP1, Drought, Time-point 1:

ANOVA Table...

Source of Variation	Sum of Squares	d.f.	Variance	F	p
Between Groups:	12.2090	5	2.4418	6.0834	0.0050
Within Groups:	4.8167	12	0.4014		
Total:	17.0257	17			

Post-hoc tests...

```
Vilana vs Zen: Diff=-1.2705, 95%CI=-3.0081 to 0.4671, p=0.2119
Vilana vs Sponsor: Diff=-1.1777, 95%CI=-2.9153 to 0.5599, p=0.2742
Vilana vs Kye-shuan: Diff=-2.3405, 95%CI=-4.0781 to -0.6030, p=0.0070
Vilana vs Czi-ti-4: Diff=-1.9683, 95%CI=-3.7059 to -0.2307, p=0.0236
Vilana vs Lastochka: Diff=-2.3600, 95%CI=-4.0976 to -0.6224, p=0.0066
Zen vs Sponsor: Diff=0.0928, 95%CI=-1.6448 to 1.8304, p=1.0000
Zen vs Kye-shuan: Diff=-1.0700, 95%CI=-2.8076 to 0.6676, p=0.3627
Zen vs Czi-ti-4: Diff=-0.6978, 95%CI=-2.4354 to 1.0398, p=0.7543
Zen vs Lastochka: Diff=-1.0895, 95%CI=-2.8271 to 0.6481, p=0.3455
Sponsor vs Kye-shuan: Diff=-1.1628, 95%CI=-2.9004 to 0.5747, p=0.2854
```

Tukey HSD Post-hoc Test:

Vilana vs Zen: Diff=-1.2705, 95%CI=-3.0081 to 0.4671, p=0.2119
Vilana vs Sponsor: Diff=-1.1777, 95%CI=-2.9153 to 0.5599, p=0.2742
Vilana vs Kye-shuan: Diff=-2.3405, 95%CI=-4.0781 to -0.6030, p=0.0070
Vilana vs Czi-ti-4: Diff=-1.9683, 95%CI=-3.7059 to -0.2307, p=0.0236
Vilana vs Lastochka: Diff=-2.3600, 95%CI=-4.0976 to -0.6224, p=0.0066
Zen vs Sponsor: Diff=0.0928, 95%CI=-1.6448 to 1.8304, p=1.0000
Zen vs Kye-shuan: Diff=-1.0700, 95%CI=-2.8076 to 0.6676, p=0.3627
Zen vs Czi-ti-4: Diff=-0.6978, 95%CI=-2.4354 to 1.0398, p=0.7543
Zen vs Lastochka: Diff=-1.0895, 95%CI=-2.8271 to 0.6481, p=0.3455
Sponsor vs Kye-shuan: Diff=-1.1628, 95%CI=-2.9004 to 0.5747, p=0.2854
Sponsor vs Czi-ti-4: Diff=-0.7906, 95%CI=-2.5282 to 0.9470, p=0.6546
Sponsor vs Lastochka: Diff=-1.1823, 95%CI=-2.9199 to 0.5553, p=0.2709
Kye-shuan vs Czi-ti-4: Diff=0.3722, 95%CI=-1.3653 to 2.1098, p=0.9757
Kye-shuan vs Lastochka: Diff=-0.0195, 95%CI=-1.7570 to 1.7181, p=1.0000
Czi-ti-4 vs Lastochka: Diff=-0.3917, 95%CI=-2.1293 to 1.3459, p=0.9698

Dr-TP2, Drought, Time-point 2:

ANOVA Table...

Source of Variation	Sum of Squares	d.f.	Variance	F	p
Between Groups:	86.1902	5	17.2380	7.9524	0.0016
Within Groups:	26.0120	12	2.1677		
Total:	112.2021	17			

Post-hoc tests...

```
Vilana vs Zen: Diff=1.4429, 95%CI=-2.5950 to 5.4808, p=0.8287
Vilana vs Sponsor: Diff=-0.4230, 95%CI=-4.4609 to 3.6149, p=0.9991
Vilana vs Kye-shuan: Diff=-4.1061, 95%CI=-8.1440 to -0.0681, p=0.0455
Vilana vs Czi-ti-4: Diff=-3.7239, 95%CI=-7.7618 to 0.3140, p=0.0772
Vilana vs Lastochka: Diff=-3.8157, 95%CI=-7.8537 to 0.2222, p=0.0680
Zen vs Sponsor: Diff=-1.8659, 95%CI=-5.9039 to 2.1720, p=0.6409
Zen vs Kye-shuan: Diff=-5.5490, 95%CI=-9.5869 to -1.5111, p=0.0061
Zen vs Czi-ti-4: Diff=-5.1668, 95%CI=-9.2047 to -1.1289, p=0.0103
Zen vs Lastochka: Diff=-5.2587, 95%CI=-9.2966 to -1.2208, p=0.0090
Sponsor vs Kye-shuan: Diff=-3.6830, 95%CI=-7.7210 to 0.3549, p=0.0816
```

Tukey HSD Post-hoc Test:

Vilana vs Zen: Diff=1.4429, 95%CI=-2.5950 to 5.4808, p=0.8287
Vilana vs Sponsor: Diff=-0.4230, 95%CI=-4.4609 to 3.6149, p=0.9991
Vilana vs Kye-shuan: Diff=-4.1061, 95%CI=-8.1440 to -0.0681, p=0.0455
Vilana vs Czi-ti-4: Diff=-3.7239, 95%CI=-7.7618 to 0.3140, p=0.0772
Vilana vs Lastochka: Diff=-3.8157, 95%CI=-7.8537 to 0.2222, p=0.0680
Zen vs Sponsor: Diff=-1.8659, 95%CI=-5.9039 to 2.1720, p=0.6409
Zen vs Kye-shuan: Diff=-5.5490, 95%CI=-9.5869 to -1.5111, p=0.0061
Zen vs Czi-ti-4: Diff=-5.1668, 95%CI=-9.2047 to -1.1289, p=0.0103
Zen vs Lastochka: Diff=-5.2587, 95%CI=-9.2966 to -1.2208, p=0.0090
Sponsor vs Kye-shuan: Diff=-3.6830, 95%CI=-7.7210 to 0.3549, p=0.0816
Sponsor vs Czi-ti-4: Diff=-3.3009, 95%CI=-7.3388 to 0.7371, p=0.1361
Sponsor vs Lastochka: Diff=-3.3927, 95%CI=-7.4306 to 0.6452, p=0.1206
Kye-shuan vs Czi-ti-4: Diff=0.3822, 95%CI=-3.6557 to 4.4201, p=0.9994
Kye-shuan vs Lastochka: Diff=0.2903, 95%CI=-3.7476 to 4.3282, p=0.9998
Czi-ti-4 vs Lastochka: Diff=-0.0919, 95%CI=-4.1298 to 3.9461, p=1.0000

(F) Glyma.15G092400, ATP-binding cassette (*ABC*)

WW-TP1, Well-watered, Time-point 1:

ANOVA Table...

Source of Variation	Sum of Squares	d.f.	Variance	F	p
Between Groups:	0.1860	5	0.0372	0.3352	0.8820
Within Groups:	1.3315	12	0.1110		
Total:	1.5175	17			

Post-hoc tests...

Tukey HSD Post-hoc Test...
Vilana vs Zen: Diff=0.2067, 95%CI=-0.7069 to 1.1202, p=0.9694
Vilana vs Sponsor: Diff=0.3363, 95%CI=-0.5773 to 1.2498, p=0.8116
Vilana vs Kye-shuan: Diff=0.1560, 95%CI=-0.7576 to 1.0695, p=0.9910
Vilana vs Czi-ti-4: Diff=0.2453, 95%CI=-0.6682 to 1.1589, p=0.9387
Vilana vs Lastochka: Diff=0.1859, 95%CI=-0.7276 to 1.0995, p=0.9805
Zen vs Sponsor: Diff=0.1296, 95%CI=-0.7840 to 1.0431, p=0.9962
Zen vs Kye-shuan: Diff=-0.0507, 95%CI=-0.9643 to 0.8628, p=0.9999
Zen vs Czi-ti-4: Diff=0.0387, 95%CI=-0.8749 to 0.9522, p=1.0000
Zen vs Lastochka: Diff=-0.0208, 95%CI=-0.9343 to 0.8928, p=1.0000

Tukey HSD Post-hoc Test:

Vilana vs Zen: Diff=0.2067, 95%CI=-0.7069 to 1.1202, p=0.9694
Vilana vs Sponsor: Diff=0.3363, 95%CI=-0.5773 to 1.2498, p=0.8116
Vilana vs Kye-shuan: Diff=0.1560, 95%CI=-0.7576 to 1.0695, p=0.9910
Vilana vs Czi-ti-4: Diff=0.2453, 95%CI=-0.6682 to 1.1589, p=0.9387
Vilana vs Lastochka: Diff=0.1859, 95%CI=-0.7276 to 1.0995, p=0.9805
Zen vs Sponsor: Diff=0.1296, 95%CI=-0.7840 to 1.0431, p=0.9962
Zen vs Kye-shuan: Diff=-0.0507, 95%CI=-0.9643 to 0.8628, p=0.9999
Zen vs Czi-ti-4: Diff=0.0387, 95%CI=-0.8749 to 0.9522, p=1.0000
Zen vs Lastochka: Diff=-0.0208, 95%CI=-0.9343 to 0.8928, p=1.0000
Sponsor vs Kye-shuan: Diff=-0.1803, 95%CI=-1.0939 to 0.7332, p=0.9829
Sponsor vs Czi-ti-4: Diff=-0.0909, 95%CI=-1.0045 to 0.8226, p=0.9993
Sponsor vs Lastochka: Diff=-0.1503, 95%CI=-1.0639 to 0.7632, p=0.9924
Kye-shuan vs Czi-ti-4: Diff=0.0894, 95%CI=-0.8242 to 1.0030, p=0.9993
Kye-shuan vs Lastochka: Diff=0.0300, 95%CI=-0.8836 to 0.9435, p=1.0000
Czi-ti-4 vs Lastochka: Diff=-0.0594, 95%CI=-0.9730 to 0.8541, p=0.9999

WW-TP2, Well-watered, Time-point 2:

ANOVA Table...

Source of Variation	Sum of Squares	d.f.	Variance	F	p
Between Groups:	0.3961	5	0.0792	0.5857	0.7111
Within Groups:	1.6233	12	0.1353		
Total:	2.0194	17			

Post-hoc tests...

Vilana vs Zen: Diff=0.1196, 95%CI=-0.8891 to 1.1284, p=0.9984
Vilana vs Sponsor: Diff=0.2590, 95%CI=-0.7497 to 1.2677, p=0.9486
Vilana vs Kye-shuan: Diff=-0.0676, 95%CI=-1.0763 to 0.9412, p=0.9999
Vilana vs Czi-ti-4: Diff=0.0094, 95%CI=-0.9993 to 1.0181, p=1.0000
Vilana vs Lastochka: Diff=-0.2185, 95%CI=-1.2272 to 0.7902, p=0.9745
Zen vs Sponsor: Diff=0.1394, 95%CI=-0.8694 to 1.1481, p=0.9966
Zen vs Kye-shuan: Diff=-0.1872, 95%CI=-1.1959 to 0.8215, p=0.9870
Zen vs Czi-ti-4: Diff=-0.1102, 95%CI=-1.1189 to 0.8985, p=0.9989
Zen vs Lastochka: Diff=-0.3381, 95%CI=-1.3468 to 0.6706, p=0.8616
Sponsor vs Kye-shuan: Diff=-0.3265, 95%CI=-1.3353 to 0.6822, p=0.8773

Tukey HSD Post-hoc Test:

Vilana vs Zen: Diff=0.1196, 95%CI=-0.8891 to 1.1284, p=0.9984
Vilana vs Sponsor: Diff=0.2590, 95%CI=-0.7497 to 1.2677, p=0.9486
Vilana vs Kye-shuan: Diff=-0.0676, 95%CI=-1.0763 to 0.9412, p=0.9999
Vilana vs Czi-ti-4: Diff=0.0094, 95%CI=-0.9993 to 1.0181, p=1.0000
Vilana vs Lastochka: Diff=-0.2185, 95%CI=-1.2272 to 0.7902, p=0.9745
Zen vs Sponsor: Diff=0.1394, 95%CI=-0.8694 to 1.1481, p=0.9966
Zen vs Kye-shuan: Diff=-0.1872, 95%CI=-1.1959 to 0.8215, p=0.9870
Zen vs Czi-ti-4: Diff=-0.1102, 95%CI=-1.1189 to 0.8985, p=0.9989
Zen vs Lastochka: Diff=-0.3381, 95%CI=-1.3468 to 0.6706, p=0.8616
Sponsor vs Kye-shuan: Diff=-0.3265, 95%CI=-1.3353 to 0.6822, p=0.8773
Sponsor vs Czi-ti-4: Diff=-0.2496, 95%CI=-1.2583 to 0.7592, p=0.9557
Sponsor vs Lastochka: Diff=-0.4775, 95%CI=-1.4862 to 0.5312, p=0.6192
Kye-shuan vs Czi-ti-4: Diff=0.0770, 95%CI=-0.9318 to 1.0857, p=0.9998
Kye-shuan vs Lastochka: Diff=-0.1509, 95%CI=-1.1597 to 0.8578, p=0.9951
Czi-ti-4 vs Lastochka: Diff=-0.2279, 95%CI=-1.2366 to 0.7808, p=0.9695

Dr-TP1, Drought, Time-point 1:

ANOVA Table...

Source of Variation	Sum of Squares	d.f.	Variance	F	p
Between Groups:	17.5685	5	3.5137	14.6018	0.0001
Within Groups:	2.8876	12	0.2406		
Total:	20.4561	17			

Post-hoc tests...

```
Tukey HSD Post-hoc Test...
Vilana vs Zen: Diff=1.8238, 95%CI=0.4785 to 3.1692, p=0.0067
Vilana vs Sponsor: Diff=-0.5260, 95%CI=-1.8714 to 0.8194, p=0.7731
Vilana vs Kye-shuan: Diff=-0.1847, 95%CI=-1.5301 to 1.1606, p=0.9967
Vilana vs Czi-ti-4: Diff=-1.3279, 95%CI=-2.6733 to 0.0175, p=0.0538
Vilana vs Lastochka: Diff=-0.7898, 95%CI=-2.1352 to 0.5555, p=0.4094
Zen vs Sponsor: Diff=-2.3498, 95%CI=-3.6952 to -1.0045, p=0.0008
Zen vs Kye-shuan: Diff=-2.0086, 95%CI=-3.3539 to -0.6632, p=0.0032
Zen vs Czi-ti-4: Diff=-3.1518, 95%CI=-4.4971 to -1.8064, p=0.0001
Zen vs Lastochka: Diff=-2.6137, 95%CI=-3.9590 to -1.2683, p=0.0003
```

Tukey HSD Post-hoc Test:

Vilana vs Zen: Diff=1.8238, 95%CI=0.4785 to 3.1692, p=0.0067
Vilana vs Sponsor: Diff=-0.5260, 95%CI=-1.8714 to 0.8194, p=0.7731
Vilana vs Kye-shuan: Diff=-0.1847, 95%CI=-1.5301 to 1.1606, p=0.9967
Vilana vs Czi-ti-4: Diff=-1.3279, 95%CI=-2.6733 to 0.0175, p=0.0538
Vilana vs Lastochka: Diff=-0.7898, 95%CI=-2.1352 to 0.5555, p=0.4094
Zen vs Sponsor: Diff=-2.3498, 95%CI=-3.6952 to -1.0045, p=0.0008
Zen vs Kye-shuan: Diff=-2.0086, 95%CI=-3.3539 to -0.6632, p=0.0032
Zen vs Czi-ti-4: Diff=-3.1518, 95%CI=-4.4971 to -1.8064, p=0.0001
Zen vs Lastochka: Diff=-2.6137, 95%CI=-3.9590 to -1.2683, p=0.0003
Sponsor vs Kye-shuan: Diff=0.3413, 95%CI=-1.0041 to 1.6866, p=0.9510
Sponsor vs Czi-ti-4: Diff=-0.8019, 95%CI=-2.1473 to 0.5435, p=0.3944
Sponsor vs Lastochka: Diff=-0.2638, 95%CI=-1.6092 to 1.0815, p=0.9834
Kye-shuan vs Czi-ti-4: Diff=-1.1432, 95%CI=-2.4886 to 0.2022, p=0.1146
Kye-shuan vs Lastochka: Diff=-0.6051, 95%CI=-1.9505 to 0.7402, p=0.6646
Czi-ti-4 vs Lastochka: Diff=0.5381, 95%CI=-0.8073 to 1.8834, p=0.7573

Dr-TP2, Drought, Time-point 2:

ANOVA Table...

Source of Variation	Sum of Squares	d.f.	Variance	F	p
Between Groups:	21.5175	5	4.3035	8.0120	0.0016
Within Groups:	6.4456	12	0.5371		
Total:	27.9631	17			

Post-hoc tests...

```
Tukey HSD Post-hoc Test...
Vilana vs Zen: Diff=0.9331, 95%CI=-1.0769 to 2.9431, p=0.6368
Vilana vs Sponsor: Diff=-0.8947, 95%CI=-2.9047 to 1.1153, p=0.6735
Vilana vs Kye-shuan: Diff=-1.6675, 95%CI=-3.6775 to 0.3425, p=0.1276
Vilana vs Czi-ti-4: Diff=-2.1983, 95%CI=-4.2083 to -0.1883, p=0.0294
Vilana vs Lastochka: Diff=-1.7563, 95%CI=-3.7663 to 0.2538, p=0.1006
Zen vs Sponsor: Diff=-1.8278, 95%CI=-3.8378 to 0.1822, p=0.0829
Zen vs Kye-shuan: Diff=-2.6006, 95%CI=-4.6106 to -0.5906, p=0.0095
Zen vs Czi-ti-4: Diff=-3.1314, 95%CI=-5.1414 to -1.1214, p=0.0022
Zen vs Lastochka: Diff=-2.6894, 95%CI=-4.6994 to -0.6793, p=0.0074
```

Tukey HSD Post-hoc Test:

Vilana vs Zen: Diff=0.9331, 95%CI=-1.0769 to 2.9431, p=0.6368
Vilana vs Sponsor: Diff=-0.8947, 95%CI=-2.9047 to 1.1153, p=0.6735
Vilana vs Kye-shuan: Diff=-1.6675, 95%CI=-3.6775 to 0.3425, p=0.1276
Vilana vs Czi-ti-4: Diff=-2.1983, 95%CI=-4.2083 to -0.1883, p=0.0294
Vilana vs Lastochka: Diff=-1.7563, 95%CI=-3.7663 to 0.2538, p=0.1006
Zen vs Sponsor: Diff=-1.8278, 95%CI=-3.8378 to 0.1822, p=0.0829
Zen vs Kye-shuan: Diff=-2.6006, 95%CI=-4.6106 to -0.5906, p=0.0095
Zen vs Czi-ti-4: Diff=-3.1314, 95%CI=-5.1414 to -1.1214, p=0.0022
Zen vs Lastochka: Diff=-2.6894, 95%CI=-4.6994 to -0.6793, p=0.0074
Sponsor vs Kye-shuan: Diff=-0.7728, 95%CI=-2.7829 to 1.2372, p=0.7842
Sponsor vs Czi-ti-4: Diff=-1.3036, 95%CI=-3.3136 to 0.7064, p=0.3138
Sponsor vs Lastochka: Diff=-0.8616, 95%CI=-2.8716 to 1.1484, p=0.7047
Kye-shuan vs Czi-ti-4: Diff=-0.5308, 95%CI=-2.5408 to 1.4793, p=0.9426
Kye-shuan vs Lastochka: Diff=-0.0887, 95%CI=-2.0988 to 1.9213, p=1.0000
Czi-ti-4 vs Lastochka: Diff=0.4420, 95%CI=-1.5680 to 2.4521, p=0.9728

(G) Glyma.05G163000, Nitrate transporter (*NTR1.2*)

WW-TP1, Well-watered, Time-point 1:

ANOVA Table...

Source of Variation	Sum of Squares	d.f.	Variance	F	p
Between Groups:	0.2683	5	0.0537	0.4929	0.7757
Within Groups:	1.3067	12	0.1089		
Total:	1.5750	17			

Post-hoc tests...

Vilana vs Zen: Diff=0.0376, 95%CI=-0.8674 to 0.9426, p=1.0000
Vilana vs Sponsor: Diff=0.0582, 95%CI=-0.8469 to 0.9632, p=0.9999
Vilana vs Kye-shuan: Diff=0.3643, 95%CI=-0.5407 to 1.2693, p=0.7526
Vilana vs Czi-ti-4: Diff=0.1697, 95%CI=-0.7354 to 1.0747, p=0.9864
Vilana vs Lastochka: Diff=0.0717, 95%CI=-0.8334 to 0.9767, p=0.9998
Zen vs Sponsor: Diff=0.0205, 95%CI=-0.8845 to 0.9256, p=1.0000
Zen vs Kye-shuan: Diff=0.3267, 95%CI=-0.5783 to 1.2317, p=0.8230
Zen vs Czi-ti-4: Diff=0.1320, 95%CI=-0.7730 to 1.0371, p=0.9956
Zen vs Lastochka: Diff=0.0340, 95%CI=-0.8710 to 0.9391, p=1.0000
Sponsor vs Kye-shuan: Diff=0.3062, 95%CI=-0.5989 to 1.2112, p=0.8572

Tukey HSD Post-hoc Test:

Vilana vs Zen: Diff=0.0376, 95%CI=-0.8674 to 0.9426, p=1.0000
Vilana vs Sponsor: Diff=0.0582, 95%CI=-0.8469 to 0.9632, p=0.9999
Vilana vs Kye-shuan: Diff=0.3643, 95%CI=-0.5407 to 1.2693, p=0.7526
Vilana vs Czi-ti-4: Diff=0.1697, 95%CI=-0.7354 to 1.0747, p=0.9864
Vilana vs Lastochka: Diff=0.0717, 95%CI=-0.8334 to 0.9767, p=0.9998
Zen vs Sponsor: Diff=0.0205, 95%CI=-0.8845 to 0.9256, p=1.0000
Zen vs Kye-shuan: Diff=0.3267, 95%CI=-0.5783 to 1.2317, p=0.8230
Zen vs Czi-ti-4: Diff=0.1320, 95%CI=-0.7730 to 1.0371, p=0.9956
Zen vs Lastochka: Diff=0.0340, 95%CI=-0.8710 to 0.9391, p=1.0000
Sponsor vs Kye-shuan: Diff=0.3062, 95%CI=-0.5989 to 1.2112, p=0.8572
Sponsor vs Czi-ti-4: Diff=0.1115, 95%CI=-0.7935 to 1.0165, p=0.9980
Sponsor vs Lastochka: Diff=0.0135, 95%CI=-0.8915 to 0.9185, p=1.0000
Kye-shuan vs Czi-ti-4: Diff=-0.1946, 95%CI=-1.0997 to 0.7104, p=0.9753
Kye-shuan vs Lastochka: Diff=-0.2927, 95%CI=-1.1977 to 0.6124, p=0.8778
Czi-ti-4 vs Lastochka: Diff=-0.0980, 95%CI=-1.0030 to 0.8070, p=0.9989

WW-TP2, Well-watered, Time-point 2:

ANOVA Table...

Source of Variation	Sum of Squares	d.f.	Variance	F	p
Between Groups:	0.3767	5	0.0753	0.7257	0.6172
Within Groups:	1.2457	12	0.1038		
Total:	1.6224	17			

Post-hoc tests...

Tukey HSD Post-hoc Test...
Vilana vs Zen: Diff=-0.0225, 95%CI=-0.9062 to 0.8611, p=1.0000
Vilana vs Sponsor: Diff=0.2043, 95%CI=-0.6793 to 1.0880, p=0.9665
Vilana vs Kye-shuan: Diff=0.2948, 95%CI=-0.5888 to 1.1785, p=0.8638
Vilana vs Czi-ti-4: Diff=-0.1259, 95%CI=-1.0095 to 0.7578, p=0.9961
Vilana vs Lastochka: Diff=0.1488, 95%CI=-0.7348 to 1.0324, p=0.9916
Zen vs Sponsor: Diff=0.2268, 95%CI=-0.6568 to 1.1105, p=0.9487
Zen vs Kye-shuan: Diff=0.3174, 95%CI=-0.5663 to 1.2010, p=0.8259
Zen vs Czi-ti-4: Diff=-0.1033, 95%CI=-0.9870 to 0.7803, p=0.9985
Zen vs Lastochka: Diff=0.1713, 95%CI=-0.7123 to 1.0550, p=0.9842

Tukey HSD Post-hoc Test:

Vilana vs Zen: Diff=-0.0225, 95%CI=-0.9062 to 0.8611, p=1.0000
Vilana vs Sponsor: Diff=0.2043, 95%CI=-0.6793 to 1.0880, p=0.9665
Vilana vs Kye-shuan: Diff=0.2948, 95%CI=-0.5888 to 1.1785, p=0.8638
Vilana vs Czi-ti-4: Diff=-0.1259, 95%CI=-1.0095 to 0.7578, p=0.9961
Vilana vs Lastochka: Diff=0.1488, 95%CI=-0.7348 to 1.0324, p=0.9916
Zen vs Sponsor: Diff=0.2268, 95%CI=-0.6568 to 1.1105, p=0.9487
Zen vs Kye-shuan: Diff=0.3174, 95%CI=-0.5663 to 1.2010, p=0.8259
Zen vs Czi-ti-4: Diff=-0.1033, 95%CI=-0.9870 to 0.7803, p=0.9985
Zen vs Lastochka: Diff=0.1713, 95%CI=-0.7123 to 1.0550, p=0.9842
Sponsor vs Kye-shuan: Diff=0.0905, 95%CI=-0.7931 to 0.9742, p=0.9992
Sponsor vs Czi-ti-4: Diff=-0.3302, 95%CI=-1.2138 to 0.5535, p=0.8025
Sponsor vs Lastochka: Diff=-0.0555, 95%CI=-0.9392 to 0.8281, p=0.9999
Kye-shuan vs Czi-ti-4: Diff=-0.4207, 95%CI=-1.3043 to 0.4630, p=0.6139
Kye-shuan vs Lastochka: Diff=-0.1460, 95%CI=-1.0297 to 0.7376, p=0.9923
Czi-ti-4 vs Lastochka: Diff=0.2747, 95%CI=-0.6090 to 1.1583, p=0.8938

Dr-TP1, Drought, Time-point 1:

ANOVA Table...

Source of Variation	Sum of Squares	d.f.	Variance	F	p
Between Groups:	0.4600	5	0.0920	0.5309	0.7492
Within Groups:	2.0793	12	0.1733		
Total:	2.5393	17			

Post-hoc tests...

```
Vilana vs Zen: Diff=0.3362, 95%CI=-0.8054 to 1.4779, p=0.9128
Vilana vs Sponsor: Diff=-0.1580, 95%CI=-1.2996 to 0.9837, p=0.9966
Vilana vs Kye-shuan: Diff=0.0904, 95%CI=-1.0513 to 1.2320, p=0.9998
Vilana vs Czi-ti-4: Diff=-0.1028, 95%CI=-1.2444 to 1.0389, p=0.9996
Vilana vs Lastochka: Diff=-0.0170, 95%CI=-1.1586 to 1.1247, p=1.0000
Zen vs Sponsor: Diff=-0.4942, 95%CI=-1.6359 to 0.6474, p=0.6967
Zen vs Kye-shuan: Diff=-0.2459, 95%CI=-1.3875 to 0.8958, p=0.9751
Zen vs Czi-ti-4: Diff=-0.4390, 95%CI=-1.5806 to 0.7027, p=0.7842
Zen vs Lastochka: Diff=-0.3532, 95%CI=-1.4948 to 0.7885, p=0.8956
Sponsor vs Kye-shuan: Diff=0.2484, 95%CI=-0.8933 to 1.3900, p=0.9740
```

Tukey HSD Post-hoc Test:

Vilana vs Zen: Diff=0.3362, 95%CI=-0.8054 to 1.4779, p=0.9128
Vilana vs Sponsor: Diff=-0.1580, 95%CI=-1.2996 to 0.9837, p=0.9966
Vilana vs Kye-shuan: Diff=0.0904, 95%CI=-1.0513 to 1.2320, p=0.9998
Vilana vs Czi-ti-4: Diff=-0.1028, 95%CI=-1.2444 to 1.0389, p=0.9996
Vilana vs Lastochka: Diff=-0.0170, 95%CI=-1.1586 to 1.1247, p=1.0000
Zen vs Sponsor: Diff=-0.4942, 95%CI=-1.6359 to 0.6474, p=0.6967
Zen vs Kye-shuan: Diff=-0.2459, 95%CI=-1.3875 to 0.8958, p=0.9751
Zen vs Czi-ti-4: Diff=-0.4390, 95%CI=-1.5806 to 0.7027, p=0.7842
Zen vs Lastochka: Diff=-0.3532, 95%CI=-1.4948 to 0.7885, p=0.8956
Sponsor vs Kye-shuan: Diff=0.2484, 95%CI=-0.8933 to 1.3900, p=0.9740
Sponsor vs Czi-ti-4: Diff=0.0552, 95%CI=-1.0864 to 1.1969, p=1.0000
Sponsor vs Lastochka: Diff=0.1410, 95%CI=-1.0006 to 1.2827, p=0.9980
Kye-shuan vs Czi-ti-4: Diff=-0.1931, 95%CI=-1.3348 to 0.9485, p=0.9914
Kye-shuan vs Lastochka: Diff=-0.1073, 95%CI=-1.2490 to 1.0343, p=0.9995
Czi-ti-4 vs Lastochka: Diff=0.0858, 95%CI=-1.0558 to 1.2274, p=0.9998

Dr-TP2, Drought, Time-point 2:

ANOVA Table...

Source of Variation	Sum of Squares	d.f.	Variance	F	p
Between Groups:	15.2545	5	3.0509	2.4601	0.0935
Within Groups:	14.8815	12	1.2401		
Total:	30.1360	17			

Post-hoc tests...

```
Vilana vs Zen: Diff=-0.9911, 95%CI=-4.0453 to 2.0631, p=0.8763
Vilana vs Sponsor: Diff=-0.3774, 95%CI=-3.4316 to 2.6768, p=0.9980
Vilana vs Kye-shuan: Diff=-2.1198, 95%CI=-5.1740 to 0.9344, p=0.2539
Vilana vs Czi-ti-4: Diff=-2.3016, 95%CI=-5.3557 to 0.7526, p=0.1894
Vilana vs Lastochka: Diff=-2.1820, 95%CI=-5.2361 to 0.8722, p=0.2301
Zen vs Sponsor: Diff=0.6137, 95%CI=-2.4405 to 3.6679, p=0.9815
Zen vs Kye-shuan: Diff=-1.1287, 95%CI=-4.1829 to 1.9254, p=0.8092
Zen vs Czi-ti-4: Diff=-1.3105, 95%CI=-4.3647 to 1.7437, p=0.7039
Zen vs Lastochka: Diff=-1.1909, 95%CI=-4.2451 to 1.8633, p=0.7749
Sponsor vs Kye-shuan: Diff=-1.7424, 95%CI=-4.7966 to 1.3117, p=0.4378
```

Tukey HSD Post-hoc Test:

Vilana vs Zen: Diff=-0.9911, 95%CI=-4.0453 to 2.0631, p=0.8763
Vilana vs Sponsor: Diff=-0.3774, 95%CI=-3.4316 to 2.6768, p=0.9980
Vilana vs Kye-shuan: Diff=-2.1198, 95%CI=-5.1740 to 0.9344, p=0.2539
Vilana vs Czi-ti-4: Diff=-2.3016, 95%CI=-5.3557 to 0.7526, p=0.1894
Vilana vs Lastochka: Diff=-2.1820, 95%CI=-5.2361 to 0.8722, p=0.2301
Zen vs Sponsor: Diff=0.6137, 95%CI=-2.4405 to 3.6679, p=0.9815
Zen vs Kye-shuan: Diff=-1.1287, 95%CI=-4.1829 to 1.9254, p=0.8092
Zen vs Czi-ti-4: Diff=-1.3105, 95%CI=-4.3647 to 1.7437, p=0.7039
Zen vs Lastochka: Diff=-1.1909, 95%CI=-4.2451 to 1.8633, p=0.7749
Sponsor vs Kye-shuan: Diff=-1.7424, 95%CI=-4.7966 to 1.3117, p=0.4378
Sponsor vs Czi-ti-4: Diff=-1.9242, 95%CI=-4.9784 to 1.1300, p=0.3409
Sponsor vs Lastochka: Diff=-1.8046, 95%CI=-4.8588 to 1.2496, p=0.4031
Kye-shuan vs Czi-ti-4: Diff=-0.1817, 95%CI=-3.2359 to 2.8724, p=0.9999
Kye-shuan vs Lastochka: Diff=-0.0621, 95%CI=-3.1163 to 2.9920, p=1.0000
Czi-ti-4 vs Lastochka: Diff=0.1196, 95%CI=-2.9346 to 3.1738, p=1.0000

(H) Glyma.18G029000, Auxin transporter (*AUXT1*)

WW-TP1, Well-watered, Time-point 1:

ANOVA Table...

Source of Variation	Sum of Squares	d.f.	Variance	F	p
Between Groups:	0.1727	5	0.0345	0.6766	0.6494
Within Groups:	0.6127	12	0.0511		
Total:	0.7854	17			

Post-hoc tests...

Vilana vs Zen: Diff=-0.1784, 95%CI=-0.7981 to 0.4413, p=0.9199
Vilana vs Sponsor: Diff=-0.1820, 95%CI=-0.8017 to 0.4377, p=0.9138
Vilana vs Kye-shuan: Diff=-0.1360, 95%CI=-0.7557 to 0.4837, p=0.9731
Vilana vs Czi-ti-4: Diff=0.0194, 95%CI=-0.6003 to 0.6391, p=1.0000
Vilana vs Lastochka: Diff=-0.2475, 95%CI=-0.8672 to 0.3722, p=0.7583
Zen vs Sponsor: Diff=-0.0035, 95%CI=-0.6232 to 0.6162, p=0.9986
Zen vs Kye-shuan: Diff=0.0424, 95%CI=-0.5773 to 0.6621, p=0.9999
Zen vs Czi-ti-4: Diff=0.1978, 95%CI=-0.4219 to 0.8175, p=0.8833
Zen vs Lastochka: Diff=-0.0690, 95%CI=-0.6887 to 0.5507, p=0.9988
Sponsor vs Kye-shuan: Diff=0.0460, 95%CI=-0.5737 to 0.6657, p=0.9998

Tukey HSD Post-hoc Test:

Vilana vs Zen: Diff=-0.1784, 95%CI=-0.7981 to 0.4413, p=0.9199
Vilana vs Sponsor: Diff=-0.1820, 95%CI=-0.8017 to 0.4377, p=0.9138
Vilana vs Kye-shuan: Diff=-0.1360, 95%CI=-0.7557 to 0.4837, p=0.9731
Vilana vs Czi-ti-4: Diff=0.0194, 95%CI=-0.6003 to 0.6391, p=1.0000
Vilana vs Lastochka: Diff=-0.2475, 95%CI=-0.8672 to 0.3722, p=0.7583
Zen vs Sponsor: Diff=-0.0035, 95%CI=-0.6232 to 0.6162, p=0.9986
Zen vs Kye-shuan: Diff=0.0424, 95%CI=-0.5773 to 0.6621, p=0.9999
Zen vs Czi-ti-4: Diff=0.1978, 95%CI=-0.4219 to 0.8175, p=0.8833
Zen vs Lastochka: Diff=-0.0690, 95%CI=-0.6887 to 0.5507, p=0.9988
Sponsor vs Kye-shuan: Diff=0.0460, 95%CI=-0.5737 to 0.6657, p=0.9998
Sponsor vs Czi-ti-4: Diff=0.2013, 95%CI=-0.4184 to 0.8210, p=0.8758
Sponsor vs Lastochka: Diff=-0.0655, 95%CI=-0.6852 to 0.5542, p=0.9990
Kye-shuan vs Czi-ti-4: Diff=0.1553, 95%CI=-0.4644 to 0.7750, p=0.9533
Kye-shuan vs Lastochka: Diff=-0.1115, 95%CI=-0.7312 to 0.5082, p=0.9887
Czi-ti-4 vs Lastochka: Diff=-0.2668, 95%CI=-0.8865 to 0.3529, p=0.7011

WW-TP2, Well-watered, Time-point 2:

ANOVA Table...

Source of Variation	Sum of Squares	d.f.	Variance	F	p
Between Groups:	0.0807	5	0.0161	0.3529	0.8707
Within Groups:	0.5487	12	0.0457		
Total:	0.6294	17			

Post-hoc tests...

Vilana vs Zen: Diff=-0.1211, 95%CI=-0.7076 to 0.4654, p=0.9792
Vilana vs Sponsor: Diff=0.0835, 95%CI=-0.5030 to 0.6700, p=0.9961
Vilana vs Kye-shuan: Diff=-0.0556, 95%CI=-0.6421 to 0.5309, p=0.9994
Vilana vs Czi-ti-4: Diff=0.0124, 95%CI=-0.5741 to 0.5989, p=1.0000
Vilana vs Lastochka: Diff=0.0470, 95%CI=-0.5395 to 0.6335, p=0.9997
Zen vs Sponsor: Diff=0.2046, 95%CI=-0.3819 to 0.7911, p=0.8417
Zen vs Kye-shuan: Diff=0.0655, 95%CI=-0.5209 to 0.6520, p=0.9988
Zen vs Czi-ti-4: Diff=0.1335, 95%CI=-0.4530 to 0.7200, p=0.9686
Zen vs Lastochka: Diff=0.1681, 95%CI=-0.4184 to 0.7546, p=0.9212
Sponsor vs Kye-shuan: Diff=-0.1391, 95%CI=-0.7255 to 0.4474, p=0.9628

Tukey HSD Post-hoc Test:

Vilana vs Zen: Diff=-0.1211, 95%CI=-0.7076 to 0.4654, p=0.9792
Vilana vs Sponsor: Diff=0.0835, 95%CI=-0.5030 to 0.6700, p=0.9961
Vilana vs Kye-shuan: Diff=-0.0556, 95%CI=-0.6421 to 0.5309, p=0.9994
Vilana vs Czi-ti-4: Diff=0.0124, 95%CI=-0.5741 to 0.5989, p=1.0000
Vilana vs Lastochka: Diff=0.0470, 95%CI=-0.5395 to 0.6335, p=0.9997
Zen vs Sponsor: Diff=0.2046, 95%CI=-0.3819 to 0.7911, p=0.8417
Zen vs Kye-shuan: Diff=0.0655, 95%CI=-0.5209 to 0.6520, p=0.9988
Zen vs Czi-ti-4: Diff=0.1335, 95%CI=-0.4530 to 0.7200, p=0.9686
Zen vs Lastochka: Diff=0.1681, 95%CI=-0.4184 to 0.7546, p=0.9212
Sponsor vs Kye-shuan: Diff=-0.1391, 95%CI=-0.7255 to 0.4474, p=0.9628
Sponsor vs Czi-ti-4: Diff=-0.0711, 95%CI=-0.6576 to 0.5154, p=0.9982
Sponsor vs Lastochka: Diff=-0.0365, 95%CI=-0.6230 to 0.5500, p=0.9999
Kye-shuan vs Czi-ti-4: Diff=0.0679, 95%CI=-0.5185 to 0.6544, p=0.9985
Kye-shuan vs Lastochka: Diff=0.1026, 95%CI=-0.4839 to 0.6890, p=0.9900
Czi-ti-4 vs Lastochka: Diff=0.0346, 95%CI=-0.5519 to 0.6211, p=0.9999

Dr-TP1, Drought, Time-point 1:

ANOVA Table...

Source of Variation	Sum of Squares	d.f.	Variance	F	p
Between Groups:	4.3176	5	0.8635	5.5220	0.0072
Within Groups:	1.8766	12	0.1564		
Total:	6.1942	17			

Post-hoc tests...

```
Vilana vs Zen: Diff=-0.2647, 95%CI=-1.3493 to 0.8198, p=0.9581
Vilana vs Sponsor: Diff=1.0900, 95%CI=0.0054 to 2.1745, p=0.0486
Vilana vs Kye-shuan: Diff=0.6421, 95%CI=-0.4424 to 1.7267, p=0.4011
Vilana vs Czi-ti-4: Diff=0.0491, 95%CI=-1.0354 to 1.1337, p=1.0000
Vilana vs Lastochka: Diff=-0.2179, 95%CI=-1.3024 to 0.8667, p=0.9815
Zen vs Sponsor: Diff=1.3547, 95%CI=0.2701 to 2.4392, p=0.0122
Zen vs Kye-shuan: Diff=0.9068, 95%CI=-0.1777 to 1.9914, p=0.1232
Zen vs Czi-ti-4: Diff=0.3139, 95%CI=-0.7707 to 1.3984, p=0.9183
Zen vs Lastochka: Diff=0.0468, 95%CI=-1.0377 to 1.1314, p=1.0000
Sponsor vs Kye-shuan: Diff=-0.4478, 95%CI=-1.5324 to 0.6367, p=0.7338
```

Tukey HSD Post-hoc Test:

Vilana vs Zen: Diff=-0.2647, 95%CI=-1.3493 to 0.8198, p=0.9581
Vilana vs Sponsor: Diff=1.0900, 95%CI=0.0054 to 2.1745, p=0.0486
Vilana vs Kye-shuan: Diff=0.6421, 95%CI=-0.4424 to 1.7267, p=0.4011
Vilana vs Czi-ti-4: Diff=0.0491, 95%CI=-1.0354 to 1.1337, p=1.0000
Vilana vs Lastochka: Diff=-0.2179, 95%CI=-1.3024 to 0.8667, p=0.9815
Zen vs Sponsor: Diff=1.3547, 95%CI=0.2701 to 2.4392, p=0.0122
Zen vs Kye-shuan: Diff=0.9068, 95%CI=-0.1777 to 1.9914, p=0.1232
Zen vs Czi-ti-4: Diff=0.3139, 95%CI=-0.7707 to 1.3984, p=0.9183
Zen vs Lastochka: Diff=0.0468, 95%CI=-1.0377 to 1.1314, p=1.0000
Sponsor vs Kye-shuan: Diff=-0.4478, 95%CI=-1.5324 to 0.6367, p=0.7338
Sponsor vs Czi-ti-4: Diff=-1.0408, 95%CI=-2.1254 to 0.0438, p=0.0627
Sponsor vs Lastochka: Diff=-1.3078, 95%CI=-2.3924 to -0.2233, p=0.0156
Kye-shuan vs Czi-ti-4: Diff=-0.5930, 95%CI=-1.6775 to 0.4916, p=0.4802
Kye-shuan vs Lastochka: Diff=-0.8600, 95%CI=-1.9445 to 0.2246, p=0.1548
Czi-ti-4 vs Lastochka: Diff=-0.2670, 95%CI=-1.3516 to 0.8175, p=0.9566

Dr-TP2, Drought, Time-point 2:

ANOVA Table...

Source of Variation	Sum of Squares	d.f.	Variance	F	p
Between Groups:	4.6744	5	0.9349	2.5456	0.0858
Within Groups:	4.4070	12	0.3673		
Total:	9.0814	17			

Post-hoc tests...

```
Vilana vs Zen: Diff=-1.1830, 95%CI=-2.8451 to 0.4790, p=0.2331
Vilana vs Sponsor: Diff=0.5086, 95%CI=-1.1534 to 2.1707, p=0.8996
Vilana vs Kye-shuan: Diff=-0.4261, 95%CI=-2.0882 to 1.2359, p=0.9489
Vilana vs Czi-ti-4: Diff=-0.1144, 95%CI=-1.7764 to 1.5477, p=0.9999
Vilana vs Lastochka: Diff=-0.2650, 95%CI=-1.9271 to 1.3970, p=0.9934
Zen vs Sponsor: Diff=1.6917, 95%CI=0.0296 to 3.3537, p=0.0452
Zen vs Kye-shuan: Diff=0.7569, 95%CI=-0.9051 to 2.4190, p=0.6538
Zen vs Czi-ti-4: Diff=1.0687, 95%CI=-0.5934 to 2.7307, p=0.3218
Zen vs Lastochka: Diff=0.9180, 95%CI=-0.7441 to 2.5800, p=0.4701
Sponsor vs Kye-shuan: Diff=-0.9347, 95%CI=-2.5968 to 0.7273, p=0.4520
```

Tukey HSD Post-hoc Test:

Vilana vs Zen: Diff=-1.1830, 95%CI=-2.8451 to 0.4790, p=0.2331
Vilana vs Sponsor: Diff=0.5086, 95%CI=-1.1534 to 2.1707, p=0.8996
Vilana vs Kye-shuan: Diff=-0.4261, 95%CI=-2.0882 to 1.2359, p=0.9489
Vilana vs Czi-ti-4: Diff=-0.1144, 95%CI=-1.7764 to 1.5477, p=0.9999
Vilana vs Lastochka: Diff=-0.2650, 95%CI=-1.9271 to 1.3970, p=0.9934
Zen vs Sponsor: Diff=1.6917, 95%CI=0.0296 to 3.3537, p=0.0452
Zen vs Kye-shuan: Diff=0.7569, 95%CI=-0.9051 to 2.4190, p=0.6538
Zen vs Czi-ti-4: Diff=1.0687, 95%CI=-0.5934 to 2.7307, p=0.3218
Zen vs Lastochka: Diff=0.9180, 95%CI=-0.7441 to 2.5800, p=0.4701
Sponsor vs Kye-shuan: Diff=-0.9347, 95%CI=-2.5968 to 0.7273, p=0.4520
Sponsor vs Czi-ti-4: Diff=-0.6230, 95%CI=-2.2851 to 1.0391, p=0.8005
Sponsor vs Lastochka: Diff=-0.7737, 95%CI=-2.4357 to 0.8884, p=0.6344
Kye-shuan vs Czi-ti-4: Diff=0.3117, 95%CI=-1.3503 to 1.9738, p=0.9863
Kye-shuan vs Lastochka: Diff=0.1611, 95%CI=-1.5010 to 1.8231, p=0.9994
Czi-ti-4 vs Lastochka: Diff=-0.1507, 95%CI=-1.8127 to 1.5114, p=0.9995
